# Integrative multi-omics analysis identifies VCAN and ESM1 as candidate cell-type-associated biomarkers of NET-related glomerular injury in diabetic kidney disease

**DOI:** 10.1080/0886022X.2026.2707003

**Published:** 2026-08-06

**Authors:** Yufeng Liang, Chenlun Li, Binsan Huang, Azhen Wang, Meilan Qiu, Juan Lu, Jianxin Wan

**Affiliations:** a Department of Nephrology, The Second Hospital of Longyan, Fujian, China; b Department of Nephrology, National Regional Medical Center, Binhai Campus of the First Affiliated Hospital, Fujian Medical University, Fuzhou, China; c Department of Nephrology, Blood Purification Research Center, The First Affiliated Hospital, Fujian Medical University, Fuzhou, China; d Fujian Clinical Research Center for Metabolic Chronic Kidney Disease, The First Affiliated Hospital, Fujian Medical University, Fuzhou, China

**Keywords:** Diabetic kidney disease, neutrophil extracellular traps, VCAN, ESM1, glomerular injury

## Abstract

Diabetic kidney disease (DKD) involves complex inflammatory and microvascular injury, but cell-specific molecular signatures linked to neutrophil extracellular traps (NETs) remain unclear. We aimed to identify NET-associated biomarkers of glomerular injury in DKD. Bulk glomerular transcriptomic datasets were analyzed using differential expression, weighted gene co-expression network analysis, and machine learning (LASSO and random forest). Single-cell RNA sequencing (scRNA-seq) defined cell-specific expression and intercellular communication. Candidates were evaluated in independent cohorts, *in vitro* high-glucose co-culture datasets, and db/db mice. Exploratory in silico drug-screening was also performed. VCAN and ESM1 emerged as core candidate genes. In bulk cohorts, VCAN was upregulated and ESM1 downregulated, yielding strong combined diagnostic performance. scRNA-seq revealed ESM1 decreases predominantly in glomerular endothelial cells, while VCAN increases in mesangial cells. NET-related activity enriched primarily in the endothelium, whereas VCAN-expressing cells associated with matrix remodeling. *In vitro*, ESM1 showed early endothelial-predominant decreases under injury. In db/db mice, renal VCAN and NET markers were elevated, while ESM1 levels were reduced and correlated with albuminuria severity. Molecular docking identified potential targeting compounds. In conclusion, VCAN and ESM1 are promising biomarkers reflecting distinct, cell-type-enriched patterns of NET-related glomerular injury in DKD, supporting a dual-track model of endothelial dysfunction and paracrine matrix remodeling.

## 1. Introduction

Diabetic kidney disease (DKD) remains the primary driver of end-stage renal disease globally, affecting approximately 30% of diabetic patients and imposing substantial socioeconomic burdens [1]. Current diagnostic methods, relying on albuminuria and estimated glomerular filtration rate (eGFR), often fail to capture early pathological changes, particularly in patients with normoalbuminuric DKD [2,3]. Consequently, identifying molecular biomarkers that reflect specific underlying mechanisms is critical for improving early detection and intervention.

The pathogenesis of DKD involves a complex interplay of metabolic dysregulation, hemodynamic perturbations, and inflammatory activation [4]. Although traditionally viewed as metabolically driven, DKD is increasingly recognized as an inflammatory condition where immune activation plays a central role. Innate immune mechanisms, in particular, promote progressive glomerular injury, with inflammatory mediators strongly correlating with declining renal function [4]. Among these, neutrophil extracellular traps (NETs)—web-like chromatin structures originally described in host defense—are now recognized as candidate key mediators of sterile inflammation in renal pathology [5–7]. Beyond antimicrobial functions, NETs have been causally implicated in tissue damage across diverse noninfectious inflammatory and autoimmune conditions [8,9].

Evidence linking NET formation to DKD progression has grown significantly. Studies from 2023 and 2024 report abundant accumulation of NET components in diabetic kidney biopsies compared to controls [10,11]. Mechanistically, chronic hyperglycemia and advanced glycation end products (AGEs) appear to prime neutrophils for enhanced NET release, creating a pathological cycle that amplifies injury. While histological studies place NET deposition primarily around glomerular capillaries, biochemical analyses confirm elevated circulating and urinary NET products [8,12]. Thus, NET biomarkers may serve as both pathological effectors and indicators of renal dysfunction.

The glomerular filtration barrier, comprising fenestrated endothelium, glomerular basement membrane, and podocytes, represents a primary target of diabetic injury. Glomerular endothelial cells (GECs), constituting a significant portion of the glomerular volume, act as the first line of defense against circulating inflammatory stimuli [13,14]. scRNA-seq studies have documented profound phenotypic alterations in diabetic GECs, including fenestral loss and metabolic reprogramming. Experimental models suggest that NET-endothelial interactions trigger injury through oxidative stress and barrier disruption [7,10]. However, the specific molecular signatures distinguishing NET-mediated glomerular injury from other inflammatory cascades remain ill-defined, limiting the design of targeted therapies.

Systems biology approaches offer robust tools for dissecting these complex networks [15]. Combining high-throughput sequencing with computational methods allows for comprehensive molecular profiling. Machine learning algorithms, particularly when integrated with network analyses, can uncover disease-associated patterns often missed by traditional approaches [16]. Despite progress, several knowledge gaps remain. First, the specific cellular origins of NET-responsive genes within the glomerulus are not fully characterized. Second, the temporal dynamics of gene expression—distinguishing adaptive from maladaptive responses—require systematic examination at single-cell resolution. Third, existing studies often focus on single molecules rather than coordinated networks. Finally, the translational potential of computational targets requires rigorous multi-cohort and experimental validation.

To address these issues, we employed a multi-tier analytical strategy. We utilized weighted gene co-expression network analysis (WGCNA) and dual machine learning algorithms to screen glomerular transcriptomic datasets. Using scRNA-seq, we mapped the cellular expression and dynamics of candidate genes. We further established clinical relevance through multi-cohort validation and experimental verification in db/db mice, concluding with computational drug repurposing analysis.

## 2. Materials and methods

### 2.1. Acquisition and preprocessing of bulk transcriptomic data

Raw microarray datasets GSE30528 [17] and GSE96804 [18] were retrieved from the Gene Expression Omnibus (GEO) database (accessed on 15 January 2024). Inclusion criteria were established as follows: (1) data generated on microarray platforms; (2) samples derived from human glomerular tissue; (3) study design encompassing DKD patients and healthy controls. The merged cohort comprised 83 samples (50 DKD cases and 33 controls). For each dataset, probe identifiers were annotated to official gene symbols according to the corresponding platform annotation file. When multiple probes mapped to the same gene symbol, their average expression value was used. To minimize technical variability, raw expression data underwent log2 transformation followed by quantile normalization, and batch effects were corrected using the ComBat algorithm, ensuring cross-platform comparability (Figure S1).

### 2.2. Single-cell RNA sequencing (scRNA-seq) analysis

To investigate cellular heterogeneity, scRNA-seq data (GSE131882) [19] comprising three early-stage human DKD specimens and three healthy controls were analyzed. Standard quality control (QC) was performed using Seurat. Filtering criteria included: >3 genes/cell; >200 feature counts; <4,000 features (to exclude multiplets); and <2% mitochondrial gene content. Doublets were removed *via* DoubletFinder (v2.0.4) [20], and ambient RNA was corrected using decontX (v1.2.0) [21]. The final dataset contained 19,897 nuclei. Data integration was achieved using the Harmony algorithm (Figure S1).

### 2.3. Construction of neutrophil extracellular Trap-Related gene set and single-cell scoring

A curated list of 214 unique neutrophil extracellular trap-related genes (NRGs) was compiled from three complementary sources: (1) 137 genes identified by Wu et al. [15] in renal ischemia-reperfusion injury; (2) 69 genes from pan-cancer NET signature studies [22]; (3) 89 genes from the GeneCards database (search term: ‘Neutrophil Extracellular Traps’) with relevance scores ≥20. After removing duplicates, 214 unique NRGs were retained. To generate a disease-contextualized reference set, these 214 NRGs were intersected with DKD-associated genes retrieved from GeneCards (search term: ‘Diabetic Kidney Disease’). Coincidentally, this intersection yielded exactly 89 DKD-contextualized NRGs (DKD-NRGs; full list in Supplementary Table S5). It should be noted that these two 89-gene sets are derived from different search strategies and are not identical. Both the full 214-gene set and the DKD-NRG subset were used in parallel for the downstream GSEA and AUCell analyses.NETs activity scores at the single-cell level were computed using the AUCell algorithm. To evaluate the activity of these NRGs in the DKD single-cell atlas, we performed cell scoring using the R package AUCell (version 1.26.0). AUCell identifies cells with active gene sets (e.g., signatures, gene modules) in scRNA-seq data. Specifically, it calculates the ‘Area Under the Curve’ (AUC) to assess whether a critical subset of the input gene set is enriched within the expressed genes of each individual cell. By analyzing the distribution of AUC scores across all cells, we explored the relative expression and activation status of the NRG signature across different cell populations.

### 2.4. Validation using independent external and In vitro transcriptomic datasets

To further validate the diagnostic performance of the identified hub genes in clinical settings, an independent external cohort (GSE142025) was downloaded from the GEO database. This dataset comprises 27 DKD samples (including 6 early-stage and 21 advanced-stage DKD) and nine healthy controls. The expression data were processed using the same methods as the training cohort. ROC curve analysis was performed consistently with the training model to evaluate the diagnostic value of VCAN and ESM1. To validate the cell-type-associated expression, temporal expression dynamics, and pathological responses of the identified biomarkers, relevant *in vitro* transcriptomic datasets were acquired from the Gene Expression Omnibus (GEO) database. Specifically, publicly available gene expression profiles of human glomerular endothelial cells (GSE220227) and podocytes (GSE220228) co-cultured under high-glucose conditions for 48 and 96 h were downloaded. The normalized expression matrices from these datasets were extracted to evaluate the expression levels of ESM1 and VCAN across different cell types, time points, and stimulation conditions. To further test whether VCAN and ESM1 respond directly to NET exposure, we analyzed GSE189875, a publicly available transcriptomic dataset of human endothelial cells treated with or without isolated NETs for 12 h (*n* = 5 per group) to assess whether short-term direct NET exposure alters VCAN or ESM1 expression.

### 2.5. Differential expression and functional enrichment analyses

Differential expression analysis was conducted using the limma package coupled with empirical Bayes methods. Differentially expressed genes (DEGs) were defined by adjusted *p*-value <0.05 and |log2-fold change (FC)| >1.0. Functional annotation was performed with the clusterProfiler package, encompassing Gene Ontology (GO) and Kyoto Encyclopedia of Genes and Genomes (KEGG) pathway analyses. Gene Set Enrichment Analysis (GSEA) was executed with 1,000 permutations to identify significantly enriched biological pathways (Table S1–3). To assess the robustness of NET-related enrichment to gene-set definition, GSEA was additionally performed on the DKD-NRG subset and on three standardized NET-formation gene sets (KEGG, GO, and WikiPathways) (Supplementary Table S6). In parallel, a NET CoreGene Score was computed from ten canonical NET genes (PADI4, MPO, ELANE, CTSG, GSDMD, CYBB, AGER, VWF, TLR2, and F3) and compared between DKD and control samples using the Wilcoxon rank-sum test.

### 2.6. Weighted gene co-expression network analysis

Co-expression networks were constructed using Weighted Gene Co-expression Network Analysis (WGCNA). Soft-thresholding power (β) was selected to achieve a scale-free topology fitting index (R^2^) >0.8. Modules were identified through dynamic tree-cutting algorithms (deepSplit = 2, minimum module size = 50), and module preservation statistics were calculated to assess network robustness. Module-trait relationships were then evaluated to identify the module most strongly associated with the DKD phenotype. Genes from the key disease-associated module were intersected with DEGs for subsequent feature selection.

### 2.7. Machine learning-based feature selection

A dual machine learning strategy was employed for diagnostic biomarker screening. Initially, Least Absolute Shrinkage and Selection Operator (LASSO) regression was performed using the glmnet package with 10-fold cross-validation. Subsequently, features were ranked by mean decrease in Gini index using random forest algorithms (randomForest package, ntree = 100). Diagnostic performance was evaluated through receiver operating characteristic (ROC) curve analysis (Table S4). For the combined diagnostic model of ESM1 and VCAN, a binary logistic regression model was constructed in the merged cohort, and the area under the ROC curve (AUC) was calculated.

### 2.8. Immune infiltration analysis

The CIBERSORT algorithm was applied to estimate relative proportions of 22 immune cell types (1,000 permutations). Spearman rank correlation analysis was employed to evaluate associations between gene expression and immune cell abundance.

### 2.9. Experimental validation

To further validate our findings at the mRNA and protein levels *in vivo*, C57BL/KsJ-db/db male mice (*n* = 6) and age-matched db/m control littermates (*n* = 6) were utilized. Mice were housed under specific pathogen-free (SPF) conditions with a 12-h light/dark cycle and *ad libitum* access to food and water. At 20 weeks of age, diabetic phenotypes were confirmed through the monitoring of body weight, fasting blood glucose, and urinary albumin levels. All experimental procedures were conducted in accordance with institutional animal care guidelines and the ARRIVE guidelines, and were approved by the institutional committee (Approval Number: FJMU IACUC 2020-0104). A completed ARRIVE guidelines checklist is provided as a supplementary file.

At the end of the experiment, mice were deeply anesthetized with an intraperitoneal injection of sodium pentobarbital (50 mg/kg body weight) and euthanized by cervical dislocation. All euthanasia procedures were performed in strict accordance with the American Veterinary Medical Association (AVMA) Guidelines for the Euthanasia of Animals. Following euthanasia, kidney tissues, blood, and urine samples were harvested. For mRNA quantification, total RNA was extracted from kidney tissues and subjected to quantitative real-time PCR (qRT-PCR) (Table S7). Relative expression was calculated using the 2-ΔΔCt method with GAPDH as the internal reference. For protein localization and expression analysis, immunohistochemistry (IHC) was performed. Formalin-fixed, paraffin-embedded tissue sections (4 μm) were deparaffinized, subjected to antigen retrieval, and incubated with primary antibodies against myeloperoxidase (MPO; R&D Systems, AF3667, 1:200), Cit-H3 (Abcam, ab5103, 1:100), Versican (VCAN; ImmunoWay, YT4874, 1:150), and Endothelial cell-specific molecule 1 (ESM1; ImmunoWay, YT7820, 1:100). Staining intensity was quantified using ImageJ software (NIH, Bethesda, MD, USA) by two independent observers blinded to group allocation. Additionally, the plasma concentrations of ESM1 and MPO-NETs in mice were quantified using specific enzyme-linked immunosorbent assay (ELISA) kits (ESM1: Cat. No. E-EL-M3106, Elabscience; MPO-NETs: Cat. No. AB-B57554A, Ab-mart) in strict accordance with the manufacturers’ instructions.

### 2.10. Deep learning-driven drug prediction

The GraphBAN (Graph-based Bilinear Attention Network) model was utilized to predict potential therapeutic compounds targeting VCAN and ESM1. Candidate drugs were screened from the ZINC-250K database, filtered by Lipinski’s rule of five and pan-assay interference compounds (PAINS) criteria. Compounds with high prediction probabilities (>0.5) underwent molecular docking *via* CB-DOCK2, with protein structures sourced from AlphaFold (Table S8).

### 2.11. Statistics

Statistical analyses were primarily performed using R software (version 4.4.2). For experimental data (e.g., animal phenotype, qRT-PCR, and IHC), data distribution was assessed using the Shapiro-Wilk test. Continuous variables are presented as mean ± standard deviation (SD) for normally distributed data or median with interquartile range (IQR) for non-normally distributed data. Comparisons between two groups were conducted using the unpaired Student’s t-test or the Mann–Whitney U-test, as appropriate. For high-throughput sequencing data, *p*-values were adjusted for multiple testing using the Benjamini–Hochberg method to control the False Discovery Rate (FDR). Correlation analyses were performed using Pearson’s or Spearman’s correlation coefficients based on data distribution. A two-sided *p*-value < 0.05 was considered statistically significant.

## 3. Results

### 3.1. Transcriptomic analysis identifies DEGs and pathway enrichment in DKD

**Figure 1.**
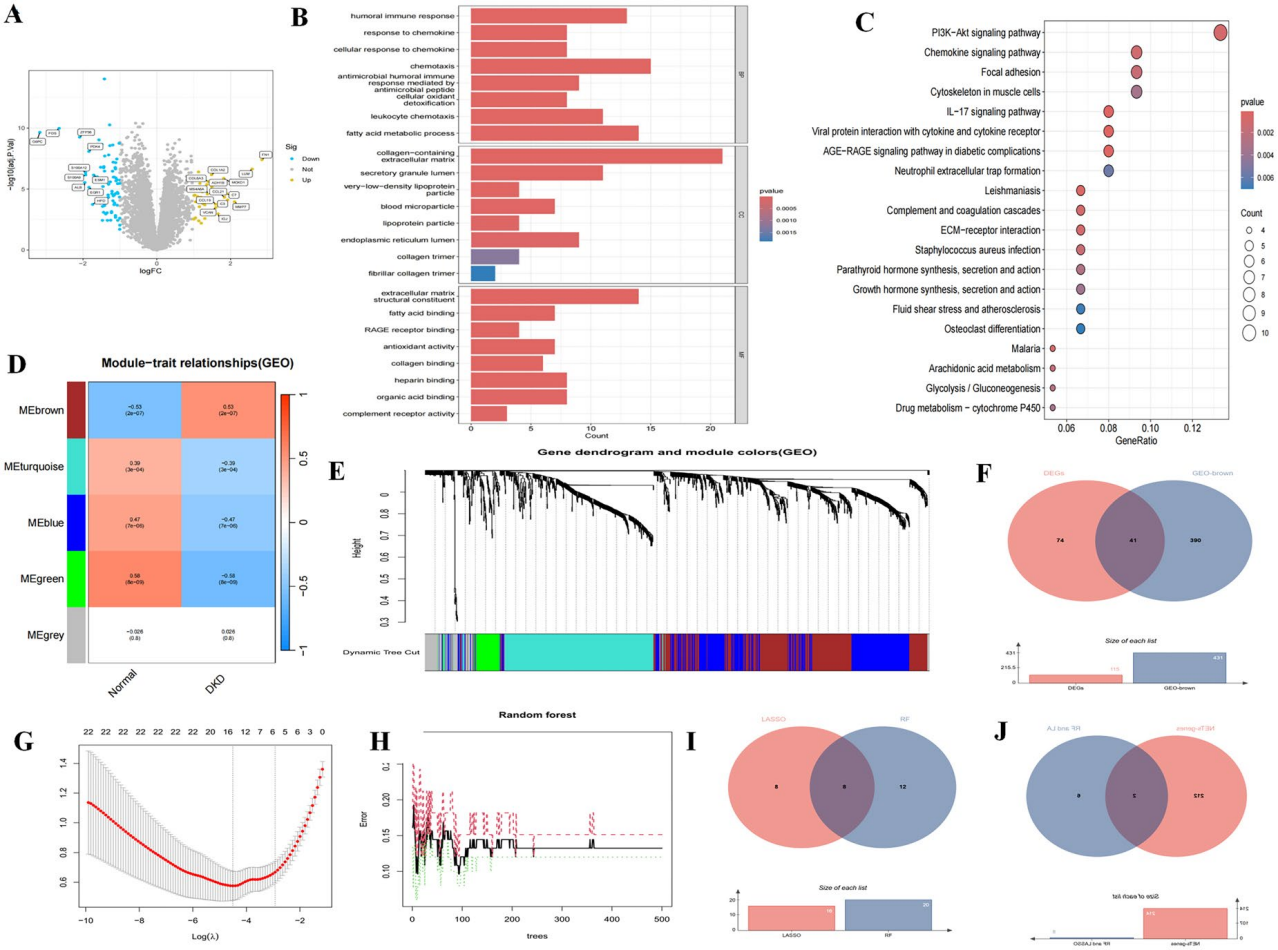
Integrated bioinformatics analysis identifies VCAN and ESM1 as core feature genes. (A) Volcano plot of 115 DEGs between DKD and controls. (B) GO enrichment analysis of the 115 DEGs. (C) KEGG pathway enrichment analysis. (D) WGCNA module-trait relationship heatmap. (E) Gene dendrogram with module assignments. (F) Venn diagram of DEGs and brown module genes. (G) LASSO regression coefficient profiles. (H) Random forest feature importance. (I) Venn diagram of LASSO and random forest outputs. (J) Venn diagram showing the overlap between the candidate genes identified in (I) and NET-related genes. DEGs: differentially expressed genes; GO: Gene Ontology; KEGG: Kyoto Encyclopedia of Genes and Genomes; WGCNA: weighted gene co-expression network analysis; LASSO: Least Absolute Shrinkage and Selection Operator.

We integrated the GSE30528 and GSE96804 datasets, encompassing 50 DKD samples and 33 controls, and applied the ComBat algorithm for batch correction. Principal component analysis revealed clear separation between DKD and control samples, with the first two principal components accounting for over 50% of the total variance. We identified 115 differentially expressed genes (DEGs) (Figure 1(A)), including key genes closely associated with DKD pathogenesis such as VCAN, ESM1, and several notable inflammatory and metabolic markers (the full list of DEGs is provided in Table S1). GO enrichment analysis of the 115 DEGs revealed significant enrichment in three main dimensions: metabolic stress (e.g., fatty acid metabolic process, lipoprotein particle, and RAGE receptor binding), immune and inflammatory activation (e.g., leukocyte chemotaxis and complement receptor activity), and extracellular matrix remodeling (e.g., collagen-containing extracellular matrix) (Figure 1(B)). KEGG pathway analysis showed notable enrichment in the PI3K-Akt signaling, IL-17 signaling, and NET formation pathways (Figure 1(C)), highlighting the contribution of innate immunity to DKD pathogenesis. To contextualize this signal within DKD and to test its sensitivity to gene-set definition, we performed complementary GSEA. A CoreGene Score derived from 10 canonical NET genes was significantly elevated in DKD (*p* < 0.001), providing the primary tissue-level evidence of genuine NET-related activity. Consistently, a DKD-contextualized NRG set was also enriched in DKD glomeruli (adjusted *p* = 0.023); because this set was constructed by intersecting with DKD-associated genes; however, its enrichment should be regarded as confirmatory rather than independent evidence. By contrast, the three standardized NET-formation gene sets (KEGG, GO, and WikiPathways) did not reach significance (Supplementary Table S6). Because glomerular tissue represents a target rather than a source of NET formation, this lack of enrichment for canonical ‘NET-formation’ sets is biologically expected.

### 3.2. WGCNA and machine learning algorithms identify core feature genes

WGCNA identified the brown module (431 genes) as having the strongest correlation with the DKD phenotype (Figure 1(D–E)). Intersecting the 115 DEGs with brown module genes yielded 41 candidate core genes (Figure 1(F)). LASSO regression and random forest algorithms identified 16 and 20 robust feature genes, respectively (Figure 1(G–H)), with eight consensus genes emerging from their intersection (Figure 1(I)). Comparison against the 214 NET-related genes pinpointed VCAN and ESM1 as core features (Figure 1(J)), suggesting their potential involvement in NET-mediated DKD pathogenesis.

### 3.3. Single-cell RNA sequencing reveals endothelial-enriched NETs activation and distinct molecular signatures

**Figure 2.**
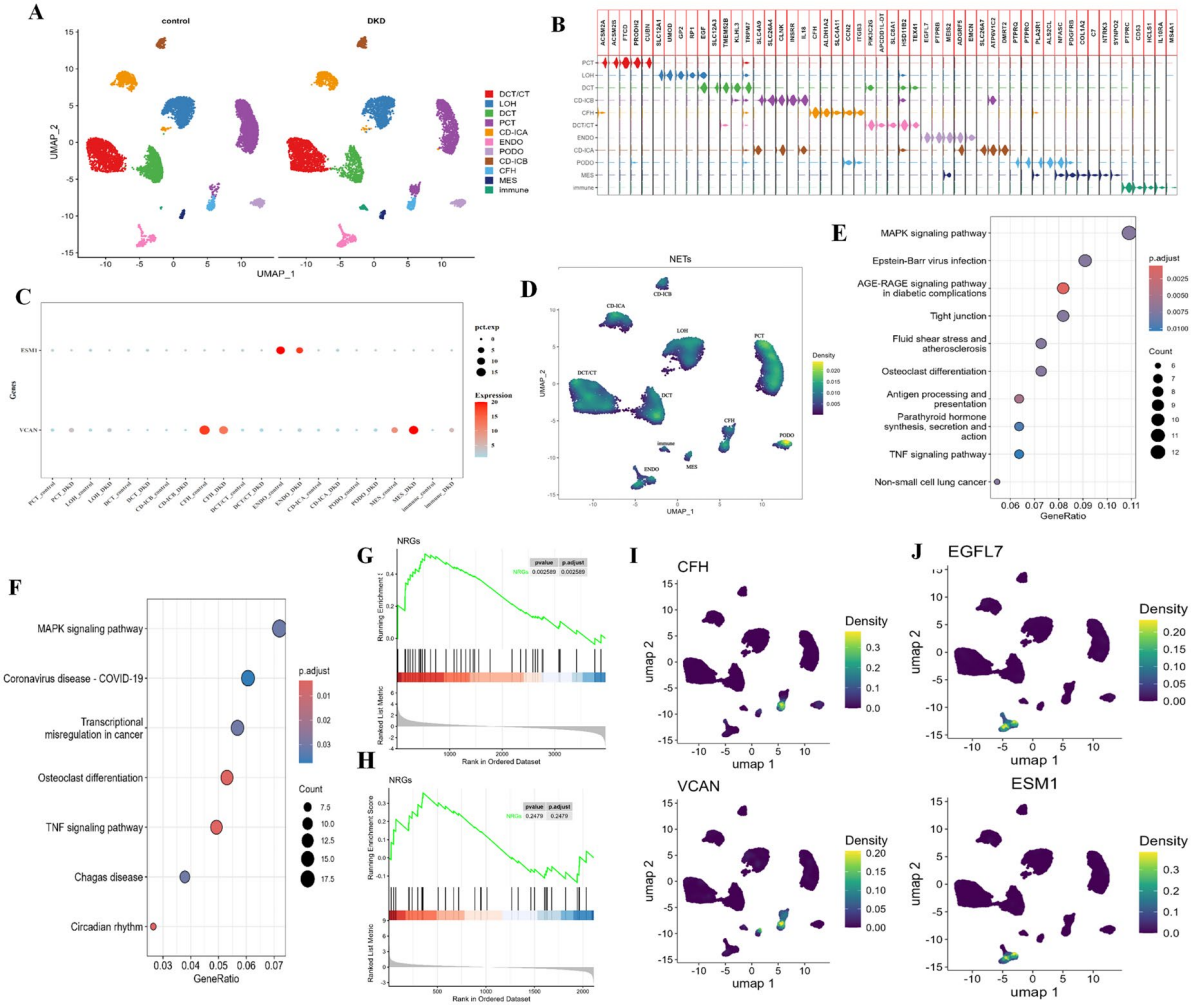
Single-cell atlas reveals NETs activation and cell type-specific expression of VCAN and ESM1 in DKD. (A, B) UMAP visualization (A) and marker gene expression (B) of 11 renal cell clusters from control and early DKD samples (*n* = 19,897 cells). (C) Dot plots showing the downregulation of ESM1 in endothelial cells (ENDO), and the upregulation of VCAN in mesangial cells (MES), but its downregulation in CFH-positive parietal epithelial cells (CFH^+^ PECs) in DKD. (D) UMAP projection of NETs activity scores. (E, F) KEGG pathway analysis of upregulated genes in DKD endothelial cells (E) and CFH^+^ PECs (F). (G, H) GSEA of NETs-related signatures in endothelial cells (G) versus CFH^+^ PECs (H). (I, J) Feature plots validating the co-localization of VCAN with CFH (I) and ESM1 with EGFL7 (J). UMAP: Uniform Manifold Approximation and Projection; ENDO: endothelial cells; MES: mesangial cells; PECs: parietal epithelial cells; GSEA: Gene Set Enrichment Analysis.

To dissect the cellular heterogeneity and spatial compartmentalization of early DKD, we integrated 19,897 high-quality single-cell transcriptomes and identified 11 major cell subpopulations through unsupervised clustering (Figure 2(A–B)). Differential expression analysis uncovered distinct cell-type-associated patterns: ESM1(Endocan, a secreted proteoglycan crucial for regulating vascular permeability and leukocyte extravasation) was specifically downregulated in DKD endothelial cells (ENDO), while VCAN (a major extracellular matrix proteoglycan driving cell adhesion and tissue remodeling) was significantly upregulated in mesangial cells (MES) but downregulated in Complement Factor H (CFH)-positive parietal epithelial cells (CFH+ PECs, a subpopulation characterized by the expression of CFH, a key negative regulator of the alternative complement pathway) in the DKD group (Figure 2(C)). NRG signature analysis showed significant enrichment of NET activity scores within the endothelial compartment (Figure 2(D)). To quantitatively evaluate this distribution, we assessed the NETs scores across all cell populations, revealing that endothelial cells (ENDO) and podocytes (PODO) exhibited the highest overall scores, with the ENDO population showing a noticeable elevation in the DKD group (Supplementary Figure S2). Although podocytes also showed high baseline NET scores, ESM1 was nearly undetectable in podocytes and podocyte VCAN was downregulated in the co-culture model (Figure 5(I)), supporting endothelial cells—rather than podocytes—as the principal cellular focus of the proposed model. To validate these enrichment scores at the single-gene level, we analyzed the expression of canonical NET-responsive genes and receptors, which confirmed cell-type-associated activation profiles, such as VWF in ENDO and AGER/RAGE in CFH^+^ PECs (Supplementary Figure S3). These findings implicate the vasculature as the primary target of NET-associated signaling. Pathway enrichment indicated that upregulated genes in DKD endothelial cells were involved in tight junction, fluid shear stress, and MAPK/TNF pathways (Figure 2(E)), suggesting structural compromise. Although CFH^+^ PECs also displayed inflammatory activation (Figure 2(F)), GSEA revealed a critical distinction: endothelial cells exhibited significant NET gene set enrichment (*p* = 0.0026, Figure 2(G)), whereas CFH^+^ PECs did not (*p* = 0.2479, Figure 2(H)). Feature plots confirmed VCAN-CFH overlap (Figure 2(I)) and ESM1-EGFL7(an endothelial-restricted secreted factor that regulates vascular development and integrity) co-expression (Figure 2(J)). Collectively, these data suggest that direct NET activation signals converge on glomerular endothelial cells, while VCAN upregulation in other compartments may occur independently of direct NET gene signature enrichment.

### 3.4. Subcluster analysis reveals cellular heterogeneity and pseudotime dynamics

**Figure 3.**
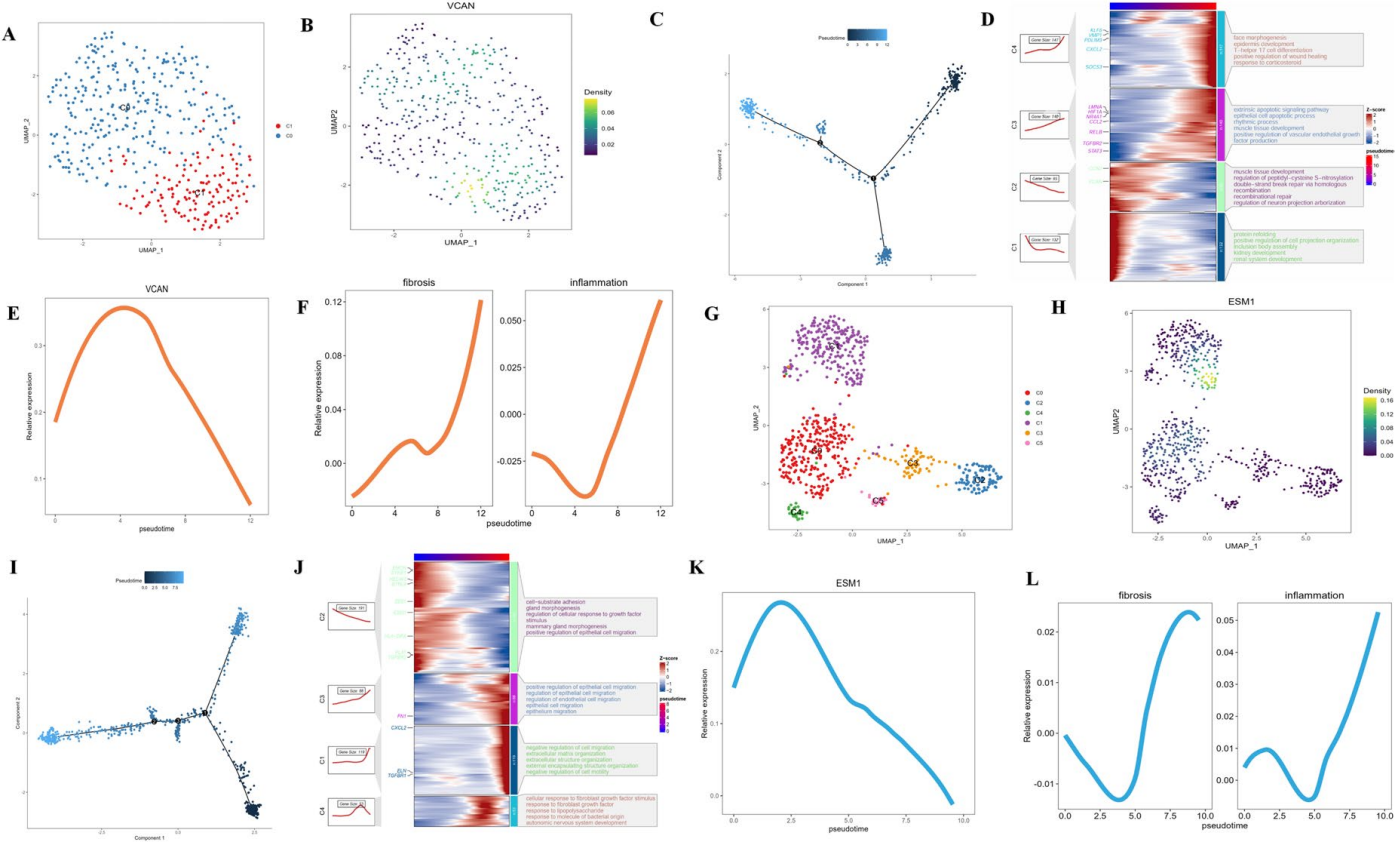
Subcluster heterogeneity and pseudotime trajectory analysis of VCAN and ESM1. (A) UMAP plot of CFH+PEC subclusters. (B) Density plot showing VCAN expression in CFH+PEC cells. (C) Pseudotime trajectory of CFH+PEC cells colored by pseudotime. (D) Heatmap showing dynamic gene expression patterns along pseudotime with associated biological processes. (E) VCAN relative expression along pseudotime. (F) Pseudotime dynamics of fibrosis and inflammation signature scores in CFH+PEC cells. (G) UMAP plot of ENDO subclusters. (H) Density plot showing ESM1 expression in ENDO cells. (I) Pseudotime trajectory of ENDO cells colored by pseudotime. (J) Heatmap showing dynamic gene expression patterns along pseudotime with associated biological processes. (K) ESM1 relative expression along pseudotime. (L) Pseudotime dynamics of fibrosis and inflammation signature scores in ENDO cells.

Subcluster analysis identified distinct subpopulations within CFH^+^PEC and ENDO cells. To better understand the biological context prior to trajectory inference, we evaluated the cell numbers and functional characteristics of these subclusters (Supplementary Figure S3). VCAN exhibited high expression density in CFH^+^PEC subcluster C1 (*n* = 137 cells) (Figure 3(A–B)). Gene Ontology (GO) analysis revealed that this VCAN-enriched C1 subpopulation was functionally characterized by structural remodeling processes, including positive regulation of stress fiber assembly and actomyosin structure organization. Conversely, ESM1 showed predominant expression in ENDO subcluster C4 (*n* = 31 cells) (Figure 3(G–H)). Functionally, this ESM1-enriched endothelial subpopulation was closely associated with metabolic and stress responses, such as energy homeostasis and response to hypoxia. Pseudotime trajectory analysis of CFH+PEC cells revealed a branched developmental trajectory. Notably, VCAN expression displayed a transient upregulation pattern, peaking at early-to-mid pseudotime before declining (Figure 3(C,E)). This trajectory is consistent with the overall downregulation of VCAN observed in the DKD CFH+ PEC cluster (Figure 2(C)). This dynamic process was accompanied by distinct gene expression modules enriched in pathways such as muscle tissue development and apoptotic signaling (Figure 3(D)). Concurrently, the scores for fibrosis and inflammation signatures exhibited significant fluctuations along the CFH+PEC trajectory, ultimately showing a sharp increase at the late stage (Figure 3(F)). Similarly, ENDO cell pseudotime analysis suggested a complex branched trajectory, with ESM1 displaying a biphasic pattern (an initial increase followed by a continuous decline) (Figure 3(I,K)). The downregulation of ESM1 correlated with dynamic changes in pathways related to cell migration and adhesion (Figure 3(J)), alongside progressive alterations in fibrosis and inflammation scores (Figure 3(L)), suggesting a role in endothelial dysfunction during disease progression.

### 3.5. Alterations in intercellular communication support the dual-track injury mechanism

**Figure 4.**
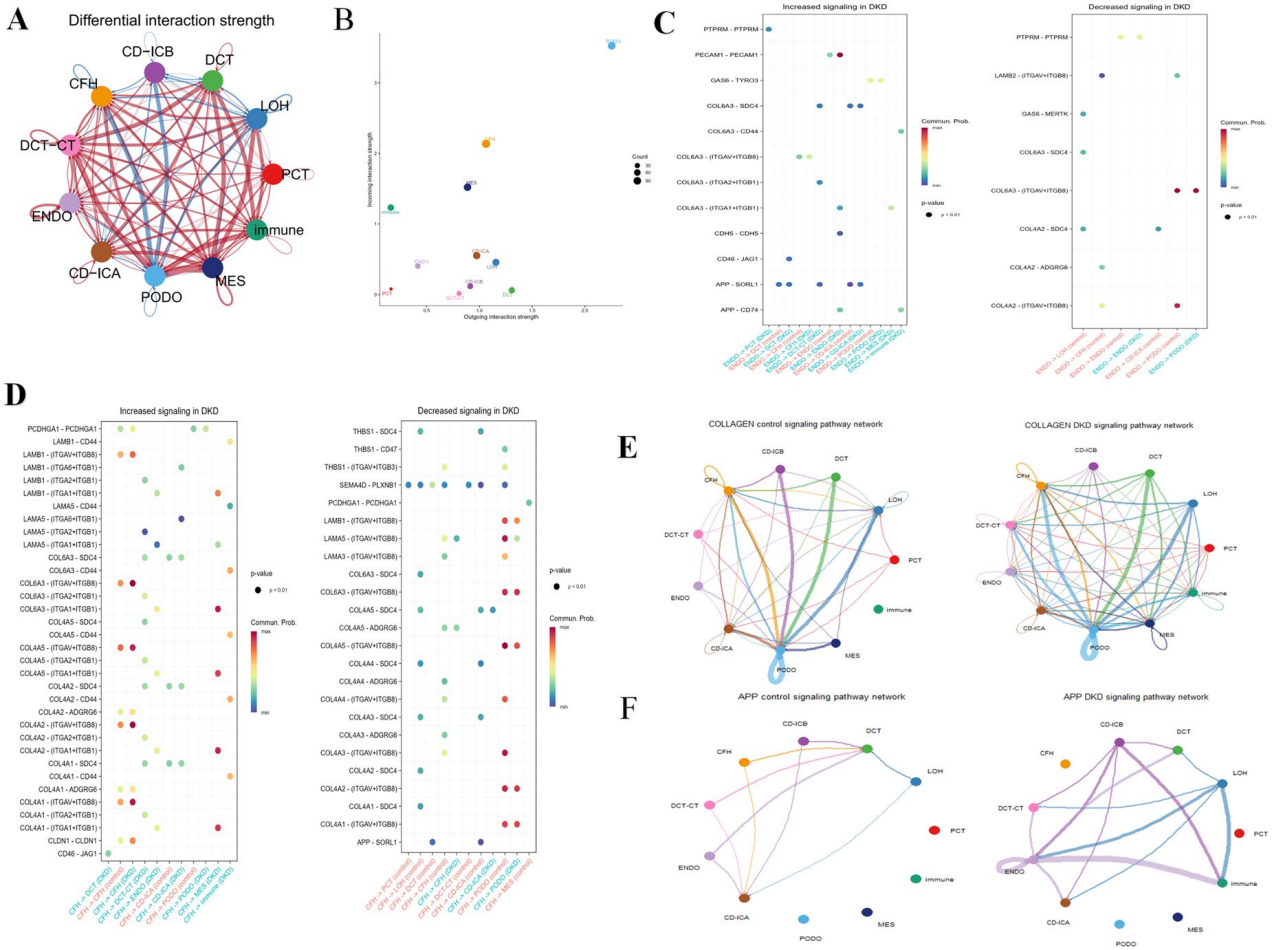
Inferring intercellular communication networks in DKD glomeruli. (A) Circle plots showing the number and strength of inferred interactions among cell types in Control and DKD groups. (B) Scatter plot identifying key sender and receiver populations based on dominant incoming and outgoing signaling patterns. (C–D) Bubble plots showing the up-regulated (left) and down-regulated (right) signaling ligand-receptor pairs in DKD. The x-axis includes both Control and DKD samples to provide a direct visual comparison of the communication probabilities (indicated by dot color) for the same ligand-receptor pair (y-axis) between the two groups. (E) Network plots of the Collagen (COL) signaling pathway, highlighting mesangial cells and CFH^+^ PECs as primary senders driving matrix remodeling. (F) Network plots of additional stress response pathways (e.g., APP).

Given the distinct cellular localization of VCAN and ESM1, we hypothesized that intercellular crosstalk orchestrates glomerular injury. CellChat analysis was performed to evaluate changes in ligand-receptor interactions. Comparison of the global communication networks revealed significant differences in interaction strength between the DKD and Control groups (Figure 4(A)). Scatter plot analysis identified the primary sender and receiver cell populations driving these changes (Figure 4(B)). Crucially, the analysis identified two major signaling axes that support our proposed VCAN/ESM1 ‘dual-track’ model. First, comparative bubble plot analysis (Figure 4(D)) directly demonstrated that specific enhanced Collagen ligand-receptor interactions, notably COL4A1-CD44 and COL4A1-SDC4, were primarily emitted by mesangial cells and CFH^+^ PECs (acting as the major senders) targeting other resident cells in DKD compared to Controls. Although VCAN is downregulated in CFH^+^ PECs, these cells maintain robust expression of distinct matrix molecules like COL4A1, allowing them to actively participate in paracrine collagen signaling. The structural alterations associated with matrix remodeling are further illustrated by the COLLAGEN signaling pathway network (Figure 4(E)). These findings align with mesangial VCAN upregulation and PEC-derived collagen signaling, suggesting that both cell types actively participate in paracrine-mediated fibrosis. Second, dysregulated endothelial communication provided a mechanistic basis for vascular-immune interactions. Endothelial cells exhibited altered outgoing signaling, characterized by increased interactions such as APP-CD74 targeting immune and resident populations (Figure 4(C)). The disruption of homeostatic endothelial signaling and the transition toward stress-associated pathways (e.g., the APP signaling network, Figure 4(F)) are consistent with ESM1 depletion, linking the loss of protective factors to the inflammatory microenvironment. Together, the DKD microenvironment is characterized by both matrix accumulation (VCAN-driven) and injury related to ESM1 depletion.

### 3.6. Multi-cohort validation and in vivo transcriptomic confirmation

**Figure 5.**
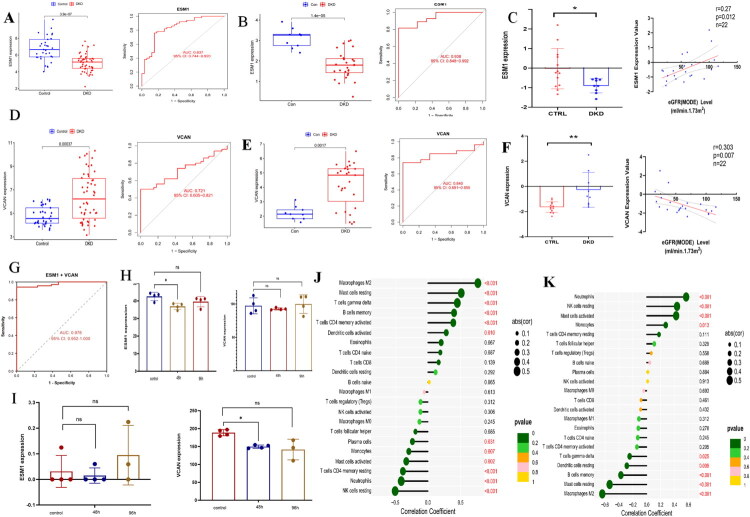
Validation of ESM1 and VCAN expression, diagnostic value, and clinical correlations. (A–B) Differential expression (boxplots) and diagnostic performance (ROC curves) of ESM1 in the training cohort (merged GSE30528 and GSE96804) (A) and the independent external validation cohort (GSE142025) (B). (C) Validation of ESM1 expression in the Nephroseq database and its correlation with eGFR. (D–E) Differential expression (boxplots) and ROC curves of VCAN in the training cohort (D) and the external validation cohort (E). (F) Validation of VCAN expression in the Nephroseq database and its correlation with eGFR. (G) Diagnostic performance (ROC curve) of the combined ESM1 and VCAN model. (H) Expression levels of ESM1 (left) and VCAN (right) in human glomerular endothelial cells co-cultured with podocytes under high glucose conditions for 48h and 96h (GSE220227). (I) Expression levels of ESM1 (left) and VCAN (right) in podocytes from the high-glucose co-culture model for 48h and 96h (GSE220228). (J) Correlation analysis between ESM1 expression and the relative abundance of 22 immune cell types. (K) Correlation analysis between VCAN expression and the relative abundance of 22 immune cell types. Data in (H) and (I) are presented as mean ± SD. *p < 0.05, **p < 0.01, ns: not significant. ROC: receiver operating characteristic; AUC: area under the curve; eGFR: estimated glomerular filtration rate.

To ensure the robustness of our findings, we evaluated these markers across multiple cohorts. In the training cohort, ESM1 was significantly downregulated in DKD (Figure 5(A), *p* < 0.001, AUC = 0.837), while VCAN was upregulated (Figure 5(D), *p* < 0.001, AUC = 0.721). These diagnostic patterns were successfully validated in an independent external cohort (GSE142025, comprising 27 DKD and 9 controls) (Figure 5(B,E)) and the Nephroseq database. Clinical correlation analysis revealed that ESM1 positively correlated with eGFR (Figure 5(C), *r* = 0.270, *p* = 0.012), suggesting a renoprotective association, whereas VCAN negatively correlated with eGFR (Figure 5(F), r=-0.303, *p* = 0.007). Notably, the combined ESM1-VCAN model showed excellent diagnostic value (AUC = 0.978, Figure 5(G)).

To validate the cell-type-associated expression and temporal dynamics of these biomarkers, we analyzed transcriptomic data from an *in vitro* high-glucose endothelial-podocyte co-culture model. In glomerular endothelial cells, ESM1 exhibited a significant acute downregulation at 48h followed by a non-significant compensatory trend at 96h (Figure 5(H)), While the *in vitro* acute phase captures a rapid protective depletion of ESM1, the subsequent compensatory trend at 96h mirrors the complex, multi-phasic state transitions inferred by our scRNA-seq pseudotime trajectory, albeit with distinct temporal dynamics. Strikingly, ESM1 expression was nearly undetectable in podocytes across all time points (Figure 5(I)), consistent with its endothelial-predominant expression. Conversely, podocyte VCAN expression significantly decreased at 48h (Figure 5(I)), suggesting that the observed VCAN upregulation in DKD bulk tissues is likely driven primarily by mesangial cells, rather than podocytes or PECs. Finally, immune infiltration analysis suggested that ESM1 positively correlated with anti-inflammatory M2 macrophages and resting mast cells (Figure 5(J)), while VCAN significantly correlated with neutrophils and resting NK cells (Figure 5(K)), suggesting VCAN may promote DKD progression through neutrophil recruitment.

### 3.7. In vivo validation and molecular docking prediction

**Figure 6.**
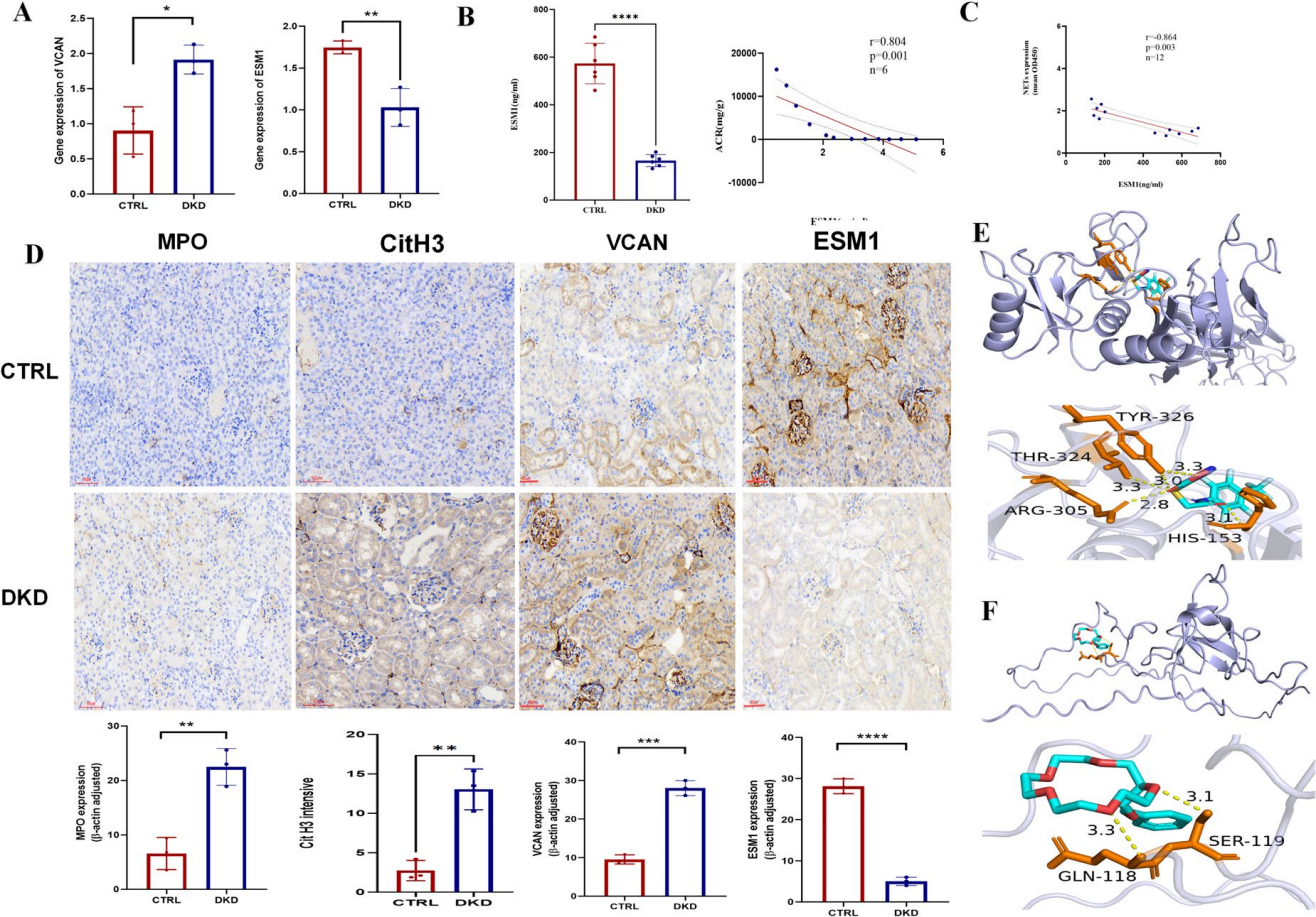
*In vivo* validation in db/db diabetic mice and molecular docking predictions. (A) qRT-PCR analysis of VCAN and ESM1 mRNA expression in kidney tissues from control (CTRL) and DKD mice. (B) Analysis of serum ESM1 protein levels (left) and the Pearson correlation analysis between serum ESM1 concentration and urinary albumin-to-creatinine ratio (ACR) (right). (C) Pearson correlation analysis between serum ESM1 concentration and NETs expression (mean OD450). (D) Representative immunohistochemistry (IHC) staining images and quantitative analysis of MPO, CitH3, VCAN, and ESM1 in kidney tissues from CTRL and DKD groups. Scale bar: 60 μm. (E) Molecular docking visualization showing the binding mode of ZINC005331 with the target protein, highlighting the 3D structure and key interacting residues (ARG-305, THR-324, TYR-326, HIS-153). (F) Molecular docking visualization showing the binding mode of ZINC171091 with the target protein, depicting hydrogen bond interactions with GLN-118 and SER-119. Data are presented as mean ± SEM. *p < 0.05, **p < 0.01, ***p < 0.001, ****p < 0.0001. qRT-PCR: quantitative real-time polymerase chain reaction; ACR: albumin-to-creatinine ratio; MPO: myeloperoxidase; CitH3: citrullinated histone H3.

To translate our bioinformatic findings into biological relevance, we performed experimental verification in db/db diabetic mice. qRT-PCR confirmed significant upregulation of VCAN mRNA and downregulation of ESM1 mRNA in DKD kidneys (Figure 6(A)). ELISA suggested diminished serum ESM1 levels in DKD mice, which inversely correlated with urinary ACR (*r* = −0.804, *p* = 0.001), linking reduced circulating ESM1 to proteinuria progression (Figure 6(B)). Notably, a significant negative correlation was observed between serum ESM1 concentration and NETs expression (*r* = −0.864, *p* = 0.003) (Figure 6(C)). IHC verified these changes at the protein level: MPO, CitH3 (NET markers), and VCAN were elevated in DKD kidneys, with VCAN deposition prominent in the Bowman’s capsule region. Conversely, ESM1 protein in glomerular endothelial areas was reduced (Figure 6(D)). Finally, to explore the therapeutic potential of targeting these core genes, we utilized the GraphBAN model. ZINC005331 was identified as a potential VCAN inhibitor (binding affinity −9.6 kcal/mol), and ZINC171091 as a potential ESM1 modulator (binding affinity −6.6 kcal/mol) (Figure 6(E–F)). We emphasize that these molecular docking results are purely predictive and lack experimental validation at this stage.

## 4. Discussion

DKD is increasingly recognized as a multifactorial disorder involving metabolic stress, endothelial dysfunction, immune activation, and progressive extracellular matrix remodeling. In this context, the present study integrated bulk transcriptomics, single-cell RNA sequencing, and experimental validation to identify VCAN and ESM1 as cell-type-associated molecular signatures linked to NET-related glomerular injury in DKD. Compared with analyses based solely on differential gene expression, this integrative strategy allowed these candidates to be interpreted within a broader biological context connecting inflammatory activation, microvascular injury, and tissue remodeling.

### 4.1. VCAN and indirect paracrine-associated fibrotic responses

Our single-cell analysis suggested that VCAN may be involved in indirect NET-associated inflammatory and matrix-remodeling responses. VCAN was highly expressed in both CFH+ PECs and mesangial cells, though exhibiting divergent expression trends in DKD (upregulated in mesangial cells and downregulated in PECs). This complex pattern, alongside the pseudotime trajectory showing a late-stage decline in PECs, suggests that VCAN dynamics in these compartments may reflect a secondary, stage-dependent response within a NET-enriched glomerular microenvironment rather than direct NET-associated signaling in these cells. In this proposed framework, endothelial injury may promote the release of inflammatory or stress-related mediators that influence adjacent mesangial cells and PECs through paracrine signaling, thereby contributing to extracellular matrix remodeling [4,23,24]. Consistent with this interpretation, our inferred intercellular communication analysis indicated enhanced collagen-centered signaling originating from mesangial cells and CFH^+^ PECs, including representative interactions such as COL4A1–CD44, COL4A1–SDC4, and collagen–integrin receptor pairs. These predicted interactions are compatible with fibrotic signaling cascades in which collagen-related pathways are linked to persistent matrix activation [25]. Accordingly, VCAN may represent a component of an activated matrix-remodeling network rather than a passive bystander. The diabetic milieu may further amplify this process, as AGE accumulation and oxidative stress can increase the susceptibility of glomerular cells to inflammatory stimulation [26]. In addition, pseudotime-based trajectory analysis showed increasing VCAN expression along inferred cell-state transitions, together with associations with matrix remodeling and apoptosis-related programs. Its positive correlation with neutrophil infiltration abundance further supports a potential association between VCAN and inflammatory recruitment in DKD, although the directionality of this relationship remains to be clarified.

### 4.2. ESM1 depletion and endothelial injury in a NET-enriched context

In contrast to VCAN, ESM1 displayed a distinct pattern characterized by consistent downregulation, supporting its relevance as an indicator of endothelial dysfunction. ESM1 was decreased across multiple datasets, including single-cell analysis of early-stage DKD, bulk tissue transcriptomic profiling, and *in vivo* db/db models. This reduction was negatively associated with disease severity, suggesting that ESM1 loss may represent an early and persistent feature of DKD progression. Given the established role of ESM1 in glycocalyx maintenance and endothelial barrier integrity [27,28], its downregulation is consistent with impairment of these protective mechanisms.

Our ligand-receptor analysis further suggested disruption of endothelial-associated communication, including altered PECAM1-, PTPRM-, GAS6-, and APP-related signaling, which is in line with the loss of vascular homeostasis and increased endothelial stress in DKD [29]. Thus, reduced ESM1 expression may serve as a molecular readout of endothelial injury within an inflammatory microenvironment. Several observations support this interpretation. ESM1 co-localized with the endothelial marker EGFL7, while GSEA indicated preferential enrichment of NET-associated injury signals in endothelial cells. Pathway analysis also showed upregulation of genes related to tight junction disturbance, fluid shear stress, and MAPK/TNF signaling in DKD endothelial cells, suggesting that ESM1 loss occurs in parallel with barrier dysfunction and inflammatory activation [30]. The decrease of ESM1 in bulk tissue may reflect a combination of reduced healthy endothelial cell abundance, transcriptional repression in injured endothelial cells, and possible degradation by proteases released in NET-rich inflammatory settings [31,32]. In contrast to the pro-inflammatory associations observed for VCAN, ESM1 showed positive correlations with M2 macrophages, mast cells, and eGFR, further supporting its potential renoprotective relevance.

### 4.3. A proposed dual-track model of glomerular injury in DKD

Taken together, these findings support a cell-type-associated pattern of NET-related signaling and glomerular injury in DKD. GSEA revealed an important distinction within the diabetic glomerular inflammatory landscape: although both endothelial cells and CFH^+^ PECs showed generalized inflammatory activation, NET-associated gene signatures were preferentially enriched in endothelial cells. Given their fenestrated architecture and direct exposure to the circulation, glomerular endothelial cells may be particularly vulnerable to circulating NET components, including extracellular DNA, histones, and MPO, which is consistent with the increased MPO deposition observed in db/db glomeruli. In combination with diabetic hemodynamic alterations and metabolic stress, these observations support a proposed framework in which metabolic, hemodynamic, and NET-related factors may converge to aggravate glomerular injury [33]. By contrast, the divergent VCAN dynamics across resident cells-upregulation in mesangial cells and a stage-dependent decline in CFH^+^ PECs-appears more consistent with indirect paracrine activation within the injured glomerular microenvironment. The VCAN-CFH co-expression pattern also raises the possibility that complement-related activity and matrix remodeling may interact to promote glomerulosclerotic progression [34]. Importantly, our intercellular communication network reconstruction suggested a polarized glomerular microenvironment characterized by a VCAN-associated matrix-remodeling axis and an ESM1-depleted endothelial injury axis. On this basis, we propose a ‘dual-track’ model in which endothelial injury accompanied by ESM1 loss may occur in parallel with paracrine activation of PECs (associated with collagen deposition) and mesangial cells (associated with VCAN upregulation), collectively contributing to matrix remodeling. This model underscores the potential importance of intercellular crosstalk in DKD progression, while requiring further functional validation.

### 4.4. Translational relevance and limitations

From a translational perspective, both markers showed moderate-to-good diagnostic performance in ROC analyses. In addition, our GraphBAN-based analysis identified ZINC005331 and ZINC171091 as computationally predicted candidate compounds potentially associated with VCAN and ESM1, respectively. However, these findings are purely predictive and exploratory. Nevertheless, several limitations should be acknowledged. First, the scRNA-seq dataset included a limited number of early-stage DKD samples, and our conclusions regarding pseudotime dynamics and cell-cell communication should therefore be interpreted cautiously. These findings need to be validated in larger cohorts or late-stage DKD datasets to fully capture the disease progression. Importantly, pseudotime analysis infers potential cellular state transitions rather than true biological time. Future studies incorporating functional time-course *in vivo* experiments across different disease stages are required to definitively validate these true time-dependent dynamic changes. In addition, the statistical significance of formal NET gene-set enrichment was sensitive to the gene-set definition used: a DKD-contextualized NRG set and a canonical NET CoreGene Score were significantly associated with DKD, whereas standardized NET-formation gene sets were not. The convergent cellular localization observed across multiple gene-set definitions nevertheless indicates that our central conclusions do not depend on any single gene-set choice; accordingly, we have framed the NET-related findings as associative rather than mechanistic. Second, the proposed dual-track injury model is mainly derived from correlative evidence generated by multi-omics analyses; although our *in vivo* experiments confirmed the overall expression trends of these targets, we were unable to perform *in vivo* co-immunofluorescence to definitively confirm their cell-specific spatial localization at the protein level due to the lack of compatible antibodies for double-staining. Therefore, future functional experiments—specifically, the knockdown or overexpression of VCAN and ESM1 in relevant cell types under NET-rich conditions—are needed to determine causality more directly and fully support the proposed model. Third, as our drug screening lacks experimental validation, the candidate compounds identified by molecular docking must be regarded strictly as in silico predictions, requiring rigorous *in vitro* and *in vivo* pharmacological validation before any therapeutic claims can be made. Fourth, although serum ESM1 showed strong correlations with clinical parameters, future studies should also assess urinary ESM1 as a potentially more accessible noninvasive biomarker. Finally, prospective cohorts with more comprehensive clinical metadata are needed to better define the diagnostic and prognostic value of VCAN and ESM1.

## 5. Conclusions

In summary, this integrative multi-omics study identified VCAN and ESM1 as promising cell-type-associated candidate biomarkers linked to NET-related glomerular injury in diabetic kidney disease. VCAN was primarily associated with mesangial and matrix-remodeling-related cell populations, whereas ESM1 was predominantly associated with glomerular endothelial cells, highlighting complementary aspects of inflammatory remodeling and microvascular dysfunction. These findings provide biologically meaningful insight into the molecular heterogeneity of DKD, support a proposed dual-track model of glomerular injury, and suggest the potential value of VCAN and ESM1 for disease stratification and future therapeutic investigation.

## Supplementary Material

Supplementary_Material.docx

## Data Availability

The data that support the findings of this study are openly available in the Gene Expression Omnibus (GEO) at https://www.ncbi.nlm.nih.gov/geo/, reference numbers GSE30528, GSE96804, GSE220227, GSE220228, GSE131882,GSE142025, and GSE189875. Additional data supporting the findings of this study are available from the corresponding author upon reasonable request. Additional Supplementary Material is available in the ScienceDB repository at: https://www.scidb.cn/anonymous/M0VGWjdu.
