# Supplementary material for: Integrative multi-omics analysis identifies VCAN and ESM1 as candidate cell-type-associated biomarkers of NET-related glomerular injury in diabetic kidney disease
Source: Ren Fail. 2026 Aug 6;48(1):2707003. doi: 10.1080/0886022X.2026.2707003 (PMC13455701; doi:10.1080/0886022X.2026.2707003)
Supplement: Supplementary file 1 [file IRNF_A_2707003_SM9749.docx]

Supplementary Material

# 1 Supplementary Figures and Tables

## 1.1 Supplementary Figure S1

`

**Supplementary Figure S1. Batch effect correction and single-cell quality control.**(A–B) Principal component analysis (PCA) plots of bulk RNA-seq datasets (GSE30528 and GSE96804) showing sample distribution after (A) and before (B) batch correction.(C–D) UMAP projection of scRNA-seq data identifying 15 unsupervised clusters (C) and corresponding cell type annotations (D).(E) Violin plots showing distribution of detected genes (nFeature_RNA), mitochondrial counts (percent.mt), ribosomal counts (percent.ribo), and hemoglobin counts (percent.hb) in control and DKD samples (n=3/group).

## 1.2 Supplementary Figure S2

**Supplementary Figure S2. Robustness and sensitivity analyses of NET-related gene sets and cell-type-associated activity scores.** (A) Violin plot showing the distribution of the NET CoreGene Score (derived from ten canonical NET genes) in bulk glomerular tissues from Control and DKD groups. Statistical significance was determined using the Wilcoxon rank-sum test （P<0.001).(B) Boxplot illustrating the distribution of the custom NETs score (AUCell) across 10 major renal cell types in Control and DKD groups.(C-F) UMAP density plots demonstrating the distribution and density of AUCell scores across all 11 renal cell clusters based on different gene-set definitions:(C) GO_NETs (Gene Ontology NET-formation gene set),(D) KEGG_NETs (KEGG NET-formation pathway),(E) WP_NETs (WikiPathways NET-formation pathway), and(F) NRGs_DKD (the disease-contextualized NET-related gene set).(G) Glomerular-focused UMAP density plot showing the distribution of the disease-contextualized NRGs_DKD score specifically within glomerular-resident cell subpopulations (including ENDO, PODO, MES, CFH^+^ PECs, and immune cells), highlighting concentrated activity within the glomerular filtration barrier (endothelial cells and podocytes).

## 1.3 Supplementary Figure 3

**Supplementary Figure S3. Transcriptomic profiles, functional enrichment of cell subclusters, and validation of candidate markers.**(A, B) Heatmaps of gene expression modules and enriched GO terms across single-cell subclusters of CFH^+^ parietal epithelial cells (PECs) (A) and endothelial cells (ENDO) (B).(C, D) mRNA expression of VCAN (C) and ESM1 (D) in endothelial cells treated with or without NETs for 12 h (GSE189875, n = 5/group). Data represent mean ± SD; statistical significance was assessed by unpaired t-test (ns, not significant).(E) Dotplot showing the expression levels and percentages of canonical NET-responsive genes and receptors across renal cell populations in control and DKD groups.

## **2.Supplementary Tables**

### 2.1 Table S1: List of differentially expressed genes (DEGs).

| **id** | **logFC** | **AveExpr** | **t** | **P.Value** | **adj.P.Val** | **B** |
| --- | --- | --- | --- | --- | --- | --- |
| DUSP1 | -1.4200 | 5.4008 | -11.4563 | 0.0000 | 0.0000 | 32.1626 |
| CXCR2 | -1.2784 | 3.7909 | -9.2598 | 0.0000 | 0.0000 | 22.4985 |
| FOS | -2.6492 | 4.5464 | -8.9972 | 0.0000 | 0.0000 | 21.3262 |
| G6PC | -3.1710 | 6.5097 | -8.7807 | 0.0000 | 0.0000 | 20.3593 |
| ZFP36 | -2.0913 | 7.5191 | -8.5108 | 0.0000 | 0.0000 | 19.1540 |
| CDH10 | -1.5688 | 3.4381 | -8.3377 | 0.0000 | 0.0000 | 18.3821 |
| WT1-AS | -1.0190 | 4.9032 | -8.1536 | 0.0000 | 0.0000 | 17.5621 |
| LPL | -1.4923 | 4.8031 | -8.0889 | 0.0000 | 0.0000 | 17.2741 |
| TPPP3 | -1.5910 | 6.7247 | -8.0698 | 0.0000 | 0.0000 | 17.1894 |
| CXCR1 | -1.1942 | 3.4445 | -8.0169 | 0.0000 | 0.0000 | 16.9543 |
| LOX | -1.5390 | 7.2629 | -8.0082 | 0.0000 | 0.0000 | 16.9153 |
| PTGDS | -1.2716 | 7.7583 | -7.9765 | 0.0000 | 0.0000 | 16.7746 |
| PDK4 | -1.8399 | 7.2517 | -7.6708 | 0.0000 | 0.0000 | 15.4204 |
| PRKAR2B | -1.3045 | 6.7679 | -7.4825 | 0.0000 | 0.0000 | 14.5900 |
| DPP6 | -1.4114 | 6.3543 | -7.4154 | 0.0000 | 0.0000 | 14.2949 |
| HIST1H2BD | -1.0822 | 5.0724 | -7.2363 | 0.0000 | 0.0000 | 13.5097 |
| FN1 | 2.8503 | 6.8109 | 7.1912 | 0.0000 | 0.0000 | 13.3131 |
| ARHGAP19 | -1.1819 | 7.5007 | -7.0201 | 0.0000 | 0.0000 | 12.5676 |
| CA10 | -1.1150 | 5.2500 | -6.9940 | 0.0000 | 0.0000 | 12.4543 |
| LRRC2 | -1.4354 | 7.0150 | -6.7818 | 0.0000 | 0.0000 | 11.5370 |
| NPHS1 | -1.5215 | 8.3942 | -6.7455 | 0.0000 | 0.0000 | 11.3810 |
| G0S2 | -1.1267 | 6.2499 | -6.7301 | 0.0000 | 0.0000 | 11.3147 |
| S100A8 | -1.4410 | 4.4165 | -6.7183 | 0.0000 | 0.0000 | 11.2641 |
| HSD17B14 | -1.1203 | 4.5915 | -6.6453 | 0.0000 | 0.0000 | 10.9510 |
| LUM | 2.5846 | 6.1444 | 6.6379 | 0.0000 | 0.0000 | 10.9196 |
| JUNB | -1.4172 | 5.3657 | -6.5115 | 0.0000 | 0.0000 | 10.3802 |
| PCOLCE2 | -1.2000 | 8.7344 | -6.5060 | 0.0000 | 0.0000 | 10.3570 |
| TGFBI | 1.1313 | 4.2641 | 6.4627 | 0.0000 | 0.0000 | 10.1728 |
| S100A12 | -1.9318 | 4.1870 | -6.4202 | 0.0000 | 0.0000 | 9.9926 |
| COL1A2 | 1.4858 | 4.0924 | 6.3022 | 0.0000 | 0.0000 | 9.4945 |
| S100A9 | -1.9301 | 7.2608 | -6.2981 | 0.0000 | 0.0000 | 9.4769 |
| ESM1 | -1.7096 | 5.7471 | -6.2898 | 0.0000 | 0.0000 | 9.4421 |
| PTGS2 | -1.6190 | 3.9985 | -6.1916 | 0.0000 | 0.0000 | 9.0304 |
| DACH1 | -1.1561 | 10.0985 | -6.1709 | 0.0000 | 0.0000 | 8.9436 |
| TCF21 | -1.1738 | 10.6229 | -6.1681 | 0.0000 | 0.0000 | 8.9318 |
| RGS2 | -1.4696 | 7.3710 | -6.1261 | 0.0000 | 0.0000 | 8.7567 |
| TNNI1 | -1.3857 | 7.8242 | -6.1196 | 0.0000 | 0.0000 | 8.7298 |
| CYP27B1 | -1.1104 | 3.2936 | -6.1178 | 0.0000 | 0.0000 | 8.7221 |
| MOXD1 | 1.8149 | 4.4265 | 6.0975 | 0.0000 | 0.0000 | 8.6377 |
| FOSB | -1.0809 | 2.9005 | -6.0172 | 0.0000 | 0.0000 | 8.3045 |
| FGF9 | -1.0009 | 5.5990 | -6.0006 | 0.0000 | 0.0000 | 8.2356 |
| MMP2 | 1.0588 | 4.3890 | 5.9966 | 0.0000 | 0.0000 | 8.2190 |
| FPR1 | -1.1032 | 4.3213 | -5.9530 | 0.0000 | 0.0000 | 8.0391 |
| COL6A3 | 1.3899 | 3.7051 | 5.9173 | 0.0000 | 0.0000 | 7.8921 |
| EGF | -1.2628 | 4.0348 | -5.9012 | 0.0000 | 0.0000 | 7.8258 |
| ALB | -1.9526 | 5.4954 | -5.8582 | 0.0000 | 0.0000 | 7.6497 |
| DDN | -1.0066 | 4.7785 | -5.8039 | 0.0000 | 0.0000 | 7.4275 |
| THBS2 | 1.1669 | 3.7293 | 5.8021 | 0.0000 | 0.0000 | 7.4203 |
| FCGR3B | -1.0607 | 5.0322 | -5.7879 | 0.0000 | 0.0000 | 7.3623 |
| HRSP12 | -1.3069 | 7.5134 | -5.7515 | 0.0000 | 0.0000 | 7.2139 |
| MARCKS | 1.3550 | 7.2166 | 5.7292 | 0.0000 | 0.0000 | 7.1233 |
| EHD3 | -1.4599 | 8.3228 | -5.6173 | 0.0000 | 0.0000 | 6.6714 |
| ADH1B | 1.4936 | 4.5517 | 5.6000 | 0.0000 | 0.0000 | 6.6015 |
| EGR1 | -1.8377 | 7.2286 | -5.5666 | 0.0000 | 0.0000 | 6.4677 |
| ASPN | 1.1910 | 4.5734 | 5.5195 | 0.0000 | 0.0000 | 6.2791 |
| DCXR | -1.2152 | 8.6669 | -5.3437 | 0.0000 | 0.0000 | 5.5822 |
| VSIG4 | 1.2333 | 3.9589 | 5.3127 | 0.0000 | 0.0000 | 5.4608 |
| APOD | -1.0675 | 11.8842 | -5.2793 | 0.0000 | 0.0000 | 5.3299 |
| MAGI2 | -1.0679 | 6.6794 | -5.2737 | 0.0000 | 0.0000 | 5.3078 |
| ATF3 | -1.0544 | 4.0237 | -5.2137 | 0.0000 | 0.0000 | 5.0740 |
| TNNT2 | -1.1171 | 5.9867 | -5.2055 | 0.0000 | 0.0000 | 5.0423 |
| C7 | 1.8947 | 7.6109 | 5.2030 | 0.0000 | 0.0000 | 5.0324 |
| FBP1 | -1.2153 | 5.7203 | -5.2012 | 0.0000 | 0.0000 | 5.0255 |
| MS4A6A | 1.3736 | 4.6547 | 5.1844 | 0.0000 | 0.0000 | 4.9605 |
| CR1 | -1.1518 | 8.4803 | -5.1334 | 0.0000 | 0.0000 | 4.7632 |
| HPGD | -1.3210 | 6.2871 | -5.1232 | 0.0000 | 0.0000 | 4.7239 |
| MFAP4 | 1.0300 | 5.4341 | 5.1142 | 0.0000 | 0.0000 | 4.6893 |
| AEBP1 | 1.0109 | 5.1903 | 5.0908 | 0.0000 | 0.0000 | 4.5992 |
| APOH | -1.1775 | 3.9475 | -5.0704 | 0.0000 | 0.0000 | 4.5212 |
| TNC | 1.1082 | 5.5740 | 5.0369 | 0.0000 | 0.0000 | 4.3927 |
| CYP2B6 | -1.0078 | 4.3978 | -4.9966 | 0.0000 | 0.0000 | 4.2395 |
| CPA3 | 1.1916 | 3.2724 | 4.9947 | 0.0000 | 0.0000 | 4.2322 |
| CCL21 | 1.8332 | 5.2906 | 4.9888 | 0.0000 | 0.0000 | 4.2098 |
| CHI3L1 | -1.3133 | 8.9941 | -4.9820 | 0.0000 | 0.0000 | 4.1839 |
| C3 | 1.9198 | 4.8639 | 4.8279 | 0.0000 | 0.0001 | 3.6039 |
| PTN | 1.1348 | 5.3706 | 4.8252 | 0.0000 | 0.0001 | 3.5937 |
| SERPINF1 | 1.1814 | 4.0429 | 4.8178 | 0.0000 | 0.0001 | 3.5663 |
| FBLN5 | 1.2437 | 6.2716 | 4.7970 | 0.0000 | 0.0001 | 3.4887 |
| PCK1 | -1.1887 | 5.6531 | -4.7753 | 0.0000 | 0.0001 | 3.4080 |
| FMO3 | 1.0547 | 4.3432 | 4.7182 | 0.0000 | 0.0001 | 3.1972 |
| MMP7 | 2.1080 | 7.7451 | 4.7147 | 0.0000 | 0.0001 | 3.1842 |
| GSTA1 | -1.5366 | 9.7396 | -4.6309 | 0.0000 | 0.0001 | 2.8772 |
| NR4A2 | -1.1448 | 3.7805 | -4.6292 | 0.0000 | 0.0001 | 2.8711 |
| CDH6 | 1.2552 | 6.1323 | 4.5841 | 0.0000 | 0.0002 | 2.7072 |
| SLC12A3 | -1.4204 | 5.1776 | -4.5693 | 0.0000 | 0.0002 | 2.6537 |
| CCL19 | 1.5914 | 4.9513 | 4.5599 | 0.0000 | 0.0002 | 2.6196 |
| HPD | -1.7403 | 6.8469 | -4.5471 | 0.0000 | 0.0002 | 2.5733 |
| FABP1 | -1.1589 | 6.6103 | -4.5322 | 0.0000 | 0.0002 | 2.5199 |
| CCL2 | 1.3007 | 9.7974 | 4.5246 | 0.0000 | 0.0002 | 2.4926 |
| PRODH2 | -1.0713 | 5.7001 | -4.4987 | 0.0000 | 0.0002 | 2.3993 |
| AFM | -1.4189 | 5.8567 | -4.4749 | 0.0000 | 0.0002 | 2.3142 |
| AGMAT | -1.4686 | 7.0572 | -4.4291 | 0.0000 | 0.0003 | 2.1512 |
| FPR3 | 1.1535 | 4.0320 | 4.4014 | 0.0000 | 0.0003 | 2.0531 |
| VCAN | 1.6001 | 5.7693 | 4.3499 | 0.0000 | 0.0003 | 1.8717 |
| SST | -1.2270 | 5.1762 | -4.0939 | 0.0001 | 0.0007 | 0.9903 |
| HAO2 | -1.4431 | 6.2257 | -4.0480 | 0.0001 | 0.0008 | 0.8359 |
| ALDOB | -1.2295 | 12.0573 | -4.0193 | 0.0001 | 0.0009 | 0.7400 |
| GLYAT | -1.3500 | 8.0943 | -4.0156 | 0.0001 | 0.0009 | 0.7276 |
| IGJ | 1.6617 | 5.4630 | 3.9421 | 0.0002 | 0.0011 | 0.4844 |
| HBB | -1.3818 | 7.8129 | -3.9272 | 0.0002 | 0.0011 | 0.4352 |
| BHMT | -1.1204 | 8.5211 | -3.8185 | 0.0003 | 0.0015 | 0.0819 |
| B3GALT2 | -1.1196 | 6.3244 | -3.7001 | 0.0004 | 0.0021 | -0.2949 |
| IGKC | 1.0496 | 4.5323 | 3.6679 | 0.0004 | 0.0023 | -0.3962 |
| CALB1 | -1.0038 | 5.2163 | -3.6399 | 0.0005 | 0.0024 | -0.4835 |
| CCL18 | 1.3020 | 3.3772 | 3.6180 | 0.0005 | 0.0026 | -0.5515 |
| KRT19 | 1.0042 | 4.9677 | 3.5785 | 0.0006 | 0.0029 | -0.6733 |
| MRC1 | 1.0030 | 4.8448 | 3.5722 | 0.0006 | 0.0029 | -0.6927 |
| KNG1 | -1.2511 | 7.7790 | -3.4918 | 0.0008 | 0.0036 | -0.9375 |
| APOM | -1.0580 | 7.2433 | -3.4853 | 0.0008 | 0.0037 | -0.9571 |
| UMOD | -1.4735 | 7.2216 | -3.4833 | 0.0008 | 0.0037 | -0.9632 |
| IGF1 | -1.3379 | 6.9694 | -3.4832 | 0.0008 | 0.0037 | -0.9634 |
| RNASE6 | 1.1373 | 5.5911 | 3.4484 | 0.0009 | 0.0041 | -1.0681 |
| APOC1 | 1.2044 | 5.6358 | 3.2749 | 0.0015 | 0.0063 | -1.5768 |
| PLCG2 | -1.0030 | 5.8122 | -3.1204 | 0.0025 | 0.0092 | -2.0130 |
| CYP4A11 | -1.0051 | 6.6115 | -2.7878 | 0.0066 | 0.0202 | -2.8949 |

### **2.2 Table S2: GO enrichment analysis results.**

| **ONTOLOGY** | **ID** | **Description** | **GeneRatio** | **BgRatio** | **pvalue** | **p.adjust** | **qvalue** | **geneID** | **Count** |
| --- | --- | --- | --- | --- | --- | --- | --- | --- | --- |
| BP | GO:1990266 | neutrophil migration | 10/111 | 129/18888 | 4.46E-09 | 1.08E-05 | 8.01E-06 | S100A12/S100A9/UMOD/S100A8/CXCR2/CXCR1/CCL2/CCL18/CCL19/CCL21 | 10 |
| BP | GO:0030593 | neutrophil chemotaxis | 9/111 | 107/18888 | 1.34E-08 | 1.08E-05 | 8.01E-06 | S100A12/S100A9/S100A8/CXCR2/CXCR1/CCL2/CCL18/CCL19/CCL21 | 9 |
| BP | GO:0030595 | leukocyte chemotaxis | 12/111 | 237/18888 | 1.52E-08 | 1.08E-05 | 8.01E-06 | S100A12/S100A9/S100A8/DUSP1/CXCR2/CXCR1/MMP2/PTN/CCL2/CCL18/CCL19/CCL21 | 12 |
| BP | GO:0097529 | myeloid leukocyte migration | 12/111 | 239/18888 | 1.67E-08 | 1.08E-05 | 8.01E-06 | S100A12/S100A9/UMOD/S100A8/DUSP1/CXCR2/CXCR1/MMP2/CCL2/CCL18/CCL19/CCL21 | 12 |
| BP | GO:0097530 | granulocyte migration | 10/111 | 154/18888 | 2.47E-08 | 1.27E-05 | 9.47E-06 | S100A12/S100A9/UMOD/S100A8/CXCR2/CXCR1/CCL2/CCL18/CCL19/CCL21 | 10 |
| BP | GO:0060326 | cell chemotaxis | 13/111 | 322/18888 | 5.42E-08 | 2.33E-05 | 1.73E-05 | S100A12/S100A9/LOX/S100A8/DUSP1/CXCR2/CXCR1/MMP2/PTN/CCL2/CCL18/CCL19/CCL21 | 13 |
| BP | GO:0071621 | granulocyte chemotaxis | 9/111 | 128/18888 | 6.41E-08 | 2.35E-05 | 1.75E-05 | S100A12/S100A9/S100A8/CXCR2/CXCR1/CCL2/CCL18/CCL19/CCL21 | 9 |
| BP | GO:0050900 | leukocyte migration | 14/111 | 396/18888 | 8.38E-08 | 2.35E-05 | 1.75E-05 | S100A12/S100A9/UMOD/S100A8/DUSP1/CXCR2/CXCR1/APOD/MMP2/PTN/CCL2/CCL18/CCL19/CCL21 | 14 |
| BP | GO:0006935 | chemotaxis | 15/111 | 466/18888 | 9.49E-08 | 2.35E-05 | 1.75E-05 | S100A12/S100A9/LOX/S100A8/DUSP1/CXCR2/CXCR1/FPR1/MMP2/PTN/FPR3/CCL2/CCL18/CCL19/CCL21 | 15 |
| BP | GO:0006631 | fatty acid metabolic process | 14/111 | 401/18888 | 9.78E-08 | 2.35E-05 | 1.75E-05 | PDK4/PTGS2/GSTA1/LPL/HAO2/HPGD/PRKAR2B/PTGDS/PCK1/FABP1/CYP2B6/CYP4A11/APOC1/C3 | 14 |
| BP | GO:0042330 | taxis | 15/111 | 468/18888 | 1.00E-07 | 2.35E-05 | 1.75E-05 | S100A12/S100A9/LOX/S100A8/DUSP1/CXCR2/CXCR1/FPR1/MMP2/PTN/FPR3/CCL2/CCL18/CCL19/CCL21 | 15 |
| BP | GO:0098869 | cellular oxidant detoxification | 8/111 | 99/18888 | 1.21E-07 | 2.43E-05 | 1.81E-05 | ALB/S100A9/PTGS2/GSTA1/HBB/FABP1/APOM/FBLN5 | 8 |
| BP | GO:0007565 | female pregnancy | 10/111 | 183/18888 | 1.26E-07 | 2.43E-05 | 1.81E-05 | FOS/PTGS2/TPPP3/RGS2/JUNB/HPGD/CYP27B1/FOSB/MMP2/PTN | 10 |
| BP | GO:1990868 | response to chemokine | 8/111 | 101/18888 | 1.42E-07 | 2.43E-05 | 1.81E-05 | LOX/DUSP1/CXCR2/CXCR1/CCL2/CCL18/CCL19/CCL21 | 8 |
| BP | GO:1990869 | cellular response to chemokine | 8/111 | 101/18888 | 1.42E-07 | 2.43E-05 | 1.81E-05 | LOX/DUSP1/CXCR2/CXCR1/CCL2/CCL18/CCL19/CCL21 | 8 |
| BP | GO:0046697 | decidualization | 5/111 | 25/18888 | 3.10E-07 | 4.99E-05 | 3.72E-05 | PTGS2/TPPP3/JUNB/CYP27B1/PTN | 5 |
| BP | GO:0044703 | multi-organism reproductive process | 10/111 | 204/18888 | 3.46E-07 | 5.25E-05 | 3.91E-05 | FOS/PTGS2/TPPP3/RGS2/JUNB/HPGD/CYP27B1/FOSB/MMP2/PTN | 10 |
| BP | GO:0097305 | response to alcohol | 11/111 | 259/18888 | 3.68E-07 | 5.27E-05 | 3.93E-05 | FOS/RGS2/S100A8/SLC12A3/HPGD/FBP1/PCK1/FOSB/TNC/CCL19/CCL21 | 11 |
| BP | GO:0044706 | multi-multicellular organism process | 10/111 | 212/18888 | 4.93E-07 | 6.49E-05 | 4.83E-05 | FOS/PTGS2/TPPP3/RGS2/JUNB/HPGD/CYP27B1/FOSB/MMP2/PTN | 10 |
| BP | GO:1990748 | cellular detoxification | 8/111 | 119/18888 | 5.03E-07 | 6.49E-05 | 4.83E-05 | ALB/S100A9/PTGS2/GSTA1/HBB/FABP1/APOM/FBLN5 | 8 |
| BP | GO:0048260 | positive regulation of receptor-mediated endocytosis | 6/111 | 53/18888 | 6.59E-07 | 8.09E-05 | 6.03E-05 | EGF/MAGI2/PLCG2/CCL19/CCL21/C3 | 6 |
| BP | GO:0097237 | cellular response to toxic substance | 8/111 | 129/18888 | 9.32E-07 | 0.000109175 | 8.13E-05 | ALB/S100A9/PTGS2/GSTA1/HBB/FABP1/APOM/FBLN5 | 8 |
| BP | GO:0032496 | response to lipopolysaccharide | 12/111 | 348/18888 | 9.74E-07 | 0.000109175 | 8.13E-05 | FOS/ZFP36/S100A9/PTGS2/UMOD/S100A8/HPGD/PCK1/CYP27B1/PLCG2/MRC1/CCL2 | 12 |
| BP | GO:0031667 | response to nutrient levels | 14/111 | 490/18888 | 1.09E-06 | 0.000117621 | 8.76E-05 | FOS/ZFP36/ALB/PDK4/PTGS2/LPL/SLC12A3/SST/FBP1/PCK1/FABP1/CYP27B1/ATF3/TNC | 14 |
| BP | GO:0060135 | maternal process involved in female pregnancy | 6/111 | 59/18888 | 1.26E-06 | 0.000129652 | 9.66E-05 | PTGS2/TPPP3/RGS2/JUNB/CYP27B1/PTN | 6 |
| BP | GO:0002430 | complement receptor mediated signaling pathway | 4/111 | 15/18888 | 1.47E-06 | 0.00014545 | 0.000108326 | CR1/FPR1/FPR3/C3 | 4 |
| BP | GO:0070555 | response to interleukin-1 | 8/111 | 138/18888 | 1.55E-06 | 0.000148299 | 0.000110447 | EGR1/CHI3L1/PCK1/MMP2/CCL2/CCL18/CCL19/CCL21 | 8 |
| BP | GO:0002237 | response to molecule of bacterial origin | 12/111 | 369/18888 | 1.80E-06 | 0.000160515 | 0.000119546 | FOS/ZFP36/S100A9/PTGS2/UMOD/S100A8/HPGD/PCK1/CYP27B1/PLCG2/MRC1/CCL2 | 12 |
| BP | GO:0001893 | maternal placenta development | 5/111 | 35/18888 | 1.81E-06 | 0.000160515 | 0.000119546 | PTGS2/TPPP3/JUNB/CYP27B1/PTN | 5 |
| BP | GO:0006898 | receptor-mediated endocytosis | 10/111 | 259/18888 | 3.02E-06 | 0.000253904 | 0.000189098 | CXCR2/EGF/CXCR1/MAGI2/PLCG2/MRC1/APOC1/CCL19/CCL21/C3 | 10 |
| BP | GO:0009636 | response to toxic substance | 10/111 | 260/18888 | 3.13E-06 | 0.000253904 | 0.000189098 | FOS/ALB/S100A9/PTGS2/GSTA1/HBB/GLYAT/FABP1/APOM/FBLN5 | 10 |
| BP | GO:0006957 | complement activation, alternative pathway | 4/111 | 18/18888 | 3.24E-06 | 0.000253904 | 0.000189098 | CR1/VSIG4/C7/C3 | 4 |
| BP | GO:0034612 | response to tumor necrosis factor | 10/111 | 263/18888 | 3.46E-06 | 0.000253904 | 0.000189098 | FOS/ZFP36/PTGS2/UMOD/CHI3L1/PCK1/CCL2/CCL18/CCL19/CCL21 | 10 |
| BP | GO:0002548 | monocyte chemotaxis | 6/111 | 70/18888 | 3.47E-06 | 0.000253904 | 0.000189098 | S100A12/DUSP1/CCL2/CCL18/CCL19/CCL21 | 6 |
| BP | GO:0019216 | regulation of lipid metabolic process | 11/111 | 326/18888 | 3.49E-06 | 0.000253904 | 0.000189098 | PDK4/EGR1/PTGS2/HPGD/PCK1/FABP1/CYP27B1/APOD/PLCG2/APOC1/C3 | 11 |
| BP | GO:0071674 | mononuclear cell migration | 9/111 | 206/18888 | 3.55E-06 | 0.000253904 | 0.000189098 | S100A12/DUSP1/CXCR2/CXCR1/APOD/CCL2/CCL18/CCL19/CCL21 | 9 |
| BP | GO:0071347 | cellular response to interleukin-1 | 7/111 | 110/18888 | 3.87E-06 | 0.000263816 | 0.00019648 | EGR1/PCK1/MMP2/CCL2/CCL18/CCL19/CCL21 | 7 |
| BP | GO:0031960 | response to corticosteroid | 8/111 | 156/18888 | 3.89E-06 | 0.000263816 | 0.00019648 | FOS/ZFP36/PTGS2/SLC12A3/PTGDS/PCK1/FOSB/SERPINF1 | 8 |
| BP | GO:0098754 | detoxification | 8/111 | 158/18888 | 4.27E-06 | 0.000282545 | 0.000210429 | ALB/S100A9/PTGS2/GSTA1/HBB/FABP1/APOM/FBLN5 | 8 |
| BP | GO:0048545 | response to steroid hormone | 11/111 | 338/18888 | 4.92E-06 | 0.000317379 | 0.000236372 | FOS/ZFP36/PTGS2/LOX/SLC12A3/PTGDS/SST/PCK1/TCF21/FOSB/SERPINF1 | 11 |
| BP | GO:0048259 | regulation of receptor-mediated endocytosis | 7/111 | 115/18888 | 5.20E-06 | 0.000326984 | 0.000243526 | EGF/MAGI2/PLCG2/APOC1/CCL19/CCL21/C3 | 7 |
| BP | GO:1901654 | response to ketone | 9/111 | 217/18888 | 5.42E-06 | 0.000332469 | 0.000247611 | FOS/SLC12A3/FBP1/PCK1/FOSB/TNC/SERPINF1/CCL19/CCL21 | 9 |
| BP | GO:0030282 | bone mineralization | 7/111 | 127/18888 | 1.00E-05 | 0.000600173 | 0.000446987 | PTGS2/LOX/IGF1/CYP27B1/PTN/ASPN/COL1A2 | 7 |
| BP | GO:0002407 | dendritic cell chemotaxis | 4/111 | 24/18888 | 1.10E-05 | 0.000642323 | 0.000478378 | CXCR2/CXCR1/CCL19/CCL21 | 4 |
| BP | GO:0009410 | response to xenobiotic stimulus | 12/111 | 444/18888 | 1.18E-05 | 0.000677477 | 0.00050456 | FOS/S100A12/PTGS2/LOX/GSTA1/UMOD/GLYAT/SST/FBP1/FOSB/CYP2B6/MMP2 | 12 |
| BP | GO:0001503 | ossification | 12/111 | 446/18888 | 1.24E-05 | 0.000691103 | 0.000514708 | PTGS2/LOX/JUNB/IGF1/CYP27B1/FGF9/MMP2/TNC/PTN/ASPN/COL1A2/VCAN | 12 |
| BP | GO:0071356 | cellular response to tumor necrosis factor | 9/111 | 241/18888 | 1.26E-05 | 0.000691103 | 0.000514708 | FOS/ZFP36/UMOD/CHI3L1/PCK1/CCL2/CCL18/CCL19/CCL21 | 9 |
| BP | GO:0051384 | response to glucocorticoid | 7/111 | 134/18888 | 1.42E-05 | 0.000763604 | 0.000568704 | FOS/ZFP36/PTGS2/PTGDS/PCK1/FOSB/SERPINF1 | 7 |
| BP | GO:0006006 | glucose metabolic process | 8/111 | 192/18888 | 1.78E-05 | 0.000932844 | 0.000694748 | PDK4/IGF1/ALDOB/FBP1/DCXR/PCK1/APOD/ATF3 | 8 |
| BP | GO:0070098 | chemokine-mediated signaling pathway | 6/111 | 93/18888 | 1.81E-05 | 0.000932844 | 0.000694748 | CXCR2/CXCR1/CCL2/CCL18/CCL19/CCL21 | 6 |
| BP | GO:0001822 | kidney development | 10/111 | 319/18888 | 1.87E-05 | 0.00094703 | 0.000705313 | EGR1/NPHS1/UMOD/HPGD/CXCR2/TCF21/MAGI2/CYP4A11/CALB1/SERPINF1 | 10 |
| BP | GO:0014012 | peripheral nervous system axon regeneration | 3/111 | 10/18888 | 2.30E-05 | 0.001078194 | 0.000803 | APOD/MMP2/TNC | 3 |
| BP | GO:0048251 | elastic fiber assembly | 3/111 | 10/18888 | 2.30E-05 | 0.001078194 | 0.000803 | LOX/MFAP4/FBLN5 | 3 |
| BP | GO:1904627 | response to phorbol 13-acetate 12-myristate | 3/111 | 10/18888 | 2.30E-05 | 0.001078194 | 0.000803 | FOS/FBP1/PCK1 | 3 |
| BP | GO:1904628 | cellular response to phorbol 13-acetate 12-myristate | 3/111 | 10/18888 | 2.30E-05 | 0.001078194 | 0.000803 | FOS/FBP1/PCK1 | 3 |
| BP | GO:0072001 | renal system development | 10/111 | 329/18888 | 2.44E-05 | 0.001123338 | 0.000836621 | EGR1/NPHS1/UMOD/HPGD/CXCR2/TCF21/MAGI2/CYP4A11/CALB1/SERPINF1 | 10 |
| BP | GO:0062012 | regulation of small molecule metabolic process | 10/111 | 331/18888 | 2.57E-05 | 0.00116218 | 0.000865549 | PDK4/EGR1/PTGS2/IGF1/ALDOB/FBP1/FABP1/BHMT/CYP27B1/APOC1 | 10 |
| BP | GO:0030198 | extracellular matrix organization | 10/111 | 332/18888 | 2.64E-05 | 0.001171882 | 0.000872774 | LOX/NPHS1/AEBP1/MFAP4/MMP2/TGFBI/FBLN5/COL1A2/MMP7/LUM | 10 |
| BP | GO:0043062 | extracellular structure organization | 10/111 | 333/18888 | 2.70E-05 | 0.001181906 | 0.00088024 | LOX/NPHS1/AEBP1/MFAP4/MMP2/TGFBI/FBLN5/COL1A2/MMP7/LUM | 10 |
| BP | GO:0045229 | external encapsulating structure organization | 10/111 | 334/18888 | 2.77E-05 | 0.001192248 | 0.000887942 | LOX/NPHS1/AEBP1/MFAP4/MMP2/TGFBI/FBLN5/COL1A2/MMP7/LUM | 10 |
| BP | GO:0071496 | cellular response to external stimulus | 10/111 | 335/18888 | 2.85E-05 | 0.001202903 | 0.000895878 | FOS/ALB/PDK4/PTGS2/LPL/PCK1/NR4A2/CYP27B1/ATF3/TNC | 10 |
| BP | GO:0031668 | cellular response to extracellular stimulus | 9/111 | 269/18888 | 3.00E-05 | 0.001249119 | 0.000930298 | FOS/ALB/PDK4/LPL/PCK1/NR4A2/CYP27B1/ATF3/TNC | 9 |
| BP | GO:0036336 | dendritic cell migration | 4/111 | 31/18888 | 3.14E-05 | 0.001286941 | 0.000958467 | CXCR2/CXCR1/CCL19/CCL21 | 4 |
| BP | GO:0016042 | lipid catabolic process | 10/111 | 340/18888 | 3.23E-05 | 0.00130018 | 0.000968326 | LPL/HAO2/HPGD/PCK1/FABP1/HSD17B14/CYP27B1/CYP4A11/PLCG2/APOC1 | 10 |
| BP | GO:0050729 | positive regulation of inflammatory response | 7/111 | 153/18888 | 3.35E-05 | 0.001328453 | 0.000989384 | S100A12/S100A9/PTGS2/LPL/S100A8/PLCG2/C3 | 7 |
| BP | GO:0045807 | positive regulation of endocytosis | 7/111 | 155/18888 | 3.64E-05 | 0.001421722 | 0.001058847 | EGF/MAGI2/PLCG2/CCL2/CCL19/CCL21/C3 | 7 |
| BP | GO:0120254 | olefinic compound metabolic process | 7/111 | 162/18888 | 4.82E-05 | 0.001855984 | 0.001382269 | EGR1/PTGS2/GSTA1/PTGDS/CYP2B6/CYP4A11/ADH1B | 7 |
| BP | GO:0007517 | muscle organ development | 10/111 | 359/18888 | 5.10E-05 | 0.00193499 | 0.00144111 | FOS/EGR1/LOX/NPHS1/TNNI1/IGF1/TCF21/TNNT2/ATF3/COL6A3 | 10 |
| BP | GO:0060537 | muscle tissue development | 11/111 | 438/18888 | 5.43E-05 | 0.002028753 | 0.001510941 | FOS/EGR1/LOX/NPHS1/RGS2/TNNI1/IGF1/TCF21/TNNT2/ATF3/FGF9 | 11 |
| BP | GO:0033559 | unsaturated fatty acid metabolic process | 6/111 | 115/18888 | 6.02E-05 | 0.00221555 | 0.00165006 | PTGS2/GSTA1/HPGD/PTGDS/CYP2B6/CYP4A11 | 6 |
| BP | GO:0071241 | cellular response to inorganic substance | 8/111 | 229/18888 | 6.24E-05 | 0.002267151 | 0.001688491 | FOS/PTGS2/SLC12A3/JUNB/FBP1/FOSB/PLCG2/SERPINF1 | 8 |
| BP | GO:0043491 | phosphatidylinositol 3-kinase/protein kinase B signal transduction | 9/111 | 299/18888 | 6.82E-05 | 0.002415645 | 0.001799084 | LOX/IGF1/CHI3L1/EGF/MAGI2/COL6A3/CCL19/CCL21/FN1 | 9 |
| BP | GO:0019318 | hexose metabolic process | 8/111 | 232/18888 | 6.84E-05 | 0.002415645 | 0.001799084 | PDK4/IGF1/ALDOB/FBP1/DCXR/PCK1/APOD/ATF3 | 8 |
| BP | GO:0031669 | cellular response to nutrient levels | 8/111 | 239/18888 | 8.42E-05 | 0.002932667 | 0.002184143 | FOS/ALB/PDK4/LPL/PCK1/CYP27B1/ATF3/TNC | 8 |
| BP | GO:0002688 | regulation of leukocyte chemotaxis | 6/111 | 123/18888 | 8.75E-05 | 0.003006602 | 0.002239207 | DUSP1/CXCR2/PTN/CCL2/CCL19/CCL21 | 6 |
| BP | GO:0007162 | negative regulation of cell adhesion | 9/111 | 310/18888 | 8.98E-05 | 0.003047274 | 0.002269498 | DUSP1/KNG1/CR1/APOD/MMP2/TNC/TGFBI/VSIG4/CCL21 | 9 |
| BP | GO:0031214 | biomineral tissue development | 7/111 | 180/18888 | 9.37E-05 | 0.003138316 | 0.002337303 | PTGS2/LOX/IGF1/CYP27B1/PTN/ASPN/COL1A2 | 7 |
| BP | GO:0006690 | icosanoid metabolic process | 6/111 | 125/18888 | 9.56E-05 | 0.003161002 | 0.002354199 | PTGS2/GSTA1/HPGD/PTGDS/CYP2B6/CYP4A11 | 6 |
| BP | GO:0046890 | regulation of lipid biosynthetic process | 7/111 | 187/18888 | 0.000118916 | 0.003880571 | 0.002890107 | PDK4/EGR1/PTGS2/PCK1/CYP27B1/APOC1/C3 | 7 |
| BP | GO:0005996 | monosaccharide metabolic process | 8/111 | 252/18888 | 0.000121507 | 0.003915577 | 0.002916178 | PDK4/IGF1/ALDOB/FBP1/DCXR/PCK1/APOD/ATF3 | 8 |
| BP | GO:0034375 | high-density lipoprotein particle remodeling | 3/111 | 17/18888 | 0.000126494 | 0.004025942 | 0.002998374 | LPL/APOM/APOC1 | 3 |
| BP | GO:0006959 | humoral immune response | 8/111 | 258/18888 | 0.000142857 | 0.00449127 | 0.003344934 | S100A12/S100A9/CR1/RNASE6/VSIG4/CCL2/C7/C3 | 8 |
| BP | GO:0070371 | ERK1 and ERK2 cascade | 9/111 | 332/18888 | 0.000150796 | 0.00468376 | 0.003488293 | IGF1/CHI3L1/EGF/ATF3/CCL2/CCL18/CCL19/CCL21/FN1 | 9 |
| BP | GO:0048678 | response to axon injury | 5/111 | 86/18888 | 0.000152696 | 0.004686306 | 0.003490189 | APOD/PLCG2/MMP2/TNC/PTN | 5 |
| BP | GO:0002523 | leukocyte migration involved in inflammatory response | 3/111 | 19/18888 | 0.000178716 | 0.005391661 | 0.004015512 | S100A9/S100A8/PTN | 3 |
| BP | GO:0071248 | cellular response to metal ion | 7/111 | 200/18888 | 0.000180184 | 0.005391661 | 0.004015512 | FOS/PTGS2/JUNB/FBP1/FOSB/PLCG2/SERPINF1 | 7 |
| BP | GO:0007159 | leukocyte cell-cell adhesion | 10/111 | 419/18888 | 0.000181953 | 0.005391661 | 0.004015512 | S100A9/UMOD/S100A8/IGF1/PCK1/CR1/VSIG4/CCL2/CCL19/CCL21 | 10 |
| BP | GO:0034341 | response to type II interferon | 6/111 | 142/18888 | 0.000192451 | 0.005637947 | 0.004198936 | CYP27B1/MRC1/CCL2/CCL18/CCL19/CCL21 | 6 |
| BP | GO:0050727 | regulation of inflammatory response | 10/111 | 425/18888 | 0.00020402 | 0.005909713 | 0.004401338 | ZFP36/S100A12/S100A9/PTGS2/LPL/S100A8/IGF1/PLCG2/TNC/C3 | 10 |
| BP | GO:0010565 | regulation of cellular ketone metabolic process | 6/111 | 144/18888 | 0.000207637 | 0.005947642 | 0.004429586 | PDK4/EGR1/PTGS2/FABP1/BHMT/APOC1 | 6 |
| BP | GO:0006693 | prostaglandin metabolic process | 4/111 | 50/18888 | 0.00021125 | 0.005984633 | 0.004457135 | PTGS2/GSTA1/HPGD/PTGDS | 4 |
| BP | GO:0016051 | carbohydrate biosynthetic process | 7/111 | 206/18888 | 0.000215993 | 0.006052487 | 0.004507671 | IGF1/EGF/ALDOB/FBP1/PCK1/B3GALT2/ATF3 | 7 |
| BP | GO:0001656 | metanephros development | 5/111 | 93/18888 | 0.000220568 | 0.006114249 | 0.004553669 | EGR1/UMOD/CXCR2/TCF21/CALB1 | 5 |
| BP | GO:0006692 | prostanoid metabolic process | 4/111 | 51/18888 | 0.000228196 | 0.006258409 | 0.004661034 | PTGS2/GSTA1/HPGD/PTGDS | 4 |
| BP | GO:1904707 | positive regulation of vascular associated smooth muscle cell proliferation | 4/111 | 52/18888 | 0.0002461 | 0.006678382 | 0.004973814 | IGF1/HPGD/FGF9/MMP2 | 4 |
| BP | GO:0001890 | placenta development | 6/111 | 150/18888 | 0.000258935 | 0.006953485 | 0.005178701 | PTGS2/TPPP3/JUNB/CYP27B1/KRT19/PTN | 6 |
| BP | GO:0010038 | response to metal ion | 9/111 | 360/18888 | 0.000275114 | 0.007301842 | 0.005438144 | FOS/PTGS2/S100A8/JUNB/FBP1/TNNT2/FOSB/PLCG2/SERPINF1 | 9 |
| BP | GO:0072073 | kidney epithelium development | 6/111 | 152/18888 | 0.000278085 | 0.007301842 | 0.005438144 | NPHS1/UMOD/CXCR2/TCF21/MAGI2/CALB1 | 6 |
| BP | GO:0030449 | regulation of complement activation | 3/111 | 22/18888 | 0.000280404 | 0.007301842 | 0.005438144 | CR1/VSIG4/C3 | 3 |
| BP | GO:0031103 | axon regeneration | 4/111 | 54/18888 | 0.000284904 | 0.00734482 | 0.005470153 | APOD/MMP2/TNC/PTN | 4 |
| BP | GO:0009612 | response to mechanical stimulus | 7/111 | 216/18888 | 0.000288283 | 0.007358364 | 0.00548024 | FOS/PTGS2/CHI3L1/FOSB/MMP2/TNC/PTN | 7 |
| BP | GO:0002686 | negative regulation of leukocyte migration | 4/111 | 55/18888 | 0.000305867 | 0.007655583 | 0.005701598 | DUSP1/APOD/CCL2/CCL21 | 4 |
| BP | GO:0085029 | extracellular matrix assembly | 4/111 | 55/18888 | 0.000305867 | 0.007655583 | 0.005701598 | LOX/MFAP4/FBLN5/COL1A2 | 4 |
| BP | GO:0048661 | positive regulation of smooth muscle cell proliferation | 5/111 | 100/18888 | 0.000309295 | 0.00766695 | 0.005710064 | PTGS2/IGF1/HPGD/FGF9/MMP2 | 5 |
| BP | GO:0043086 | negative regulation of catalytic activity | 10/111 | 449/18888 | 0.000316182 | 0.007763028 | 0.005781619 | ZFP36/RGS2/DUSP1/PRKAR2B/KNG1/FABP1/CR1/CYP27B1/SERPINF1/APOC1 | 10 |
| BP | GO:0010560 | positive regulation of glycoprotein biosynthetic process | 3/111 | 23/18888 | 0.000321088 | 0.007783375 | 0.005796773 | IGF1/CCL19/CCL21 | 3 |
| BP | GO:0030100 | regulation of endocytosis | 8/111 | 291/18888 | 0.000323049 | 0.007783375 | 0.005796773 | EGF/MAGI2/PLCG2/APOC1/CCL2/CCL19/CCL21/C3 | 8 |
| BP | GO:0050832 | defense response to fungus | 4/111 | 56/18888 | 0.000327914 | 0.007827419 | 0.005829575 | S100A12/S100A9/S100A8/PLCG2 | 4 |
| BP | GO:0072006 | nephron development | 6/111 | 158/18888 | 0.000342287 | 0.008095561 | 0.006029277 | EGR1/NPHS1/UMOD/TCF21/MAGI2/CALB1 | 6 |
| BP | GO:0044282 | small molecule catabolic process | 9/111 | 373/18888 | 0.000356688 | 0.008359462 | 0.006225821 | HPD/HAO2/ALDOB/DCXR/PCK1/FABP1/CYP27B1/PRODH2/CYP4A11 | 9 |
| BP | GO:0019369 | arachidonic acid metabolic process | 4/111 | 58/18888 | 0.000375386 | 0.008718421 | 0.006493161 | PTGS2/PTGDS/CYP2B6/CYP4A11 | 4 |
| BP | GO:0042180 | cellular ketone metabolic process | 7/111 | 227/18888 | 0.000389067 | 0.008955478 | 0.006669712 | PDK4/EGR1/PTGS2/FABP1/BHMT/CYP2B6/APOC1 | 7 |
| BP | GO:0071676 | negative regulation of mononuclear cell migration | 3/111 | 25/18888 | 0.000413447 | 0.009158726 | 0.006821084 | DUSP1/APOD/CCL2 | 3 |
| BP | GO:0072170 | metanephric tubule development | 3/111 | 25/18888 | 0.000413447 | 0.009158726 | 0.006821084 | UMOD/CXCR2/CALB1 | 3 |
| BP | GO:0090023 | positive regulation of neutrophil chemotaxis | 3/111 | 25/18888 | 0.000413447 | 0.009158726 | 0.006821084 | CXCR2/CCL19/CCL21 | 3 |
| BP | GO:0007584 | response to nutrient | 6/111 | 164/18888 | 0.000417566 | 0.009158726 | 0.006821084 | PTGS2/LPL/SST/FABP1/CYP27B1/TNC | 6 |
| BP | GO:0002685 | regulation of leukocyte migration | 7/111 | 230/18888 | 0.000420951 | 0.009158726 | 0.006821084 | DUSP1/CXCR2/APOD/PTN/CCL2/CCL19/CCL21 | 7 |
| BP | GO:0044242 | cellular lipid catabolic process | 7/111 | 230/18888 | 0.000420951 | 0.009158726 | 0.006821084 | LPL/HAO2/PCK1/FABP1/CYP4A11/PLCG2/APOC1 | 7 |
| BP | GO:1901655 | cellular response to ketone | 5/111 | 107/18888 | 0.000422765 | 0.009158726 | 0.006821084 | FOS/FBP1/PCK1/TNC/SERPINF1 | 5 |
| BP | GO:0006641 | triglyceride metabolic process | 5/111 | 108/18888 | 0.000441234 | 0.009479188 | 0.007059752 | LPL/PCK1/APOH/APOC1/C3 | 5 |
| BP | GO:0031102 | neuron projection regeneration | 4/111 | 61/18888 | 0.000455529 | 0.009598012 | 0.007148248 | APOD/MMP2/TNC/PTN | 4 |
| BP | GO:0001676 | long-chain fatty acid metabolic process | 5/111 | 109/18888 | 0.000460306 | 0.009598012 | 0.007148248 | PTGS2/GSTA1/PTGDS/CYP2B6/CYP4A11 | 5 |
| BP | GO:0002526 | acute inflammatory response | 5/111 | 109/18888 | 0.000460306 | 0.009598012 | 0.007148248 | PTGS2/CXCR2/FCGR3B/C3/FN1 | 5 |
| BP | GO:0002092 | positive regulation of receptor internalization | 3/111 | 26/18888 | 0.000465381 | 0.009598012 | 0.007148248 | EGF/MAGI2/PLCG2 | 3 |
| BP | GO:1903020 | positive regulation of glycoprotein metabolic process | 3/111 | 26/18888 | 0.000465381 | 0.009598012 | 0.007148248 | IGF1/CCL19/CCL21 | 3 |
| BP | GO:0070372 | regulation of ERK1 and ERK2 cascade | 8/111 | 308/18888 | 0.000471221 | 0.009641335 | 0.007180513 | IGF1/CHI3L1/ATF3/CCL2/CCL18/CCL19/CCL21/FN1 | 8 |
| BP | GO:0062197 | cellular response to chemical stress | 8/111 | 311/18888 | 0.000502343 | 0.010181549 | 0.007582845 | FOS/PTGS2/FBP1/PCK1/FABP1/NR4A2/MMP2/FBLN5 | 8 |
| BP | GO:0032623 | interleukin-2 production | 4/111 | 63/18888 | 0.000515295 | 0.010181549 | 0.007582845 | ZFP36/CR1/PLCG2/VSIG4 | 4 |
| BP | GO:0032663 | regulation of interleukin-2 production | 4/111 | 63/18888 | 0.000515295 | 0.010181549 | 0.007582845 | ZFP36/CR1/PLCG2/VSIG4 | 4 |
| BP | GO:0051897 | positive regulation of phosphatidylinositol 3-kinase/protein kinase B signal transduction | 6/111 | 171/18888 | 0.000521102 | 0.010181549 | 0.007582845 | IGF1/CHI3L1/EGF/CCL19/CCL21/FN1 | 6 |
| BP | GO:0006972 | hyperosmotic response | 3/111 | 27/18888 | 0.000521321 | 0.010181549 | 0.007582845 | SST/FBP1/PCK1 | 3 |
| BP | GO:0071624 | positive regulation of granulocyte chemotaxis | 3/111 | 27/18888 | 0.000521321 | 0.010181549 | 0.007582845 | CXCR2/CCL19/CCL21 | 3 |
| BP | GO:0009743 | response to carbohydrate | 7/111 | 239/18888 | 0.000529334 | 0.010260326 | 0.007641515 | EGR1/PTGS2/LPL/FBP1/PCK1/SERPINF1/COL6A3 | 7 |
| BP | GO:0048247 | lymphocyte chemotaxis | 4/111 | 64/18888 | 0.000547179 | 0.010527076 | 0.007840181 | CCL2/CCL18/CCL19/CCL21 | 4 |
| BP | GO:0048660 | regulation of smooth muscle cell proliferation | 6/111 | 174/18888 | 0.000571154 | 0.01090693 | 0.008123082 | PTGS2/IGF1/HPGD/APOD/FGF9/MMP2 | 6 |
| BP | GO:0032703 | negative regulation of interleukin-2 production | 3/111 | 28/18888 | 0.00058139 | 0.011020754 | 0.008207854 | ZFP36/CR1/VSIG4 | 3 |
| BP | GO:0030199 | collagen fibril organization | 4/111 | 66/18888 | 0.000615114 | 0.01157492 | 0.008620577 | LOX/AEBP1/COL1A2/LUM | 4 |
| BP | GO:0048659 | smooth muscle cell proliferation | 6/111 | 178/18888 | 0.000643615 | 0.011906917 | 0.008867836 | PTGS2/IGF1/HPGD/APOD/FGF9/MMP2 | 6 |
| BP | GO:0072207 | metanephric epithelium development | 3/111 | 29/18888 | 0.000645709 | 0.011906917 | 0.008867836 | UMOD/CXCR2/CALB1 | 3 |
| BP | GO:0006956 | complement activation | 4/111 | 67/18888 | 0.000651232 | 0.011906917 | 0.008867836 | CR1/VSIG4/C7/C3 | 4 |
| BP | GO:0009620 | response to fungus | 4/111 | 67/18888 | 0.000651232 | 0.011906917 | 0.008867836 | S100A12/S100A9/S100A8/PLCG2 | 4 |
| BP | GO:0006090 | pyruvate metabolic process | 5/111 | 118/18888 | 0.000661168 | 0.012003466 | 0.008939742 | PDK4/IGF1/ALDOB/FBP1/PCK1 | 5 |
| BP | GO:0016054 | organic acid catabolic process | 7/111 | 250/18888 | 0.000690833 | 0.01236783 | 0.009211107 | HPD/HAO2/DCXR/PCK1/FABP1/PRODH2/CYP4A11 | 7 |
| BP | GO:0046395 | carboxylic acid catabolic process | 7/111 | 250/18888 | 0.000690833 | 0.01236783 | 0.009211107 | HPD/HAO2/DCXR/PCK1/FABP1/PRODH2/CYP4A11 | 7 |
| BP | GO:0033002 | muscle cell proliferation | 7/111 | 251/18888 | 0.00070726 | 0.012528679 | 0.009330901 | FOS/PTGS2/IGF1/HPGD/APOD/FGF9/MMP2 | 7 |
| BP | GO:0071346 | cellular response to type II interferon | 5/111 | 120/18888 | 0.000713527 | 0.012528679 | 0.009330901 | MRC1/CCL2/CCL18/CCL19/CCL21 | 5 |
| BP | GO:0002438 | acute inflammatory response to antigenic stimulus | 3/111 | 30/18888 | 0.000714397 | 0.012528679 | 0.009330901 | CXCR2/FCGR3B/C3 | 3 |
| BP | GO:0072009 | nephron epithelium development | 5/111 | 121/18888 | 0.00074084 | 0.012821562 | 0.00954903 | NPHS1/UMOD/TCF21/MAGI2/CALB1 | 5 |
| BP | GO:0051896 | regulation of phosphatidylinositol 3-kinase/protein kinase B signal transduction | 7/111 | 253/18888 | 0.000741045 | 0.012821562 | 0.00954903 | IGF1/CHI3L1/EGF/MAGI2/CCL19/CCL21/FN1 | 7 |
| BP | GO:0045471 | response to ethanol | 5/111 | 122/18888 | 0.000768927 | 0.013215293 | 0.009842266 | FOS/RGS2/S100A8/HPGD/TNC | 5 |
| BP | GO:0032835 | glomerulus development | 4/111 | 71/18888 | 0.000810818 | 0.013842974 | 0.01030974 | EGR1/NPHS1/TCF21/MAGI2 | 4 |
| BP | GO:0008217 | regulation of blood pressure | 6/111 | 187/18888 | 0.0008327 | 0.014123023 | 0.01051831 | PTGS2/UMOD/HBB/CYP4A11/CPA3/COL1A2 | 6 |
| BP | GO:0016053 | organic acid biosynthetic process | 8/111 | 337/18888 | 0.000848283 | 0.01427003 | 0.010627795 | PDK4/PTGS2/LPL/PTGDS/BHMT/CYP4A11/FMO3/APOC1 | 8 |
| BP | GO:0042698 | ovulation cycle | 4/111 | 72/18888 | 0.000854662 | 0.01427003 | 0.010627795 | EGR1/MMP2/PTN/SERPINF1 | 4 |
| BP | GO:0072676 | lymphocyte migration | 5/111 | 125/18888 | 0.000857973 | 0.01427003 | 0.010627795 | APOD/CCL2/CCL18/CCL19/CCL21 | 5 |
| BP | GO:0034694 | response to prostaglandin | 3/111 | 32/18888 | 0.000865341 | 0.014300322 | 0.010650356 | TNC/CCL19/CCL21 | 3 |
| BP | GO:0031623 | receptor internalization | 5/111 | 127/18888 | 0.000921464 | 0.015130794 | 0.011268861 | CXCR2/EGF/CXCR1/MAGI2/PLCG2 | 5 |
| BP | GO:0034368 | protein-lipid complex remodeling | 3/111 | 33/18888 | 0.000947822 | 0.015367838 | 0.011445403 | LPL/APOM/APOC1 | 3 |
| BP | GO:0034369 | plasma lipoprotein particle remodeling | 3/111 | 33/18888 | 0.000947822 | 0.015367838 | 0.011445403 | LPL/APOM/APOC1 | 3 |
| BP | GO:0046683 | response to organophosphorus | 5/111 | 128/18888 | 0.00095449 | 0.015379224 | 0.011453883 | FOS/PTGS2/FBP1/PCK1/FOSB | 5 |
| BP | GO:0090022 | regulation of neutrophil chemotaxis | 3/111 | 34/18888 | 0.001035122 | 0.016472494 | 0.012268111 | CXCR2/CCL19/CCL21 | 3 |
| BP | GO:1902624 | positive regulation of neutrophil migration | 3/111 | 34/18888 | 0.001035122 | 0.016472494 | 0.012268111 | CXCR2/CCL19/CCL21 | 3 |
| BP | GO:0072329 | monocarboxylic acid catabolic process | 5/111 | 131/18888 | 0.001058862 | 0.016746916 | 0.01247249 | HAO2/DCXR/PCK1/FABP1/CYP4A11 | 5 |
| BP | GO:0070293 | renal absorption | 3/111 | 35/18888 | 0.001127346 | 0.017721331 | 0.013198199 | UMOD/SLC12A3/HBB | 3 |
| BP | GO:2000242 | negative regulation of reproductive process | 4/111 | 78/18888 | 0.00115347 | 0.018022093 | 0.013422195 | DUSP1/IGF1/PTGDS/SERPINF1 | 4 |
| BP | GO:0043405 | regulation of MAP kinase activity | 5/111 | 134/18888 | 0.001171479 | 0.018193207 | 0.013549634 | S100A12/RGS2/DUSP1/EGF/CCL19 | 5 |
| BP | GO:0034367 | protein-containing complex remodeling | 3/111 | 36/18888 | 0.001224599 | 0.018904296 | 0.014079227 | LPL/APOM/APOC1 | 3 |
| BP | GO:0006639 | acylglycerol metabolic process | 5/111 | 136/18888 | 0.001251331 | 0.019201972 | 0.014300926 | LPL/PCK1/APOH/APOC1/C3 | 5 |
| BP | GO:0000302 | response to reactive oxygen species | 6/111 | 203/18888 | 0.001271796 | 0.019400541 | 0.014448812 | FOS/HBB/FABP1/APOD/MMP2/FBLN5 | 6 |
| BP | GO:0006638 | neutral lipid metabolic process | 5/111 | 137/18888 | 0.001292734 | 0.019516282 | 0.014535012 | LPL/PCK1/APOH/APOC1/C3 | 5 |
| BP | GO:0031346 | positive regulation of cell projection organization | 8/111 | 360/18888 | 0.001294525 | 0.019516282 | 0.014535012 | S100A9/RGS2/MAGI2/PTN/SERPINF1/CCL19/CCL21/FN1 | 8 |
| BP | GO:0051346 | negative regulation of hydrolase activity | 6/111 | 204/18888 | 0.00130417 | 0.019547385 | 0.014558177 | RGS2/KNG1/FABP1/CR1/SERPINF1/APOC1 | 6 |
| BP | GO:0033280 | response to vitamin D | 3/111 | 37/18888 | 0.001326984 | 0.019666437 | 0.014646842 | PTGS2/CYP27B1/TNC | 3 |
| BP | GO:0006970 | response to osmotic stress | 4/111 | 81/18888 | 0.00132737 | 0.019666437 | 0.014646842 | PTGS2/SST/FBP1/PCK1 | 4 |
| BP | GO:0001101 | response to acid chemical | 5/111 | 139/18888 | 0.001378566 | 0.020308248 | 0.01512484 | UMOD/SST/PCK1/MMP2/COL1A2 | 5 |
| BP | GO:0042401 | biogenic amine biosynthetic process | 3/111 | 38/18888 | 0.001434598 | 0.020973754 | 0.015620484 | AGMAT/NR4A2/MOXD1 | 3 |
| BP | GO:0009746 | response to hexose | 6/111 | 208/18888 | 0.001440013 | 0.020973754 | 0.015620484 | EGR1/PTGS2/LPL/PCK1/SERPINF1/COL6A3 | 6 |
| BP | GO:0001794 | type IIa hypersensitivity | 2/111 | 10/18888 | 0.001493503 | 0.021039626 | 0.015669543 | FCGR3B/C3 | 2 |
| BP | GO:0002445 | type II hypersensitivity | 2/111 | 10/18888 | 0.001493503 | 0.021039626 | 0.015669543 | FCGR3B/C3 | 2 |
| BP | GO:0009750 | response to fructose | 2/111 | 10/18888 | 0.001493503 | 0.021039626 | 0.015669543 | PTGS2/PCK1 | 2 |
| BP | GO:0042538 | hyperosmotic salinity response | 2/111 | 10/18888 | 0.001493503 | 0.021039626 | 0.015669543 | FBP1/PCK1 | 2 |
| BP | GO:0051238 | sequestering of metal ion | 2/111 | 10/18888 | 0.001493503 | 0.021039626 | 0.015669543 | S100A9/S100A8 | 2 |
| BP | GO:2000425 | regulation of apoptotic cell clearance | 2/111 | 10/18888 | 0.001493503 | 0.021039626 | 0.015669543 | CCL2/C3 | 2 |
| BP | GO:0043542 | endothelial cell migration | 7/111 | 286/18888 | 0.001507645 | 0.021123415 | 0.015731946 | S100A12/S100A9/PTGS2/EGF/APOH/PTN/SERPINF1 | 7 |
| BP | GO:0009309 | amine biosynthetic process | 3/111 | 39/18888 | 0.001547541 | 0.021544331 | 0.016045429 | AGMAT/NR4A2/MOXD1 | 3 |
| BP | GO:0014074 | response to purine-containing compound | 5/111 | 143/18888 | 0.001562758 | 0.021544331 | 0.016045429 | FOS/PTGS2/FBP1/PCK1/FOSB | 5 |
| BP | GO:2001235 | positive regulation of apoptotic signaling pathway | 5/111 | 143/18888 | 0.001562758 | 0.021544331 | 0.016045429 | S100A9/S100A8/G0S2/ATF3/MMP2 | 5 |
| BP | GO:0042594 | response to starvation | 6/111 | 212/18888 | 0.001586404 | 0.021642634 | 0.016118642 | FOS/ZFP36/ALB/PDK4/PCK1/ATF3 | 6 |
| BP | GO:0051591 | response to cAMP | 4/111 | 85/18888 | 0.001586679 | 0.021642634 | 0.016118642 | FOS/FBP1/PCK1/FOSB | 4 |
| BP | GO:0051385 | response to mineralocorticoid | 3/111 | 40/18888 | 0.001665906 | 0.022485368 | 0.016746279 | FOS/SLC12A3/FOSB | 3 |
| BP | GO:0150077 | regulation of neuroinflammatory response | 3/111 | 40/18888 | 0.001665906 | 0.022485368 | 0.016746279 | PTGS2/IGF1/PLCG2 | 3 |
| BP | GO:0010631 | epithelial cell migration | 8/111 | 375/18888 | 0.001674677 | 0.022486025 | 0.016746768 | S100A12/S100A9/PTGS2/EGF/APOH/PLCG2/PTN/SERPINF1 | 8 |
| BP | GO:0034284 | response to monosaccharide | 6/111 | 215/18888 | 0.001703434 | 0.022636357 | 0.01685873 | EGR1/PTGS2/LPL/PCK1/SERPINF1/COL6A3 | 6 |
| BP | GO:0070374 | positive regulation of ERK1 and ERK2 cascade | 6/111 | 215/18888 | 0.001703434 | 0.022636357 | 0.01685873 | IGF1/CHI3L1/CCL2/CCL18/CCL19/CCL21 | 6 |
| BP | GO:0051592 | response to calcium ion | 5/111 | 146/18888 | 0.001712337 | 0.022637969 | 0.016859931 | FOS/JUNB/TNNT2/FOSB/PLCG2 | 5 |
| BP | GO:0071277 | cellular response to calcium ion | 4/111 | 87/18888 | 0.001728695 | 0.022737637 | 0.016934159 | FOS/JUNB/FOSB/PLCG2 | 4 |
| BP | GO:0090132 | epithelium migration | 8/111 | 378/18888 | 0.001760364 | 0.023036642 | 0.017156847 | S100A12/S100A9/PTGS2/EGF/APOH/PLCG2/PTN/SERPINF1 | 8 |
| BP | GO:0071634 | regulation of transforming growth factor beta production | 3/111 | 41/18888 | 0.001789787 | 0.023092715 | 0.017198608 | PTGS2/LUM/FN1 | 3 |
| BP | GO:0002604 | regulation of dendritic cell antigen processing and presentation | 2/111 | 11/18888 | 0.001818395 | 0.023092715 | 0.017198608 | CCL19/CCL21 | 2 |
| BP | GO:0007614 | short-term memory | 2/111 | 11/18888 | 0.001818395 | 0.023092715 | 0.017198608 | CALB1/SERPINF1 | 2 |
| BP | GO:0071476 | cellular hypotonic response | 2/111 | 11/18888 | 0.001818395 | 0.023092715 | 0.017198608 | FBP1/PCK1 | 2 |
| BP | GO:0072015 | podocyte development | 2/111 | 11/18888 | 0.001818395 | 0.023092715 | 0.017198608 | NPHS1/MAGI2 | 2 |
| BP | GO:0097278 | complement-dependent cytotoxicity | 2/111 | 11/18888 | 0.001818395 | 0.023092715 | 0.017198608 | CR1/C3 | 2 |
| BP | GO:0030336 | negative regulation of cell migration | 8/111 | 381/18888 | 0.001849489 | 0.023372464 | 0.017406955 | DUSP1/APOH/DACH1/MAGI2/APOD/SERPINF1/CCL2/CCL21 | 8 |
| BP | GO:0006094 | gluconeogenesis | 4/111 | 89/18888 | 0.001879308 | 0.023527101 | 0.017522124 | ALDOB/FBP1/PCK1/ATF3 | 4 |
| BP | GO:0050863 | regulation of T cell activation | 8/111 | 382/18888 | 0.001879978 | 0.023527101 | 0.017522124 | JUNB/IGF1/PCK1/CR1/VSIG4/CCL2/CCL19/CCL21 | 8 |
| BP | GO:0090130 | tissue migration | 8/111 | 383/18888 | 0.001910863 | 0.023787932 | 0.017716381 | S100A12/S100A9/PTGS2/EGF/APOH/PLCG2/PTN/SERPINF1 | 8 |
| BP | GO:0006958 | complement activation, classical pathway | 3/111 | 42/18888 | 0.001919275 | 0.023787932 | 0.017716381 | CR1/C7/C3 | 3 |
| BP | GO:0043410 | positive regulation of MAPK cascade | 9/111 | 475/18888 | 0.001956265 | 0.024130388 | 0.017971429 | S100A12/IGF1/CHI3L1/EGF/PLCG2/CCL2/CCL18/CCL19/CCL21 | 9 |
| BP | GO:0002697 | regulation of immune effector process | 8/111 | 385/18888 | 0.001973837 | 0.024231193 | 0.018046505 | S100A9/JUNB/PCK1/CR1/PLCG2/VSIG4/CCL19/C3 | 8 |
| BP | GO:0033273 | response to vitamin | 4/111 | 91/18888 | 0.002038781 | 0.024607567 | 0.018326815 | PTGS2/FABP1/CYP27B1/TNC | 4 |
| BP | GO:1900542 | regulation of purine nucleotide metabolic process | 4/111 | 91/18888 | 0.002038781 | 0.024607567 | 0.018326815 | PDK4/IGF1/ALDOB/FBP1 | 4 |
| BP | GO:0035296 | regulation of tube diameter | 5/111 | 152/18888 | 0.002042676 | 0.024607567 | 0.018326815 | PTGS2/RGS2/HBB/KNG1/MMP2 | 5 |
| BP | GO:0097746 | blood vessel diameter maintenance | 5/111 | 152/18888 | 0.002042676 | 0.024607567 | 0.018326815 | PTGS2/RGS2/HBB/KNG1/MMP2 | 5 |
| BP | GO:0141091 | transforming growth factor beta receptor superfamily signaling pathway | 8/111 | 388/18888 | 0.00207136 | 0.024837048 | 0.018497724 | FOS/EGR1/LOX/HPGD/MAGI2/FGF9/ASPN/COL1A2 | 8 |
| BP | GO:0035150 | regulation of tube size | 5/111 | 153/18888 | 0.002101954 | 0.0249059 | 0.018549002 | PTGS2/RGS2/HBB/KNG1/MMP2 | 5 |
| BP | GO:0006140 | regulation of nucleotide metabolic process | 4/111 | 92/18888 | 0.002121921 | 0.0249059 | 0.018549002 | PDK4/IGF1/ALDOB/FBP1 | 4 |
| BP | GO:0019319 | hexose biosynthetic process | 4/111 | 92/18888 | 0.002121921 | 0.0249059 | 0.018549002 | ALDOB/FBP1/PCK1/ATF3 | 4 |
| BP | GO:0050673 | epithelial cell proliferation | 9/111 | 482/18888 | 0.002159577 | 0.0249059 | 0.018549002 | ZFP36/IGF1/EGF/APOH/DACH1/FGF9/PTN/SERPINF1/CCL2 | 9 |
| BP | GO:0051345 | positive regulation of hydrolase activity | 9/111 | 482/18888 | 0.002159577 | 0.0249059 | 0.018549002 | S100A9/S100A8/APOH/CR1/MAGI2/CCL2/CCL18/CCL19/CCL21 | 9 |
| BP | GO:0019682 | glyceraldehyde-3-phosphate metabolic process | 2/111 | 12/18888 | 0.002173711 | 0.0249059 | 0.018549002 | ALDOB/PCK1 | 2 |
| BP | GO:0072017 | distal tubule development | 2/111 | 12/18888 | 0.002173711 | 0.0249059 | 0.018549002 | UMOD/CALB1 | 2 |
| BP | GO:0072310 | glomerular epithelial cell development | 2/111 | 12/18888 | 0.002173711 | 0.0249059 | 0.018549002 | NPHS1/MAGI2 | 2 |
| BP | GO:0097048 | dendritic cell apoptotic process | 2/111 | 12/18888 | 0.002173711 | 0.0249059 | 0.018549002 | CCL19/CCL21 | 2 |
| BP | GO:2000668 | regulation of dendritic cell apoptotic process | 2/111 | 12/18888 | 0.002173711 | 0.0249059 | 0.018549002 | CCL19/CCL21 | 2 |
| BP | GO:0071604 | transforming growth factor beta production | 3/111 | 44/18888 | 0.002195422 | 0.024958832 | 0.018588424 | PTGS2/LUM/FN1 | 3 |
| BP | GO:0002690 | positive regulation of leukocyte chemotaxis | 4/111 | 93/18888 | 0.002207375 | 0.024958832 | 0.018588424 | CXCR2/PTN/CCL19/CCL21 | 4 |
| BP | GO:1904705 | regulation of vascular associated smooth muscle cell proliferation | 4/111 | 93/18888 | 0.002207375 | 0.024958832 | 0.018588424 | IGF1/HPGD/FGF9/MMP2 | 4 |
| BP | GO:0050920 | regulation of chemotaxis | 6/111 | 229/18888 | 0.0023386 | 0.026227477 | 0.019533264 | DUSP1/CXCR2/PTN/CCL2/CCL19/CCL21 | 6 |
| BP | GO:2000146 | negative regulation of cell motility | 8/111 | 396/18888 | 0.002350096 | 0.026227477 | 0.019533264 | DUSP1/APOH/DACH1/MAGI2/APOD/SERPINF1/CCL2/CCL21 | 8 |
| BP | GO:2001233 | regulation of apoptotic signaling pathway | 8/111 | 396/18888 | 0.002350096 | 0.026227477 | 0.019533264 | S100A9/PTGS2/S100A8/IGF1/NR4A2/G0S2/ATF3/MMP2 | 8 |
| BP | GO:1990874 | vascular associated smooth muscle cell proliferation | 4/111 | 95/18888 | 0.002385353 | 0.026506209 | 0.019740854 | IGF1/HPGD/FGF9/MMP2 | 4 |
| BP | GO:0006979 | response to oxidative stress | 8/111 | 398/18888 | 0.002424187 | 0.026822118 | 0.019976132 | FOS/PTGS2/HBB/FABP1/NR4A2/APOD/MMP2/FBLN5 | 8 |
| BP | GO:0046364 | monosaccharide biosynthetic process | 4/111 | 96/18888 | 0.002477942 | 0.027038266 | 0.02013711 | ALDOB/FBP1/PCK1/ATF3 | 4 |
| BP | GO:0002920 | regulation of humoral immune response | 3/111 | 46/18888 | 0.002495033 | 0.027038266 | 0.02013711 | CR1/VSIG4/C3 | 3 |
| BP | GO:1902622 | regulation of neutrophil migration | 3/111 | 46/18888 | 0.002495033 | 0.027038266 | 0.02013711 | CXCR2/CCL19/CCL21 | 3 |
| BP | GO:0001666 | response to hypoxia | 7/111 | 313/18888 | 0.002508384 | 0.027038266 | 0.02013711 | FOS/EGR1/PTGS2/PCK1/FABP1/NR4A2/MMP2 | 7 |
| BP | GO:0006971 | hypotonic response | 2/111 | 13/18888 | 0.002559091 | 0.027038266 | 0.02013711 | FBP1/PCK1 | 2 |
| BP | GO:0045916 | negative regulation of complement activation | 2/111 | 13/18888 | 0.002559091 | 0.027038266 | 0.02013711 | CR1/VSIG4 | 2 |
| BP | GO:0045986 | negative regulation of smooth muscle contraction | 2/111 | 13/18888 | 0.002559091 | 0.027038266 | 0.02013711 | PTGS2/RGS2 | 2 |
| BP | GO:0060100 | positive regulation of phagocytosis, engulfment | 2/111 | 13/18888 | 0.002559091 | 0.027038266 | 0.02013711 | PLCG2/C3 | 2 |
| BP | GO:0070294 | renal sodium ion absorption | 2/111 | 13/18888 | 0.002559091 | 0.027038266 | 0.02013711 | UMOD/SLC12A3 | 2 |
| BP | GO:0070486 | leukocyte aggregation | 2/111 | 13/18888 | 0.002559091 | 0.027038266 | 0.02013711 | S100A9/S100A8 | 2 |
| BP | GO:1905155 | positive regulation of membrane invagination | 2/111 | 13/18888 | 0.002559091 | 0.027038266 | 0.02013711 | PLCG2/C3 | 2 |
| BP | GO:0010976 | positive regulation of neuron projection development | 5/111 | 161/18888 | 0.002622361 | 0.027593665 | 0.020550751 | S100A9/RGS2/MAGI2/PTN/SERPINF1 | 5 |
| BP | GO:0055078 | sodium ion homeostasis | 3/111 | 47/18888 | 0.002653841 | 0.027811392 | 0.020712906 | UMOD/SLC12A3/CYP4A11 | 3 |
| BP | GO:0001667 | ameboidal-type cell migration | 9/111 | 498/18888 | 0.00268795 | 0.0280548 | 0.020894188 | S100A12/S100A9/PTGS2/EGF/APOH/PLCG2/PTN/SERPINF1/FN1 | 9 |
| BP | GO:0019217 | regulation of fatty acid metabolic process | 4/111 | 99/18888 | 0.00277049 | 0.028788827 | 0.021440864 | PDK4/PTGS2/FABP1/APOC1 | 4 |
| BP | GO:0050678 | regulation of epithelial cell proliferation | 8/111 | 407/18888 | 0.002780612 | 0.028788827 | 0.021440864 | ZFP36/IGF1/EGF/APOH/FGF9/PTN/SERPINF1/CCL2 | 8 |
| BP | GO:0031670 | cellular response to nutrient | 3/111 | 48/18888 | 0.002818757 | 0.029067022 | 0.021648054 | LPL/CYP27B1/TNC | 3 |
| BP | GO:0006000 | fructose metabolic process | 2/111 | 14/18888 | 0.002974175 | 0.030127035 | 0.022437513 | ALDOB/FBP1 | 2 |
| BP | GO:0042762 | regulation of sulfur metabolic process | 2/111 | 14/18888 | 0.002974175 | 0.030127035 | 0.022437513 | PDK4/BHMT | 2 |
| BP | GO:0051004 | regulation of lipoprotein lipase activity | 2/111 | 14/18888 | 0.002974175 | 0.030127035 | 0.022437513 | APOH/APOC1 | 2 |
| BP | GO:0071472 | cellular response to salt stress | 2/111 | 14/18888 | 0.002974175 | 0.030127035 | 0.022437513 | FBP1/PCK1 | 2 |
| BP | GO:0030850 | prostate gland development | 3/111 | 49/18888 | 0.002989857 | 0.030127035 | 0.022437513 | MMP2/TNC/SERPINF1 | 3 |
| BP | GO:0006633 | fatty acid biosynthetic process | 5/111 | 166/18888 | 0.002991668 | 0.030127035 | 0.022437513 | PDK4/PTGS2/LPL/PTGDS/APOC1 | 5 |
| BP | GO:0002274 | myeloid leukocyte activation | 6/111 | 241/18888 | 0.003012771 | 0.030221488 | 0.022507858 | S100A12/CXCR2/PTGDS/PLCG2/VSIG4/FN1 | 6 |
| BP | GO:0006937 | regulation of muscle contraction | 5/111 | 167/18888 | 0.003069814 | 0.030674341 | 0.022845126 | PTGS2/RGS2/EHD3/TNNI1/TNNT2 | 5 |
| BP | GO:0071827 | plasma lipoprotein particle organization | 3/111 | 50/18888 | 0.003167215 | 0.031525402 | 0.023478965 | LPL/APOM/APOC1 | 3 |
| BP | GO:0036293 | response to decreased oxygen levels | 7/111 | 327/18888 | 0.003196755 | 0.031697056 | 0.023606806 | FOS/EGR1/PTGS2/PCK1/FABP1/NR4A2/MMP2 | 7 |
| BP | GO:0007178 | transmembrane receptor protein serine/threonine kinase signaling pathway | 8/111 | 417/18888 | 0.003223428 | 0.031787264 | 0.02367399 | FOS/EGR1/LOX/HPGD/MAGI2/FGF9/ASPN/COL1A2 | 8 |
| BP | GO:0007204 | positive regulation of cytosolic calcium ion concentration | 5/111 | 169/18888 | 0.003230513 | 0.031787264 | 0.02367399 | CXCR2/KNG1/CXCR1/FPR1/FPR3 | 5 |
| BP | GO:0090257 | regulation of muscle system process | 6/111 | 245/18888 | 0.00326694 | 0.031937565 | 0.023785929 | PTGS2/RGS2/EHD3/TNNI1/IGF1/TNNT2 | 6 |
| BP | GO:0001558 | regulation of cell growth | 8/111 | 418/18888 | 0.003270565 | 0.031937565 | 0.023785929 | S100A9/RGS2/S100A8/IGF1/FBP1/CYP27B1/TNC/FN1 | 8 |
| BP | GO:0007519 | skeletal muscle tissue development | 5/111 | 170/18888 | 0.003313098 | 0.032164848 | 0.023955201 | FOS/EGR1/NPHS1/TCF21/ATF3 | 5 |
| BP | GO:0071622 | regulation of granulocyte chemotaxis | 3/111 | 51/18888 | 0.003350903 | 0.032164848 | 0.023955201 | CXCR2/CCL19/CCL21 | 3 |
| BP | GO:0001768 | establishment of T cell polarity | 2/111 | 15/18888 | 0.003418607 | 0.032164848 | 0.023955201 | CCL19/CCL21 | 2 |
| BP | GO:0007567 | parturition | 2/111 | 15/18888 | 0.003418607 | 0.032164848 | 0.023955201 | HPGD/MMP2 | 2 |
| BP | GO:0044849 | estrous cycle | 2/111 | 15/18888 | 0.003418607 | 0.032164848 | 0.023955201 | EGR1/PTN | 2 |
| BP | GO:0060099 | regulation of phagocytosis, engulfment | 2/111 | 15/18888 | 0.003418607 | 0.032164848 | 0.023955201 | PLCG2/C3 | 2 |
| BP | GO:0060192 | negative regulation of lipase activity | 2/111 | 15/18888 | 0.003418607 | 0.032164848 | 0.023955201 | RGS2/APOC1 | 2 |
| BP | GO:0072044 | collecting duct development | 2/111 | 15/18888 | 0.003418607 | 0.032164848 | 0.023955201 | UMOD/CALB1 | 2 |
| BP | GO:1905153 | regulation of membrane invagination | 2/111 | 15/18888 | 0.003418607 | 0.032164848 | 0.023955201 | PLCG2/C3 | 2 |
| BP | GO:2000402 | negative regulation of lymphocyte migration | 2/111 | 15/18888 | 0.003418607 | 0.032164848 | 0.023955201 | APOD/CCL2 | 2 |
| BP | GO:0009267 | cellular response to starvation | 5/111 | 172/18888 | 0.003482818 | 0.032649832 | 0.024316399 | FOS/ALB/PDK4/PCK1/ATF3 | 5 |
| BP | GO:0071470 | cellular response to osmotic stress | 3/111 | 52/18888 | 0.003540993 | 0.032741436 | 0.024384623 | PTGS2/FBP1/PCK1 | 3 |
| BP | GO:0009062 | fatty acid catabolic process | 4/111 | 106/18888 | 0.00354339 | 0.032741436 | 0.024384623 | HAO2/PCK1/FABP1/CYP4A11 | 4 |
| BP | GO:0032526 | response to retinoic acid | 4/111 | 106/18888 | 0.00354339 | 0.032741436 | 0.024384623 | PCK1/MMP2/TNC/SERPINF1 | 4 |
| BP | GO:0097306 | cellular response to alcohol | 4/111 | 106/18888 | 0.00354339 | 0.032741436 | 0.024384623 | FOS/FBP1/PCK1/TNC | 4 |
| BP | GO:1903706 | regulation of hemopoiesis | 8/111 | 424/18888 | 0.003564778 | 0.032821418 | 0.02444419 | FOS/ZFP36/LOX/JUNB/PCK1/CR1/PTN/CCL19 | 8 |
| BP | GO:0046394 | carboxylic acid biosynthetic process | 7/111 | 334/18888 | 0.003590999 | 0.032945178 | 0.024536362 | PDK4/PTGS2/LPL/PTGDS/BHMT/CYP4A11/APOC1 | 7 |
| BP | GO:0032963 | collagen metabolic process | 4/111 | 107/18888 | 0.00366458 | 0.033501017 | 0.024950331 | MFAP4/MMP2/COL1A2/MMP7 | 4 |
| BP | GO:0071825 | protein-lipid complex organization | 3/111 | 53/18888 | 0.003737553 | 0.034047393 | 0.025357252 | LPL/APOM/APOC1 | 3 |
| BP | GO:0051235 | maintenance of location | 7/111 | 337/18888 | 0.003770881 | 0.034230043 | 0.025493283 | S100A9/LPL/S100A8/PLCG2/CCL19/CCL21/C3 | 7 |
| BP | GO:0050870 | positive regulation of T cell activation | 6/111 | 253/18888 | 0.003822917 | 0.034361859 | 0.025591455 | IGF1/PCK1/CR1/CCL2/CCL19/CCL21 | 6 |
| BP | GO:0001767 | establishment of lymphocyte polarity | 2/111 | 16/18888 | 0.003892034 | 0.034361859 | 0.025591455 | CCL19/CCL21 | 2 |
| BP | GO:0001771 | immunological synapse formation | 2/111 | 16/18888 | 0.003892034 | 0.034361859 | 0.025591455 | CCL19/CCL21 | 2 |
| BP | GO:0002468 | dendritic cell antigen processing and presentation | 2/111 | 16/18888 | 0.003892034 | 0.034361859 | 0.025591455 | CCL19/CCL21 | 2 |
| BP | GO:0003096 | renal sodium ion transport | 2/111 | 16/18888 | 0.003892034 | 0.034361859 | 0.025591455 | UMOD/SLC12A3 | 2 |
| BP | GO:0017014 | protein nitrosylation | 2/111 | 16/18888 | 0.003892034 | 0.034361859 | 0.025591455 | S100A9/S100A8 | 2 |
| BP | GO:0018119 | peptidyl-cysteine S-nitrosylation | 2/111 | 16/18888 | 0.003892034 | 0.034361859 | 0.025591455 | S100A9/S100A8 | 2 |
| BP | GO:0030728 | ovulation | 2/111 | 16/18888 | 0.003892034 | 0.034361859 | 0.025591455 | HPGD/MMP2 | 2 |
| BP | GO:0040013 | negative regulation of locomotion | 8/111 | 431/18888 | 0.003933585 | 0.034437276 | 0.025647622 | DUSP1/APOH/DACH1/MAGI2/APOD/SERPINF1/CCL2/CCL21 | 8 |
| BP | GO:0042304 | regulation of fatty acid biosynthetic process | 3/111 | 54/18888 | 0.00394065 | 0.034437276 | 0.025647622 | PDK4/PTGS2/APOC1 | 3 |
| BP | GO:0071385 | cellular response to glucocorticoid stimulus | 3/111 | 54/18888 | 0.00394065 | 0.034437276 | 0.025647622 | ZFP36/PCK1/SERPINF1 | 3 |
| BP | GO:0032611 | interleukin-1 beta production | 4/111 | 110/18888 | 0.004044981 | 0.03511098 | 0.026149372 | EGR1/LPL/IGF1/CCL19 | 4 |
| BP | GO:0032651 | regulation of interleukin-1 beta production | 4/111 | 110/18888 | 0.004044981 | 0.03511098 | 0.026149372 | EGR1/LPL/IGF1/CCL19 | 4 |
| BP | GO:0001649 | osteoblast differentiation | 6/111 | 257/18888 | 0.004125894 | 0.03569314 | 0.026582943 | LOX/JUNB/IGF1/FGF9/TNC/VCAN | 6 |
| BP | GO:0010559 | regulation of glycoprotein biosynthetic process | 3/111 | 55/18888 | 0.004150349 | 0.035784617 | 0.026651072 | IGF1/CCL19/CCL21 | 3 |
| BP | GO:0002455 | humoral immune response mediated by circulating immunoglobulin | 3/111 | 56/18888 | 0.004366714 | 0.03725761 | 0.027748104 | CR1/C7/C3 | 3 |
| BP | GO:0002921 | negative regulation of humoral immune response | 2/111 | 17/18888 | 0.004394106 | 0.03725761 | 0.027748104 | CR1/VSIG4 | 2 |
| BP | GO:0034392 | negative regulation of smooth muscle cell apoptotic process | 2/111 | 17/18888 | 0.004394106 | 0.03725761 | 0.027748104 | IGF1/APOH | 2 |
| BP | GO:0051412 | response to corticosterone | 2/111 | 17/18888 | 0.004394106 | 0.03725761 | 0.027748104 | FOS/FOSB | 2 |
| BP | GO:0072224 | metanephric glomerulus development | 2/111 | 17/18888 | 0.004394106 | 0.03725761 | 0.027748104 | EGR1/TCF21 | 2 |
| BP | GO:0006109 | regulation of carbohydrate metabolic process | 5/111 | 182/18888 | 0.004426425 | 0.03725761 | 0.027748104 | PDK4/IGF1/EGF/ALDOB/FBP1 | 5 |
| BP | GO:0060538 | skeletal muscle organ development | 5/111 | 182/18888 | 0.004426425 | 0.03725761 | 0.027748104 | FOS/EGR1/NPHS1/TCF21/ATF3 | 5 |
| BP | GO:0034440 | lipid oxidation | 4/111 | 113/18888 | 0.004451258 | 0.03725761 | 0.027748104 | PDK4/HAO2/FABP1/APOD | 4 |
| BP | GO:0062014 | negative regulation of small molecule metabolic process | 4/111 | 113/18888 | 0.004451258 | 0.03725761 | 0.027748104 | ALDOB/FBP1/CYP27B1/APOC1 | 4 |
| BP | GO:0070542 | response to fatty acid | 3/111 | 57/18888 | 0.004589805 | 0.038046676 | 0.028335771 | PDK4/PTGS2/LPL | 3 |
| BP | GO:1901616 | organic hydroxy compound catabolic process | 3/111 | 57/18888 | 0.004589805 | 0.038046676 | 0.028335771 | CYP27B1/CYP4A11/MOXD1 | 3 |
| BP | GO:2000107 | negative regulation of leukocyte apoptotic process | 3/111 | 57/18888 | 0.004589805 | 0.038046676 | 0.028335771 | CXCR2/CCL19/CCL21 | 3 |
| BP | GO:0014706 | striated muscle tissue development | 6/111 | 265/18888 | 0.004784773 | 0.039535718 | 0.029444755 | LOX/RGS2/TNNI1/IGF1/TNNT2/FGF9 | 6 |
| BP | GO:0061005 | cell differentiation involved in kidney development | 3/111 | 58/18888 | 0.004819682 | 0.039696933 | 0.029564822 | NPHS1/TCF21/MAGI2 | 3 |
| BP | GO:1905952 | regulation of lipid localization | 5/111 | 186/18888 | 0.004850515 | 0.039823651 | 0.029659197 | LPL/EGF/CYP4A11/APOC1/C3 | 5 |
| BP | GO:0070482 | response to oxygen levels | 7/111 | 354/18888 | 0.004923183 | 0.039922324 | 0.029732685 | FOS/EGR1/PTGS2/PCK1/FABP1/NR4A2/MMP2 | 7 |
| BP | GO:0002524 | hypersensitivity | 2/111 | 18/18888 | 0.004924476 | 0.039922324 | 0.029732685 | FCGR3B/C3 | 2 |
| BP | GO:0019373 | epoxygenase P450 pathway | 2/111 | 18/18888 | 0.004924476 | 0.039922324 | 0.029732685 | CYP2B6/CYP4A11 | 2 |
| BP | GO:1901569 | fatty acid derivative catabolic process | 2/111 | 18/18888 | 0.004924476 | 0.039922324 | 0.029732685 | HPGD/CYP4A11 | 2 |
| BP | GO:0071900 | regulation of protein serine/threonine kinase activity | 6/111 | 267/18888 | 0.004960949 | 0.040091933 | 0.029859004 | S100A12/RGS2/DUSP1/PRKAR2B/EGF/CCL19 | 6 |
| BP | GO:0007566 | embryo implantation | 3/111 | 59/18888 | 0.005056404 | 0.040735652 | 0.030338422 | PTGS2/TPPP3/MMP2 | 3 |
| BP | GO:0032640 | tumor necrosis factor production | 5/111 | 189/18888 | 0.005186988 | 0.041407882 | 0.030839074 | ZFP36/LPL/IGF1/PLCG2/CCL19 | 5 |
| BP | GO:0032680 | regulation of tumor necrosis factor production | 5/111 | 189/18888 | 0.005186988 | 0.041407882 | 0.030839074 | ZFP36/LPL/IGF1/PLCG2/CCL19 | 5 |
| BP | GO:0009308 | amine metabolic process | 4/111 | 118/18888 | 0.005188032 | 0.041407882 | 0.030839074 | AGMAT/NR4A2/BHMT/MOXD1 | 4 |
| BP | GO:0001706 | endoderm formation | 3/111 | 60/18888 | 0.005300026 | 0.041912476 | 0.031214877 | DUSP1/MMP2/FN1 | 3 |
| BP | GO:0046173 | polyol biosynthetic process | 3/111 | 60/18888 | 0.005300026 | 0.041912476 | 0.031214877 | PCK1/CYP27B1/PLCG2 | 3 |
| BP | GO:0046456 | icosanoid biosynthetic process | 3/111 | 60/18888 | 0.005300026 | 0.041912476 | 0.031214877 | PTGS2/PTGDS/CYP4A11 | 3 |
| BP | GO:0071695 | anatomical structure maturation | 6/111 | 271/18888 | 0.005327568 | 0.042001442 | 0.031281136 | IGF1/NR4A2/MMP2/CCL19/CCL21/C3 | 6 |
| BP | GO:0010720 | positive regulation of cell development | 8/111 | 454/18888 | 0.005356767 | 0.042102881 | 0.031356684 | FOS/PCK1/CR1/PTN/SERPINF1/CCL19/VCAN/FN1 | 8 |
| BP | GO:0002544 | chronic inflammatory response | 2/111 | 19/18888 | 0.005482801 | 0.042684508 | 0.031789858 | S100A9/S100A8 | 2 |
| BP | GO:0055064 | chloride ion homeostasis | 2/111 | 19/18888 | 0.005482801 | 0.042684508 | 0.031789858 | UMOD/SLC12A3 | 2 |
| BP | GO:0006869 | lipid transport | 8/111 | 456/18888 | 0.005496996 | 0.042684508 | 0.031789858 | PTGS2/EGF/APOH/FABP1/APOD/APOM/CYP4A11/APOC1 | 8 |
| BP | GO:0032102 | negative regulation of response to external stimulus | 8/111 | 456/18888 | 0.005496996 | 0.042684508 | 0.031789858 | ZFP36/DUSP1/IGF1/KNG1/APOH/CR1/VSIG4/CCL2 | 8 |
| BP | GO:0043470 | regulation of carbohydrate catabolic process | 3/111 | 61/18888 | 0.005550603 | 0.042842682 | 0.031907661 | IGF1/ALDOB/FBP1 | 3 |
| BP | GO:1903018 | regulation of glycoprotein metabolic process | 3/111 | 61/18888 | 0.005550603 | 0.042842682 | 0.031907661 | IGF1/CCL19/CCL21 | 3 |
| BP | GO:0030324 | lung development | 5/111 | 193/18888 | 0.005660943 | 0.04356391 | 0.032444805 | LOX/CHI3L1/TCF21/FGF9/TNC | 5 |
| BP | GO:0003018 | vascular process in circulatory system | 6/111 | 275/18888 | 0.005713704 | 0.043839074 | 0.032649737 | PTGS2/RGS2/HBB/CXCR2/KNG1/MMP2 | 6 |
| BP | GO:0071466 | cellular response to xenobiotic stimulus | 5/111 | 194/18888 | 0.005784054 | 0.04391059 | 0.032702999 | S100A12/GSTA1/GLYAT/FBP1/CYP2B6 | 5 |
| BP | GO:0071706 | tumor necrosis factor superfamily cytokine production | 5/111 | 194/18888 | 0.005784054 | 0.04391059 | 0.032702999 | ZFP36/LPL/IGF1/PLCG2/CCL19 | 5 |
| BP | GO:1903555 | regulation of tumor necrosis factor superfamily cytokine production | 5/111 | 194/18888 | 0.005784054 | 0.04391059 | 0.032702999 | ZFP36/LPL/IGF1/PLCG2/CCL19 | 5 |
| BP | GO:0002090 | regulation of receptor internalization | 3/111 | 62/18888 | 0.005808189 | 0.04391059 | 0.032702999 | EGF/MAGI2/PLCG2 | 3 |
| BP | GO:0032731 | positive regulation of interleukin-1 beta production | 3/111 | 62/18888 | 0.005808189 | 0.04391059 | 0.032702999 | EGR1/LPL/CCL19 | 3 |
| BP | GO:1903039 | positive regulation of leukocyte cell-cell adhesion | 6/111 | 277/18888 | 0.005914276 | 0.044581878 | 0.03320295 | IGF1/PCK1/CR1/CCL2/CCL19/CCL21 | 6 |
| BP | GO:0071675 | regulation of mononuclear cell migration | 4/111 | 123/18888 | 0.006002432 | 0.045088542 | 0.033580295 | DUSP1/APOD/CCL2/CCL21 | 4 |
| BP | GO:0007611 | learning or memory | 6/111 | 278/18888 | 0.00601647 | 0.045088542 | 0.033580295 | FOS/PTGS2/PRKAR2B/CALB1/PTN/SERPINF1 | 6 |
| BP | GO:0002577 | regulation of antigen processing and presentation | 2/111 | 20/18888 | 0.006068739 | 0.045217366 | 0.033676239 | CCL19/CCL21 | 2 |
| BP | GO:0071474 | cellular hyperosmotic response | 2/111 | 20/18888 | 0.006068739 | 0.045217366 | 0.033676239 | FBP1/PCK1 | 2 |
| BP | GO:0030323 | respiratory tube development | 5/111 | 197/18888 | 0.006164721 | 0.045482773 | 0.033873904 | LOX/CHI3L1/TCF21/FGF9/TNC | 5 |
| BP | GO:0010951 | negative regulation of endopeptidase activity | 4/111 | 124/18888 | 0.00617493 | 0.045482773 | 0.033873904 | KNG1/FABP1/CR1/SERPINF1 | 4 |
| BP | GO:0043200 | response to amino acid | 4/111 | 124/18888 | 0.00617493 | 0.045482773 | 0.033873904 | SST/PCK1/MMP2/COL1A2 | 4 |
| BP | GO:0071774 | response to fibroblast growth factor | 4/111 | 124/18888 | 0.00617493 | 0.045482773 | 0.033873904 | ZFP36/FGF9/TNC/CCL2 | 4 |
| BP | GO:0052548 | regulation of endopeptidase activity | 6/111 | 280/18888 | 0.006224721 | 0.045718888 | 0.034049754 | S100A9/S100A8/KNG1/FABP1/CR1/SERPINF1 | 6 |
| BP | GO:0071384 | cellular response to corticosteroid stimulus | 3/111 | 64/18888 | 0.006344587 | 0.046335257 | 0.034508802 | ZFP36/PCK1/SERPINF1 | 3 |
| BP | GO:2001244 | positive regulation of intrinsic apoptotic signaling pathway | 3/111 | 64/18888 | 0.006344587 | 0.046335257 | 0.034508802 | S100A9/S100A8/MMP2 | 3 |
| BP | GO:0006066 | alcohol metabolic process | 7/111 | 373/18888 | 0.006505781 | 0.047258783 | 0.035196611 | IGF1/PCK1/CYP27B1/PLCG2/APOC1/ADH1B/MOXD1 | 7 |
| BP | GO:0006805 | xenobiotic metabolic process | 4/111 | 126/18888 | 0.00652976 | 0.047258783 | 0.035196611 | S100A12/GSTA1/GLYAT/CYP2B6 | 4 |
| BP | GO:0007613 | memory | 4/111 | 126/18888 | 0.00652976 | 0.047258783 | 0.035196611 | PTGS2/CALB1/PTN/SERPINF1 | 4 |
| BP | GO:0045859 | regulation of protein kinase activity | 8/111 | 470/18888 | 0.006558852 | 0.047258783 | 0.035196611 | S100A12/RGS2/DUSP1/IGF1/CHI3L1/PRKAR2B/EGF/CCL19 | 8 |
| BP | GO:0031099 | regeneration | 5/111 | 200/18888 | 0.006562701 | 0.047258783 | 0.035196611 | IGF1/APOD/MMP2/TNC/PTN | 5 |
| BP | GO:0033033 | negative regulation of myeloid cell apoptotic process | 2/111 | 21/18888 | 0.006681953 | 0.047454751 | 0.035342561 | CXCR2/APOH | 2 |
| BP | GO:0043651 | linoleic acid metabolic process | 2/111 | 21/18888 | 0.006681953 | 0.047454751 | 0.035342561 | GSTA1/CYP4A11 | 2 |
| BP | GO:0055081 | monoatomic anion homeostasis | 2/111 | 21/18888 | 0.006681953 | 0.047454751 | 0.035342561 | UMOD/SLC12A3 | 2 |
| BP | GO:0061050 | regulation of cell growth involved in cardiac muscle cell development | 2/111 | 21/18888 | 0.006681953 | 0.047454751 | 0.035342561 | RGS2/IGF1 | 2 |
| BP | GO:0071498 | cellular response to fluid shear stress | 2/111 | 21/18888 | 0.006681953 | 0.047454751 | 0.035342561 | PTGS2/MMP2 | 2 |
| BP | GO:0003014 | renal system process | 4/111 | 127/18888 | 0.006712144 | 0.047538203 | 0.035404713 | UMOD/SLC12A3/HBB/CYP4A11 | 4 |
| BP | GO:0001655 | urogenital system development | 3/111 | 66/18888 | 0.006909611 | 0.048669336 | 0.036247139 | MMP2/TNC/SERPINF1 | 3 |
| BP | GO:0050994 | regulation of lipid catabolic process | 3/111 | 66/18888 | 0.006909611 | 0.048669336 | 0.036247139 | HPGD/FABP1/APOC1 | 3 |
| BP | GO:0009749 | response to glucose | 5/111 | 203/18888 | 0.006978394 | 0.04901989 | 0.036508219 | EGR1/LPL/PCK1/SERPINF1/COL6A3 | 5 |
| BP | GO:0032612 | interleukin-1 production | 4/111 | 129/18888 | 0.007086978 | 0.049482051 | 0.03685242 | EGR1/LPL/IGF1/CCL19 | 4 |
| BP | GO:0032652 | regulation of interleukin-1 production | 4/111 | 129/18888 | 0.007086978 | 0.049482051 | 0.03685242 | EGR1/LPL/IGF1/CCL19 | 4 |
| BP | GO:0061448 | connective tissue development | 6/111 | 288/18888 | 0.007110581 | 0.049482051 | 0.03685242 | EGR1/LOX/LPL/CHI3L1/FGF9/TGFBI | 6 |
| BP | GO:0055001 | muscle cell development | 5/111 | 204/18888 | 0.007120962 | 0.049482051 | 0.03685242 | LOX/RGS2/IGF1/TNNT2/KRT19 | 5 |
| BP | GO:0055021 | regulation of cardiac muscle tissue growth | 3/111 | 67/18888 | 0.007202972 | 0.049805788 | 0.037093527 | RGS2/IGF1/FGF9 | 3 |
| BP | GO:0007530 | sex determination | 2/111 | 22/18888 | 0.007322108 | 0.049805788 | 0.037093527 | TCF21/FGF9 | 2 |
| BP | GO:0009713 | catechol-containing compound biosynthetic process | 2/111 | 22/18888 | 0.007322108 | 0.049805788 | 0.037093527 | NR4A2/MOXD1 | 2 |
| BP | GO:0042423 | catecholamine biosynthetic process | 2/111 | 22/18888 | 0.007322108 | 0.049805788 | 0.037093527 | NR4A2/MOXD1 | 2 |
| BP | GO:0061318 | renal filtration cell differentiation | 2/111 | 22/18888 | 0.007322108 | 0.049805788 | 0.037093527 | NPHS1/MAGI2 | 2 |
| BP | GO:0071305 | cellular response to vitamin D | 2/111 | 22/18888 | 0.007322108 | 0.049805788 | 0.037093527 | CYP27B1/TNC | 2 |
| BP | GO:0072112 | podocyte differentiation | 2/111 | 22/18888 | 0.007322108 | 0.049805788 | 0.037093527 | NPHS1/MAGI2 | 2 |
| BP | GO:0072234 | metanephric nephron tubule development | 2/111 | 22/18888 | 0.007322108 | 0.049805788 | 0.037093527 | UMOD/CALB1 | 2 |
| BP | GO:1903037 | regulation of leukocyte cell-cell adhesion | 7/111 | 382/18888 | 0.00737575 | 0.050038639 | 0.037266946 | IGF1/PCK1/CR1/VSIG4/CCL2/CCL19/CCL21 | 7 |
| BP | GO:0071300 | cellular response to retinoic acid | 3/111 | 68/18888 | 0.007503625 | 0.050772561 | 0.037813545 | PCK1/TNC/SERPINF1 | 3 |
| BP | GO:0010466 | negative regulation of peptidase activity | 4/111 | 132/18888 | 0.007674778 | 0.051722156 | 0.038520768 | KNG1/FABP1/CR1/SERPINF1 | 4 |
| BP | GO:0002460 | adaptive immune response based on somatic recombination of immune receptors built from immunoglobulin superfamily domains | 7/111 | 385/18888 | 0.007684091 | 0.051722156 | 0.038520768 | JUNB/CR1/FCGR3B/IGKC/CCL19/C7/C3 | 7 |
| BP | GO:0016064 | immunoglobulin mediated immune response | 5/111 | 208/18888 | 0.007711644 | 0.051772443 | 0.03855822 | CR1/FCGR3B/IGKC/C7/C3 | 5 |
| BP | GO:0060191 | regulation of lipase activity | 3/111 | 69/18888 | 0.007811611 | 0.05230736 | 0.038956606 | RGS2/APOH/APOC1 | 3 |
| BP | GO:0002689 | negative regulation of leukocyte chemotaxis | 2/111 | 23/18888 | 0.007988871 | 0.052944243 | 0.039430934 | DUSP1/CCL2 | 2 |
| BP | GO:0045932 | negative regulation of muscle contraction | 2/111 | 23/18888 | 0.007988871 | 0.052944243 | 0.039430934 | PTGS2/RGS2 | 2 |
| BP | GO:0071636 | positive regulation of transforming growth factor beta production | 2/111 | 23/18888 | 0.007988871 | 0.052944243 | 0.039430934 | PTGS2/LUM | 2 |
| BP | GO:0072311 | glomerular epithelial cell differentiation | 2/111 | 23/18888 | 0.007988871 | 0.052944243 | 0.039430934 | NPHS1/MAGI2 | 2 |
| BP | GO:0043627 | response to estrogen | 3/111 | 70/18888 | 0.00812697 | 0.053583964 | 0.039907374 | CYP27B1/KRT19/MMP2 | 3 |
| BP | GO:2000401 | regulation of lymphocyte migration | 3/111 | 70/18888 | 0.00812697 | 0.053583964 | 0.039907374 | APOD/CCL2/CCL21 | 3 |
| BP | GO:0002285 | lymphocyte activation involved in immune response | 5/111 | 212/18888 | 0.008335665 | 0.054585154 | 0.040653024 | JUNB/PCK1/CR1/PLCG2/CCL19 | 5 |
| BP | GO:0019724 | B cell mediated immunity | 5/111 | 212/18888 | 0.008335665 | 0.054585154 | 0.040653024 | CR1/FCGR3B/IGKC/C7/C3 | 5 |
| BP | GO:0010632 | regulation of epithelial cell migration | 6/111 | 298/18888 | 0.008342339 | 0.054585154 | 0.040653024 | PTGS2/EGF/APOH/PLCG2/PTN/SERPINF1 | 6 |
| BP | GO:0045600 | positive regulation of fat cell differentiation | 3/111 | 71/18888 | 0.008449742 | 0.055147936 | 0.041072163 | ZFP36/PTGS2/LPL | 3 |
| BP | GO:2000241 | regulation of reproductive process | 5/111 | 214/18888 | 0.008660462 | 0.055538404 | 0.04136297 | DUSP1/IGF1/PTGDS/FGF9/SERPINF1 | 5 |
| BP | GO:0002922 | positive regulation of humoral immune response | 2/111 | 24/18888 | 0.008681915 | 0.055538404 | 0.04136297 | CR1/C3 | 2 |
| BP | GO:0003091 | renal water homeostasis | 2/111 | 24/18888 | 0.008681915 | 0.055538404 | 0.04136297 | UMOD/CYP4A11 | 2 |
| BP | GO:0006706 | steroid catabolic process | 2/111 | 24/18888 | 0.008681915 | 0.055538404 | 0.04136297 | HSD17B14/CYP27B1 | 2 |
| BP | GO:0009651 | response to salt stress | 2/111 | 24/18888 | 0.008681915 | 0.055538404 | 0.04136297 | FBP1/PCK1 | 2 |
| BP | GO:0010640 | regulation of platelet-derived growth factor receptor signaling pathway | 2/111 | 24/18888 | 0.008681915 | 0.055538404 | 0.04136297 | LOX/APOD | 2 |
| BP | GO:0034695 | response to prostaglandin E | 2/111 | 24/18888 | 0.008681915 | 0.055538404 | 0.04136297 | CCL19/CCL21 | 2 |
| BP | GO:0046885 | regulation of hormone biosynthetic process | 2/111 | 24/18888 | 0.008681915 | 0.055538404 | 0.04136297 | EGR1/CYP27B1 | 2 |
| BP | GO:0002366 | leukocyte activation involved in immune response | 6/111 | 301/18888 | 0.00874007 | 0.055772029 | 0.041536965 | JUNB/PTGDS/PCK1/CR1/PLCG2/CCL19 | 6 |
| BP | GO:0050766 | positive regulation of phagocytosis | 3/111 | 72/18888 | 0.008779963 | 0.055888257 | 0.041623527 | PLCG2/CCL2/C3 | 3 |
| BP | GO:0016049 | cell growth | 8/111 | 496/18888 | 0.008936334 | 0.056743521 | 0.042260496 | S100A9/RGS2/S100A8/IGF1/FBP1/CYP27B1/TNC/FN1 | 8 |
| BP | GO:1901605 | alpha-amino acid metabolic process | 5/111 | 216/18888 | 0.008993931 | 0.056968928 | 0.042428372 | HPD/AGMAT/GLYAT/BHMT/PRODH2 | 5 |
| BP | GO:0032732 | positive regulation of interleukin-1 production | 3/111 | 73/18888 | 0.009117669 | 0.057470295 | 0.042801771 | EGR1/LPL/CCL19 | 3 |
| BP | GO:0060420 | regulation of heart growth | 3/111 | 73/18888 | 0.009117669 | 0.057470295 | 0.042801771 | RGS2/IGF1/FGF9 | 3 |
| BP | GO:0052547 | regulation of peptidase activity | 6/111 | 304/18888 | 0.00915125 | 0.057541272 | 0.042854632 | S100A9/S100A8/KNG1/FABP1/CR1/SERPINF1 | 6 |
| BP | GO:0002263 | cell activation involved in immune response | 6/111 | 305/18888 | 0.00929134 | 0.058279987 | 0.0434048 | JUNB/PTGDS/PCK1/CR1/PLCG2/CCL19 | 6 |
| BP | GO:0001819 | positive regulation of cytokine production | 8/111 | 500/18888 | 0.009352894 | 0.058381984 | 0.043480764 | EGR1/PTGS2/LPL/CHI3L1/PLCG2/CCL19/C3/LUM | 8 |
| BP | GO:0002757 | immune response-activating signaling pathway | 8/111 | 500/18888 | 0.009352894 | 0.058381984 | 0.043480764 | S100A9/S100A8/CR1/FPR1/PLCG2/IGKC/FPR3/C3 | 8 |
| BP | GO:0072243 | metanephric nephron epithelium development | 2/111 | 25/18888 | 0.009400912 | 0.058502022 | 0.043570164 | UMOD/CALB1 | 2 |
| BP | GO:0014823 | response to activity | 3/111 | 74/18888 | 0.009462895 | 0.058502022 | 0.043570164 | FOS/PCK1/MMP2 | 3 |
| BP | GO:0033344 | cholesterol efflux | 3/111 | 74/18888 | 0.009462895 | 0.058502022 | 0.043570164 | EGF/APOM/APOC1 | 3 |
| BP | GO:0072678 | T cell migration | 3/111 | 74/18888 | 0.009462895 | 0.058502022 | 0.043570164 | APOD/CCL2/CCL21 | 3 |
| BP | GO:0048608 | reproductive structure development | 6/111 | 307/18888 | 0.00957612 | 0.059060378 | 0.043986007 | TCF21/FGF9/MMP2/TNC/SERPINF1/C3 | 6 |
| BP | GO:0051092 | positive regulation of NF-kappaB transcription factor activity | 4/111 | 141/18888 | 0.009628275 | 0.059240315 | 0.044120018 | S100A12/S100A9/S100A8/PLCG2 | 4 |
| BP | GO:0060541 | respiratory system development | 5/111 | 220/18888 | 0.009687328 | 0.059461741 | 0.044284927 | LOX/CHI3L1/TCF21/FGF9/TNC | 5 |
| BP | GO:0046165 | alcohol biosynthetic process | 4/111 | 142/18888 | 0.009863439 | 0.060255795 | 0.04487631 | PCK1/CYP27B1/PLCG2/MOXD1 | 4 |
| BP | GO:0050921 | positive regulation of chemotaxis | 4/111 | 142/18888 | 0.009863439 | 0.060255795 | 0.04487631 | CXCR2/PTN/CCL19/CCL21 | 4 |
| BP | GO:0032925 | regulation of activin receptor signaling pathway | 2/111 | 26/18888 | 0.01014554 | 0.061151104 | 0.045543103 | MAGI2/FGF9 | 2 |
| BP | GO:0060740 | prostate gland epithelium morphogenesis | 2/111 | 26/18888 | 0.01014554 | 0.061151104 | 0.045543103 | MMP2/TNC | 2 |
| BP | GO:0072010 | glomerular epithelium development | 2/111 | 26/18888 | 0.01014554 | 0.061151104 | 0.045543103 | NPHS1/MAGI2 | 2 |
| BP | GO:1900543 | negative regulation of purine nucleotide metabolic process | 2/111 | 26/18888 | 0.01014554 | 0.061151104 | 0.045543103 | ALDOB/FBP1 | 2 |
| BP | GO:1901623 | regulation of lymphocyte chemotaxis | 2/111 | 26/18888 | 0.01014554 | 0.061151104 | 0.045543103 | CCL2/CCL21 | 2 |
| BP | GO:0061458 | reproductive system development | 6/111 | 311/18888 | 0.010164325 | 0.061151104 | 0.045543103 | TCF21/FGF9/MMP2/TNC/SERPINF1/C3 | 6 |
| BP | GO:0002224 | toll-like receptor signaling pathway | 3/111 | 76/18888 | 0.010176037 | 0.061151104 | 0.045543103 | S100A9/S100A8/PLCG2 | 3 |
| BP | GO:0035914 | skeletal muscle cell differentiation | 3/111 | 77/18888 | 0.010544015 | 0.063215049 | 0.047080254 | FOS/EGR1/ATF3 | 3 |
| BP | GO:0060048 | cardiac muscle contraction | 4/111 | 145/18888 | 0.010591244 | 0.063285135 | 0.047132451 | RGS2/EHD3/TNNI1/TNNT2 | 4 |
| BP | GO:0072330 | monocarboxylic acid biosynthetic process | 5/111 | 225/18888 | 0.010604801 | 0.063285135 | 0.047132451 | PDK4/PTGS2/LPL/PTGDS/APOC1 | 5 |
| BP | GO:0001906 | cell killing | 5/111 | 226/18888 | 0.010795193 | 0.063809753 | 0.047523167 | S100A12/CR1/FCGR3B/C7/C3 | 5 |
| BP | GO:0010447 | response to acidic pH | 2/111 | 27/18888 | 0.010915477 | 0.063809753 | 0.047523167 | SST/SERPINF1 | 2 |
| BP | GO:0010884 | positive regulation of lipid storage | 2/111 | 27/18888 | 0.010915477 | 0.063809753 | 0.047523167 | LPL/C3 | 2 |
| BP | GO:0019755 | one-carbon compound transport | 2/111 | 27/18888 | 0.010915477 | 0.063809753 | 0.047523167 | UMOD/HBB | 2 |
| BP | GO:0034377 | plasma lipoprotein particle assembly | 2/111 | 27/18888 | 0.010915477 | 0.063809753 | 0.047523167 | APOM/APOC1 | 2 |
| BP | GO:0045980 | negative regulation of nucleotide metabolic process | 2/111 | 27/18888 | 0.010915477 | 0.063809753 | 0.047523167 | ALDOB/FBP1 | 2 |
| BP | GO:0048245 | eosinophil chemotaxis | 2/111 | 27/18888 | 0.010915477 | 0.063809753 | 0.047523167 | CCL2/CCL18 | 2 |
| BP | GO:0071549 | cellular response to dexamethasone stimulus | 2/111 | 27/18888 | 0.010915477 | 0.063809753 | 0.047523167 | PCK1/SERPINF1 | 2 |
| BP | GO:0099560 | synaptic membrane adhesion | 2/111 | 27/18888 | 0.010915477 | 0.063809753 | 0.047523167 | CDH10/CDH6 | 2 |
| BP | GO:0170039 | proteinogenic amino acid metabolic process | 4/111 | 147/18888 | 0.011095253 | 0.064713941 | 0.048196574 | HPD/GLYAT/BHMT/PRODH2 | 4 |
| BP | GO:0051651 | maintenance of location in cell | 5/111 | 228/18888 | 0.011182978 | 0.065078369 | 0.048467986 | S100A9/S100A8/PLCG2/CCL19/CCL21 | 5 |
| BP | GO:0042246 | tissue regeneration | 3/111 | 79/18888 | 0.01130293 | 0.065628275 | 0.048877536 | IGF1/APOD/PTN | 3 |
| BP | GO:0002687 | positive regulation of leukocyte migration | 4/111 | 149/18888 | 0.011614501 | 0.067285805 | 0.050112004 | CXCR2/PTN/CCL19/CCL21 | 4 |
| BP | GO:0002675 | positive regulation of acute inflammatory response | 2/111 | 28/18888 | 0.011710407 | 0.067387121 | 0.05018746 | PTGS2/C3 | 2 |
| BP | GO:0010800 | positive regulation of peptidyl-threonine phosphorylation | 2/111 | 28/18888 | 0.011710407 | 0.067387121 | 0.05018746 | CHI3L1/EGF | 2 |
| BP | GO:0060512 | prostate gland morphogenesis | 2/111 | 28/18888 | 0.011710407 | 0.067387121 | 0.05018746 | MMP2/TNC | 2 |
| BP | GO:0050890 | cognition | 6/111 | 322/18888 | 0.011914151 | 0.068406865 | 0.050946928 | FOS/PTGS2/PRKAR2B/CALB1/PTN/SERPINF1 | 6 |
| BP | GO:0034614 | cellular response to reactive oxygen species | 4/111 | 151/18888 | 0.012149149 | 0.069601123 | 0.051836368 | FOS/FABP1/MMP2/FBLN5 | 4 |
| BP | GO:0010594 | regulation of endothelial cell migration | 5/111 | 234/18888 | 0.012403269 | 0.07068354 | 0.052642512 | PTGS2/EGF/APOH/PTN/SERPINF1 | 5 |
| BP | GO:0046434 | organophosphate catabolic process | 5/111 | 234/18888 | 0.012403269 | 0.07068354 | 0.052642512 | IGF1/ALDOB/FBP1/PLCG2/APOC1 | 5 |
| BP | GO:0022409 | positive regulation of cell-cell adhesion | 6/111 | 325/18888 | 0.012426129 | 0.07068354 | 0.052642512 | IGF1/PCK1/CR1/CCL2/CCL19/CCL21 | 6 |
| BP | GO:0043406 | positive regulation of MAP kinase activity | 3/111 | 82/18888 | 0.012499094 | 0.07068354 | 0.052642512 | S100A12/EGF/CCL19 | 3 |
| BP | GO:0061844 | antimicrobial humoral immune response mediated by antimicrobial peptide | 3/111 | 82/18888 | 0.012499094 | 0.07068354 | 0.052642512 | S100A12/S100A9/RNASE6 | 3 |
| BP | GO:0003401 | axis elongation | 2/111 | 29/18888 | 0.012530015 | 0.07068354 | 0.052642512 | MAGI2/TNC | 2 |
| BP | GO:0009312 | oligosaccharide biosynthetic process | 2/111 | 29/18888 | 0.012530015 | 0.07068354 | 0.052642512 | FBP1/B3GALT2 | 2 |
| BP | GO:0055074 | calcium ion homeostasis | 6/111 | 326/18888 | 0.012600183 | 0.070924174 | 0.052821728 | UMOD/CYP27B1/CALB1/PLCG2/CCL19/CCL21 | 6 |
| BP | GO:0007612 | learning | 4/111 | 153/18888 | 0.012699353 | 0.071326648 | 0.053121476 | FOS/PTGS2/PRKAR2B/PTN | 4 |
| BP | GO:0150076 | neuroinflammatory response | 3/111 | 83/18888 | 0.012913323 | 0.072370755 | 0.053899088 | PTGS2/IGF1/PLCG2 | 3 |
| BP | GO:0051251 | positive regulation of lymphocyte activation | 6/111 | 328/18888 | 0.012953432 | 0.0724314 | 0.053944254 | IGF1/PCK1/CR1/CCL2/CCL19/CCL21 | 6 |
| BP | GO:0071456 | cellular response to hypoxia | 4/111 | 154/18888 | 0.012980336 | 0.0724314 | 0.053944254 | FOS/PTGS2/PCK1/FABP1 | 4 |
| BP | GO:0006816 | calcium ion transport | 7/111 | 428/18888 | 0.013224892 | 0.073477956 | 0.054723691 | PTGS2/EGF/CYP27B1/PLCG2/CCL2/CCL19/CCL21 | 7 |
| BP | GO:0042692 | muscle cell differentiation | 7/111 | 428/18888 | 0.013224892 | 0.073477956 | 0.054723691 | LOX/NPHS1/RGS2/IGF1/TNNT2/FGF9/KRT19 | 7 |
| BP | GO:0072593 | reactive oxygen species metabolic process | 5/111 | 238/18888 | 0.013265248 | 0.073514156 | 0.054750651 | PDK4/HBB/DCXR/PLCG2/FBLN5 | 5 |
| BP | GO:0002437 | inflammatory response to antigenic stimulus | 3/111 | 84/18888 | 0.013335342 | 0.073514156 | 0.054750651 | CXCR2/FCGR3B/C3 | 3 |
| BP | GO:0065005 | protein-lipid complex assembly | 2/111 | 30/18888 | 0.013373987 | 0.073514156 | 0.054750651 | APOM/APOC1 | 2 |
| BP | GO:0071295 | cellular response to vitamin | 2/111 | 30/18888 | 0.013373987 | 0.073514156 | 0.054750651 | CYP27B1/TNC | 2 |
| BP | GO:0072012 | glomerulus vasculature development | 2/111 | 30/18888 | 0.013373987 | 0.073514156 | 0.054750651 | EGR1/TCF21 | 2 |
| BP | GO:0002429 | immune response-activating cell surface receptor signaling pathway | 6/111 | 332/18888 | 0.013680731 | 0.075040265 | 0.055887242 | CR1/FPR1/PLCG2/IGKC/FPR3/C3 | 6 |
| BP | GO:0002292 | T cell differentiation involved in immune response | 3/111 | 85/18888 | 0.013765171 | 0.075183499 | 0.055993917 | JUNB/PCK1/CCL19 | 3 |
| BP | GO:0007492 | endoderm development | 3/111 | 85/18888 | 0.013765171 | 0.075183499 | 0.055993917 | DUSP1/MMP2/FN1 | 3 |
| BP | GO:0071214 | cellular response to abiotic stimulus | 6/111 | 334/18888 | 0.014054911 | 0.076016392 | 0.056614225 | EGR1/PTGS2/FBP1/PCK1/MFAP4/MMP2 | 6 |
| BP | GO:0104004 | cellular response to environmental stimulus | 6/111 | 334/18888 | 0.014054911 | 0.076016392 | 0.056614225 | EGR1/PTGS2/FBP1/PCK1/MFAP4/MMP2 | 6 |
| BP | GO:0001516 | prostaglandin biosynthetic process | 2/111 | 31/18888 | 0.014242016 | 0.076016392 | 0.056614225 | PTGS2/PTGDS | 2 |
| BP | GO:0002313 | mature B cell differentiation involved in immune response | 2/111 | 31/18888 | 0.014242016 | 0.076016392 | 0.056614225 | CR1/PLCG2 | 2 |
| BP | GO:0003298 | physiological muscle hypertrophy | 2/111 | 31/18888 | 0.014242016 | 0.076016392 | 0.056614225 | RGS2/IGF1 | 2 |
| BP | GO:0003301 | physiological cardiac muscle hypertrophy | 2/111 | 31/18888 | 0.014242016 | 0.076016392 | 0.056614225 | RGS2/IGF1 | 2 |
| BP | GO:0010575 | positive regulation of vascular endothelial growth factor production | 2/111 | 31/18888 | 0.014242016 | 0.076016392 | 0.056614225 | PTGS2/C3 | 2 |
| BP | GO:0033238 | regulation of amine metabolic process | 2/111 | 31/18888 | 0.014242016 | 0.076016392 | 0.056614225 | NR4A2/BHMT | 2 |
| BP | GO:0046457 | prostanoid biosynthetic process | 2/111 | 31/18888 | 0.014242016 | 0.076016392 | 0.056614225 | PTGS2/PTGDS | 2 |
| BP | GO:0061049 | cell growth involved in cardiac muscle cell development | 2/111 | 31/18888 | 0.014242016 | 0.076016392 | 0.056614225 | RGS2/IGF1 | 2 |
| BP | GO:0072677 | eosinophil migration | 2/111 | 31/18888 | 0.014242016 | 0.076016392 | 0.056614225 | CCL2/CCL18 | 2 |
| BP | GO:0043434 | response to peptide hormone | 7/111 | 438/18888 | 0.014844198 | 0.079038984 | 0.058865341 | FOS/PDK4/EGR1/PTGS2/FBP1/PCK1/NR4A2 | 7 |
| BP | GO:0007179 | transforming growth factor beta receptor signaling pathway | 5/111 | 245/18888 | 0.01486963 | 0.079038984 | 0.058865341 | FOS/LOX/HPGD/ASPN/COL1A2 | 5 |
| BP | GO:0036294 | cellular response to decreased oxygen levels | 4/111 | 161/18888 | 0.015058704 | 0.079460126 | 0.059178992 | FOS/PTGS2/PCK1/FABP1 | 4 |
| BP | GO:0032372 | negative regulation of sterol transport | 2/111 | 32/18888 | 0.015133794 | 0.079460126 | 0.059178992 | EGF/APOC1 | 2 |
| BP | GO:0032375 | negative regulation of cholesterol transport | 2/111 | 32/18888 | 0.015133794 | 0.079460126 | 0.059178992 | EGF/APOC1 | 2 |
| BP | GO:0033032 | regulation of myeloid cell apoptotic process | 2/111 | 32/18888 | 0.015133794 | 0.079460126 | 0.059178992 | CXCR2/APOH | 2 |
| BP | GO:0061437 | renal system vasculature development | 2/111 | 32/18888 | 0.015133794 | 0.079460126 | 0.059178992 | EGR1/TCF21 | 2 |
| BP | GO:0061440 | kidney vasculature development | 2/111 | 32/18888 | 0.015133794 | 0.079460126 | 0.059178992 | EGR1/TCF21 | 2 |
| BP | GO:0021700 | developmental maturation | 6/111 | 341/18888 | 0.015420931 | 0.080803173 | 0.060179244 | IGF1/NR4A2/MMP2/CCL19/CCL21/C3 | 6 |
| BP | GO:2000106 | regulation of leukocyte apoptotic process | 3/111 | 89/18888 | 0.015562948 | 0.081053964 | 0.060366025 | CXCR2/CCL19/CCL21 | 3 |
| BP | GO:0034599 | cellular response to oxidative stress | 5/111 | 248/18888 | 0.015595413 | 0.081053964 | 0.060366025 | FOS/FABP1/NR4A2/MMP2/FBLN5 | 5 |
| BP | GO:0045444 | fat cell differentiation | 5/111 | 248/18888 | 0.015595413 | 0.081053964 | 0.060366025 | ZFP36/PTGS2/LPL/RGS2/NR4A2 | 5 |
| BP | GO:0048738 | cardiac muscle tissue development | 5/111 | 248/18888 | 0.015595413 | 0.081053964 | 0.060366025 | RGS2/TNNI1/IGF1/TNNT2/FGF9 | 5 |
| BP | GO:0007292 | female gamete generation | 4/111 | 163/18888 | 0.015688878 | 0.081053964 | 0.060366025 | IGF1/HPGD/MMP2/PTN | 4 |
| BP | GO:0030307 | positive regulation of cell growth | 4/111 | 163/18888 | 0.015688878 | 0.081053964 | 0.060366025 | S100A9/S100A8/IGF1/FN1 | 4 |
| BP | GO:0170033 | L-amino acid metabolic process | 4/111 | 163/18888 | 0.015688878 | 0.081053964 | 0.060366025 | HPD/GLYAT/BHMT/PRODH2 | 4 |
| BP | GO:1901617 | organic hydroxy compound biosynthetic process | 5/111 | 249/18888 | 0.015842516 | 0.081445609 | 0.060657707 | PCK1/NR4A2/CYP27B1/PLCG2/MOXD1 | 5 |
| BP | GO:0019362 | pyridine nucleotide metabolic process | 4/111 | 164/18888 | 0.016010108 | 0.081445609 | 0.060657707 | IGF1/ALDOB/FBP1/DCXR | 4 |
| BP | GO:0046496 | nicotinamide nucleotide metabolic process | 4/111 | 164/18888 | 0.016010108 | 0.081445609 | 0.060657707 | IGF1/ALDOB/FBP1/DCXR | 4 |
| BP | GO:0006096 | glycolytic process | 3/111 | 90/18888 | 0.016032087 | 0.081445609 | 0.060657707 | IGF1/ALDOB/FBP1 | 3 |
| BP | GO:0048145 | regulation of fibroblast proliferation | 3/111 | 90/18888 | 0.016032087 | 0.081445609 | 0.060657707 | IGF1/DACH1/FN1 | 3 |
| BP | GO:0001975 | response to amphetamine | 2/111 | 33/18888 | 0.016049018 | 0.081445609 | 0.060657707 | RGS2/NR4A2 | 2 |
| BP | GO:0034121 | regulation of toll-like receptor signaling pathway | 2/111 | 33/18888 | 0.016049018 | 0.081445609 | 0.060657707 | S100A9/S100A8 | 2 |
| BP | GO:0048566 | embryonic digestive tract development | 2/111 | 33/18888 | 0.016049018 | 0.081445609 | 0.060657707 | TCF21/FGF9 | 2 |
| BP | GO:0050891 | multicellular organismal-level water homeostasis | 2/111 | 33/18888 | 0.016049018 | 0.081445609 | 0.060657707 | UMOD/CYP4A11 | 2 |
| BP | GO:0010660 | regulation of muscle cell apoptotic process | 3/111 | 91/18888 | 0.016509132 | 0.08296402 | 0.061788564 | IGF1/CXCR2/APOH | 3 |
| BP | GO:0030901 | midbrain development | 3/111 | 91/18888 | 0.016509132 | 0.08296402 | 0.061788564 | CXCR2/NR4A2/FGF9 | 3 |
| BP | GO:0032273 | positive regulation of protein polymerization | 3/111 | 91/18888 | 0.016509132 | 0.08296402 | 0.061788564 | TPPP3/NPHS1/CCL21 | 3 |
| BP | GO:0055017 | cardiac muscle tissue growth | 3/111 | 91/18888 | 0.016509132 | 0.08296402 | 0.061788564 | RGS2/IGF1/FGF9 | 3 |
| BP | GO:0097006 | regulation of plasma lipoprotein particle levels | 3/111 | 91/18888 | 0.016509132 | 0.08296402 | 0.061788564 | LPL/APOM/APOC1 | 3 |
| BP | GO:0048732 | gland development | 7/111 | 449/18888 | 0.016784257 | 0.084182516 | 0.062696056 | EGF/PCK1/TCF21/MMP2/TNC/PTN/SERPINF1 | 7 |
| BP | GO:0034405 | response to fluid shear stress | 2/111 | 34/18888 | 0.016987387 | 0.084413824 | 0.062868325 | PTGS2/MMP2 | 2 |
| BP | GO:0046320 | regulation of fatty acid oxidation | 2/111 | 34/18888 | 0.016987387 | 0.084413824 | 0.062868325 | PDK4/FABP1 | 2 |
| BP | GO:0051450 | myoblast proliferation | 2/111 | 34/18888 | 0.016987387 | 0.084413824 | 0.062868325 | FOS/IGF1 | 2 |
| BP | GO:0055075 | potassium ion homeostasis | 2/111 | 34/18888 | 0.016987387 | 0.084413824 | 0.062868325 | UMOD/SLC12A3 | 2 |
| BP | GO:0046032 | ADP catabolic process | 3/111 | 92/18888 | 0.016994094 | 0.084413824 | 0.062868325 | IGF1/ALDOB/FBP1 | 3 |
| BP | GO:0002699 | positive regulation of immune effector process | 5/111 | 257/18888 | 0.017914065 | 0.08832873 | 0.065784004 | PCK1/CR1/PLCG2/CCL19/C3 | 5 |
| BP | GO:0045860 | positive regulation of protein kinase activity | 5/111 | 257/18888 | 0.017914065 | 0.08832873 | 0.065784004 | S100A12/IGF1/CHI3L1/EGF/CCL19 | 5 |
| BP | GO:0071398 | cellular response to fatty acid | 2/111 | 35/18888 | 0.017948601 | 0.08832873 | 0.065784004 | PDK4/LPL | 2 |
| BP | GO:0045765 | regulation of angiogenesis | 6/111 | 353/18888 | 0.017973015 | 0.08832873 | 0.065784004 | CHI3L1/CXCR2/APOH/THBS2/SERPINF1/C3 | 6 |
| BP | GO:0019364 | pyridine nucleotide catabolic process | 3/111 | 94/18888 | 0.017987814 | 0.08832873 | 0.065784004 | IGF1/ALDOB/FBP1 | 3 |
| BP | GO:0043502 | regulation of muscle adaptation | 3/111 | 94/18888 | 0.017987814 | 0.08832873 | 0.065784004 | RGS2/TNNI1/IGF1 | 3 |
| BP | GO:0072524 | pyridine-containing compound metabolic process | 4/111 | 170/18888 | 0.018024443 | 0.088340332 | 0.065792645 | IGF1/ALDOB/FBP1/DCXR | 4 |
| BP | GO:0045927 | positive regulation of growth | 5/111 | 258/18888 | 0.018185008 | 0.08895816 | 0.06625278 | S100A9/S100A8/IGF1/FGF9/FN1 | 5 |
| BP | GO:0009137 | purine nucleoside diphosphate catabolic process | 3/111 | 95/18888 | 0.018496589 | 0.089970203 | 0.067006513 | IGF1/ALDOB/FBP1 | 3 |
| BP | GO:0009181 | purine ribonucleoside diphosphate catabolic process | 3/111 | 95/18888 | 0.018496589 | 0.089970203 | 0.067006513 | IGF1/ALDOB/FBP1 | 3 |
| BP | GO:0071901 | negative regulation of protein serine/threonine kinase activity | 3/111 | 95/18888 | 0.018496589 | 0.089970203 | 0.067006513 | RGS2/DUSP1/PRKAR2B | 3 |
| BP | GO:0044272 | sulfur compound biosynthetic process | 4/111 | 172/18888 | 0.018729367 | 0.090930903 | 0.067722007 | PDK4/GSTA1/BHMT/FMO3 | 4 |
| BP | GO:0006882 | intracellular zinc ion homeostasis | 2/111 | 36/18888 | 0.018932366 | 0.091571558 | 0.068199143 | S100A9/S100A8 | 2 |
| BP | GO:0051968 | positive regulation of synaptic transmission, glutamatergic | 2/111 | 36/18888 | 0.018932366 | 0.091571558 | 0.068199143 | PTGS2/CCL2 | 2 |
| BP | GO:1903522 | regulation of blood circulation | 5/111 | 262/18888 | 0.01929589 | 0.093155063 | 0.06937848 | PTGS2/RGS2/EHD3/TNNT2/MMP2 | 5 |
| BP | GO:0072526 | pyridine-containing compound catabolic process | 3/111 | 97/18888 | 0.019538012 | 0.094147654 | 0.070117725 | IGF1/ALDOB/FBP1 | 3 |
| BP | GO:0002768 | immune response-regulating cell surface receptor signaling pathway | 6/111 | 360/18888 | 0.019588789 | 0.094216226 | 0.070168795 | CR1/FPR1/PLCG2/IGKC/FPR3/C3 | 6 |
| BP | GO:0002335 | mature B cell differentiation | 2/111 | 37/18888 | 0.019938388 | 0.094939798 | 0.070707685 | CR1/PLCG2 | 2 |
| BP | GO:0055023 | positive regulation of cardiac muscle tissue growth | 2/111 | 37/18888 | 0.019938388 | 0.094939798 | 0.070707685 | IGF1/FGF9 | 2 |
| BP | GO:0002696 | positive regulation of leukocyte activation | 6/111 | 362/18888 | 0.020068092 | 0.094939798 | 0.070707685 | IGF1/PCK1/CR1/CCL2/CCL19/CCL21 | 6 |
| BP | GO:1901342 | regulation of vasculature development | 6/111 | 362/18888 | 0.020068092 | 0.094939798 | 0.070707685 | CHI3L1/CXCR2/APOH/THBS2/SERPINF1/C3 | 6 |
| BP | GO:0006576 | biogenic amine metabolic process | 3/111 | 98/18888 | 0.020070671 | 0.094939798 | 0.070707685 | AGMAT/NR4A2/MOXD1 | 3 |
| BP | GO:0006942 | regulation of striated muscle contraction | 3/111 | 98/18888 | 0.020070671 | 0.094939798 | 0.070707685 | RGS2/EHD3/TNNI1 | 3 |
| BP | GO:0009191 | ribonucleoside diphosphate catabolic process | 3/111 | 98/18888 | 0.020070671 | 0.094939798 | 0.070707685 | IGF1/ALDOB/FBP1 | 3 |
| BP | GO:0010657 | muscle cell apoptotic process | 3/111 | 98/18888 | 0.020070671 | 0.094939798 | 0.070707685 | IGF1/CXCR2/APOH | 3 |
| BP | GO:0046031 | ADP metabolic process | 3/111 | 98/18888 | 0.020070671 | 0.094939798 | 0.070707685 | IGF1/ALDOB/FBP1 | 3 |
| BP | GO:0050806 | positive regulation of synaptic transmission | 4/111 | 177/18888 | 0.020565947 | 0.096926892 | 0.0721876 | PTGS2/CALB1/PTN/CCL2 | 4 |
| BP | GO:0071453 | cellular response to oxygen levels | 4/111 | 177/18888 | 0.020565947 | 0.096926892 | 0.0721876 | FOS/PTGS2/PCK1/FABP1 | 4 |
| BP | GO:0060419 | heart growth | 3/111 | 99/18888 | 0.020611302 | 0.096963386 | 0.072214779 | RGS2/IGF1/FGF9 | 3 |
| BP | GO:0002443 | leukocyte mediated immunity | 7/111 | 469/18888 | 0.02076178 | 0.097493387 | 0.072609505 | PTGDS/CR1/FCGR3B/PLCG2/IGKC/C7/C3 | 7 |
| BP | GO:0032885 | regulation of polysaccharide biosynthetic process | 2/111 | 38/18888 | 0.020966376 | 0.098096767 | 0.07305888 | IGF1/EGF | 2 |
| BP | GO:0034391 | regulation of smooth muscle cell apoptotic process | 2/111 | 38/18888 | 0.020966376 | 0.098096767 | 0.07305888 | IGF1/APOH | 2 |
| BP | GO:0050764 | regulation of phagocytosis | 3/111 | 100/18888 | 0.021159908 | 0.098644199 | 0.073466587 | PLCG2/CCL2/C3 | 3 |
| BP | GO:0072080 | nephron tubule development | 3/111 | 100/18888 | 0.021159908 | 0.098644199 | 0.073466587 | UMOD/TCF21/CALB1 | 3 |
| BP | GO:0032868 | response to insulin | 5/111 | 269/18888 | 0.021345802 | 0.099331187 | 0.073978231 | FOS/PDK4/EGR1/FBP1/PCK1 | 5 |
| BP | GO:0010906 | regulation of glucose metabolic process | 3/111 | 101/18888 | 0.021716492 | 0.100511878 | 0.074857566 | PDK4/IGF1/FBP1 | 3 |
| BP | GO:0042542 | response to hydrogen peroxide | 3/111 | 101/18888 | 0.021716492 | 0.100511878 | 0.074857566 | HBB/FABP1/MMP2 | 3 |
| BP | GO:0043255 | regulation of carbohydrate biosynthetic process | 3/111 | 101/18888 | 0.021716492 | 0.100511878 | 0.074857566 | IGF1/EGF/FBP1 | 3 |
| BP | GO:0032350 | regulation of hormone metabolic process | 2/111 | 39/18888 | 0.022016043 | 0.100991743 | 0.075214952 | EGR1/CYP27B1 | 2 |
| BP | GO:0033028 | myeloid cell apoptotic process | 2/111 | 39/18888 | 0.022016043 | 0.100991743 | 0.075214952 | CXCR2/APOH | 2 |
| BP | GO:0034390 | smooth muscle cell apoptotic process | 2/111 | 39/18888 | 0.022016043 | 0.100991743 | 0.075214952 | IGF1/APOH | 2 |
| BP | GO:0043388 | positive regulation of DNA binding | 2/111 | 39/18888 | 0.022016043 | 0.100991743 | 0.075214952 | IGF1/EGF | 2 |
| BP | GO:0045616 | regulation of keratinocyte differentiation | 2/111 | 39/18888 | 0.022016043 | 0.100991743 | 0.075214952 | ZFP36/CYP27B1 | 2 |
| BP | GO:0050878 | regulation of body fluid levels | 6/111 | 370/18888 | 0.022065347 | 0.101038124 | 0.075249495 | UMOD/HBB/KNG1/APOH/CYP4A11/PLCG2 | 6 |
| BP | GO:0042129 | regulation of T cell proliferation | 4/111 | 181/18888 | 0.022112419 | 0.101074144 | 0.075276322 | IGF1/CR1/VSIG4/CCL19 | 4 |
| BP | GO:0007200 | phospholipase C-activating G protein-coupled receptor signaling pathway | 3/111 | 102/18888 | 0.022281056 | 0.10112775 | 0.075316245 | CXCR2/FPR1/FPR3 | 3 |
| BP | GO:0009134 | nucleoside diphosphate catabolic process | 3/111 | 102/18888 | 0.022281056 | 0.10112775 | 0.075316245 | IGF1/ALDOB/FBP1 | 3 |
| BP | GO:0046620 | regulation of organ growth | 3/111 | 102/18888 | 0.022281056 | 0.10112775 | 0.075316245 | RGS2/IGF1/FGF9 | 3 |
| BP | GO:2000177 | regulation of neural precursor cell proliferation | 3/111 | 102/18888 | 0.022281056 | 0.10112775 | 0.075316245 | EGF/PTN/VCAN | 3 |
| BP | GO:0070167 | regulation of biomineral tissue development | 3/111 | 103/18888 | 0.022853601 | 0.103544081 | 0.07711584 | CYP27B1/PTN/ASPN | 3 |
| BP | GO:0045580 | regulation of T cell differentiation | 4/111 | 183/18888 | 0.022911645 | 0.103624949 | 0.077176068 | JUNB/PCK1/CR1/CCL19 | 4 |
| BP | GO:0045429 | positive regulation of nitric oxide biosynthetic process | 2/111 | 40/18888 | 0.023087103 | 0.103690857 | 0.077225153 | PTGS2/HBB | 2 |
| BP | GO:0045923 | positive regulation of fatty acid metabolic process | 2/111 | 40/18888 | 0.023087103 | 0.103690857 | 0.077225153 | PTGS2/FABP1 | 2 |
| BP | GO:0048246 | macrophage chemotaxis | 2/111 | 40/18888 | 0.023087103 | 0.103690857 | 0.077225153 | MMP2/CCL2 | 2 |
| BP | GO:0070884 | regulation of calcineurin-NFAT signaling cascade | 2/111 | 40/18888 | 0.023087103 | 0.103690857 | 0.077225153 | IGF1/PLCG2 | 2 |
| BP | GO:0032642 | regulation of chemokine production | 3/111 | 104/18888 | 0.023434126 | 0.104883987 | 0.078113753 | EGR1/LPL/APOD | 3 |
| BP | GO:0061326 | renal tubule development | 3/111 | 104/18888 | 0.023434126 | 0.104883987 | 0.078113753 | UMOD/TCF21/CALB1 | 3 |
| BP | GO:1901653 | cellular response to peptide | 6/111 | 376/18888 | 0.023648827 | 0.105661484 | 0.078692804 | FOS/PDK4/IGF1/FBP1/PCK1/NR4A2 | 6 |
| BP | GO:0032602 | chemokine production | 3/111 | 105/18888 | 0.024022631 | 0.106071648 | 0.078998279 | EGR1/LPL/APOD | 3 |
| BP | GO:1903131 | mononuclear cell differentiation | 7/111 | 484/18888 | 0.024147635 | 0.106071648 | 0.078998279 | FOS/EGR1/JUNB/PCK1/CR1/PLCG2/CCL19 | 7 |
| BP | GO:0009268 | response to pH | 2/111 | 41/18888 | 0.024179273 | 0.106071648 | 0.078998279 | SST/SERPINF1 | 2 |
| BP | GO:0032570 | response to progesterone | 2/111 | 41/18888 | 0.024179273 | 0.106071648 | 0.078998279 | FOS/FOSB | 2 |
| BP | GO:0032781 | positive regulation of ATP-dependent activity | 2/111 | 41/18888 | 0.024179273 | 0.106071648 | 0.078998279 | ALDOB/TNNT2 | 2 |
| BP | GO:0042417 | dopamine metabolic process | 2/111 | 41/18888 | 0.024179273 | 0.106071648 | 0.078998279 | NR4A2/MOXD1 | 2 |
| BP | GO:0060421 | positive regulation of heart growth | 2/111 | 41/18888 | 0.024179273 | 0.106071648 | 0.078998279 | IGF1/FGF9 | 2 |
| BP | GO:0072210 | metanephric nephron development | 2/111 | 41/18888 | 0.024179273 | 0.106071648 | 0.078998279 | EGR1/TCF21 | 2 |
| BP | GO:0106056 | regulation of calcineurin-mediated signaling | 2/111 | 41/18888 | 0.024179273 | 0.106071648 | 0.078998279 | IGF1/PLCG2 | 2 |
| BP | GO:1904407 | positive regulation of nitric oxide metabolic process | 2/111 | 41/18888 | 0.024179273 | 0.106071648 | 0.078998279 | PTGS2/HBB | 2 |
| BP | GO:0045936 | negative regulation of phosphate metabolic process | 6/111 | 378/18888 | 0.024193223 | 0.106071648 | 0.078998279 | RGS2/DUSP1/PRKAR2B/ALDOB/FBP1/APOC1 | 6 |
| BP | GO:0045785 | positive regulation of cell adhesion | 7/111 | 485/18888 | 0.02438612 | 0.106735854 | 0.079492956 | IGF1/PCK1/CR1/CCL2/CCL19/CCL21/FN1 | 7 |
| BP | GO:0010563 | negative regulation of phosphorus metabolic process | 6/111 | 379/18888 | 0.024468556 | 0.106915147 | 0.079626487 | RGS2/DUSP1/PRKAR2B/ALDOB/FBP1/APOC1 | 6 |
| BP | GO:0019395 | fatty acid oxidation | 3/111 | 106/18888 | 0.024619114 | 0.10739099 | 0.079980877 | PDK4/HAO2/FABP1 | 3 |
| BP | GO:0050867 | positive regulation of cell activation | 6/111 | 380/18888 | 0.024745987 | 0.107762084 | 0.080257254 | IGF1/PCK1/CR1/CCL2/CCL19/CCL21 | 6 |
| BP | GO:0048771 | tissue remodeling | 4/111 | 188/18888 | 0.024986175 | 0.10861245 | 0.080890576 | PDK4/UMOD/MMP2/PTN | 4 |
| BP | GO:0001818 | negative regulation of cytokine production | 6/111 | 381/18888 | 0.025025522 | 0.10861245 | 0.080890576 | ZFP36/IGF1/CR1/APOD/VSIG4/FN1 | 6 |
| BP | GO:0019751 | polyol metabolic process | 3/111 | 107/18888 | 0.025223571 | 0.10903592 | 0.08120596 | PCK1/CYP27B1/PLCG2 | 3 |
| BP | GO:0032735 | positive regulation of interleukin-12 production | 2/111 | 42/18888 | 0.025292273 | 0.10903592 | 0.08120596 | PLCG2/CCL19 | 2 |
| BP | GO:0048512 | circadian behavior | 2/111 | 42/18888 | 0.025292273 | 0.10903592 | 0.08120596 | EGR1/PTGDS | 2 |
| BP | GO:0071364 | cellular response to epidermal growth factor stimulus | 2/111 | 42/18888 | 0.025292273 | 0.10903592 | 0.08120596 | FOS/ZFP36 | 2 |
| BP | GO:1902107 | positive regulation of leukocyte differentiation | 4/111 | 190/18888 | 0.025846772 | 0.110870179 | 0.08257205 | FOS/PCK1/CR1/CCL19 | 4 |
| BP | GO:1903708 | positive regulation of hemopoiesis | 4/111 | 190/18888 | 0.025846772 | 0.110870179 | 0.08257205 | FOS/PCK1/CR1/CCL19 | 4 |
| BP | GO:2001242 | regulation of intrinsic apoptotic signaling pathway | 4/111 | 190/18888 | 0.025846772 | 0.110870179 | 0.08257205 | S100A9/PTGS2/S100A8/MMP2 | 4 |
| BP | GO:0006941 | striated muscle contraction | 4/111 | 191/18888 | 0.026283702 | 0.112557115 | 0.083828418 | RGS2/EHD3/TNNI1/TNNT2 | 4 |
| BP | GO:0045622 | regulation of T-helper cell differentiation | 2/111 | 43/18888 | 0.026425825 | 0.112921506 | 0.084099803 | JUNB/CCL19 | 2 |
| BP | GO:0032760 | positive regulation of tumor necrosis factor production | 3/111 | 109/18888 | 0.026456396 | 0.112921506 | 0.084099803 | LPL/PLCG2/CCL19 | 3 |
| BP | GO:0002822 | regulation of adaptive immune response based on somatic recombination of immune receptors built from immunoglobulin superfamily domains | 4/111 | 192/18888 | 0.026725064 | 0.113879693 | 0.084813425 | JUNB/CR1/CCL19/C3 | 4 |
| BP | GO:0009135 | purine nucleoside diphosphate metabolic process | 3/111 | 110/18888 | 0.027084754 | 0.114793296 | 0.085493844 | IGF1/ALDOB/FBP1 | 3 |
| BP | GO:0009179 | purine ribonucleoside diphosphate metabolic process | 3/111 | 110/18888 | 0.027084754 | 0.114793296 | 0.085493844 | IGF1/ALDOB/FBP1 | 3 |
| BP | GO:0048144 | fibroblast proliferation | 3/111 | 110/18888 | 0.027084754 | 0.114793296 | 0.085493844 | IGF1/DACH1/FN1 | 3 |
| BP | GO:0022407 | regulation of cell-cell adhesion | 7/111 | 496/18888 | 0.027117579 | 0.114793296 | 0.085493844 | IGF1/PCK1/CR1/VSIG4/CCL2/CCL19/CCL21 | 7 |
| BP | GO:0007622 | rhythmic behavior | 2/111 | 44/18888 | 0.027579654 | 0.115408803 | 0.08595225 | EGR1/PTGDS | 2 |
| BP | GO:0010799 | regulation of peptidyl-threonine phosphorylation | 2/111 | 44/18888 | 0.027579654 | 0.115408803 | 0.08595225 | CHI3L1/EGF | 2 |
| BP | GO:0010828 | positive regulation of glucose transmembrane transport | 2/111 | 44/18888 | 0.027579654 | 0.115408803 | 0.08595225 | IGF1/C3 | 2 |
| BP | GO:0014002 | astrocyte development | 2/111 | 44/18888 | 0.027579654 | 0.115408803 | 0.08595225 | S100A9/S100A8 | 2 |
| BP | GO:0045840 | positive regulation of mitotic nuclear division | 2/111 | 44/18888 | 0.027579654 | 0.115408803 | 0.08595225 | IGF1/EGF | 2 |
| BP | GO:0070741 | response to interleukin-6 | 2/111 | 44/18888 | 0.027579654 | 0.115408803 | 0.08595225 | CHI3L1/PCK1 | 2 |
| BP | GO:0071548 | response to dexamethasone | 2/111 | 44/18888 | 0.027579654 | 0.115408803 | 0.08595225 | PCK1/SERPINF1 | 2 |
| BP | GO:0030258 | lipid modification | 4/111 | 194/18888 | 0.027621114 | 0.115408803 | 0.08595225 | PDK4/HAO2/FABP1/APOD | 4 |
| BP | GO:0002444 | myeloid leukocyte mediated immunity | 3/111 | 112/18888 | 0.028365328 | 0.117944863 | 0.087841015 | PTGDS/FCGR3B/C3 | 3 |
| BP | GO:0045833 | negative regulation of lipid metabolic process | 3/111 | 112/18888 | 0.028365328 | 0.117944863 | 0.087841015 | CYP27B1/APOD/APOC1 | 3 |
| BP | GO:1905954 | positive regulation of lipid localization | 3/111 | 112/18888 | 0.028365328 | 0.117944863 | 0.087841015 | LPL/CYP4A11/C3 | 3 |
| BP | GO:0006739 | NADP metabolic process | 2/111 | 45/18888 | 0.028753486 | 0.118224064 | 0.088048954 | ALDOB/DCXR | 2 |
| BP | GO:0018198 | peptidyl-cysteine modification | 2/111 | 45/18888 | 0.028753486 | 0.118224064 | 0.088048954 | S100A9/S100A8 | 2 |
| BP | GO:0019432 | triglyceride biosynthetic process | 2/111 | 45/18888 | 0.028753486 | 0.118224064 | 0.088048954 | PCK1/C3 | 2 |
| BP | GO:0030574 | collagen catabolic process | 2/111 | 45/18888 | 0.028753486 | 0.118224064 | 0.088048954 | MMP2/MMP7 | 2 |
| BP | GO:0032309 | icosanoid secretion | 2/111 | 45/18888 | 0.028753486 | 0.118224064 | 0.088048954 | PTGS2/CYP4A11 | 2 |
| BP | GO:0032881 | regulation of polysaccharide metabolic process | 2/111 | 45/18888 | 0.028753486 | 0.118224064 | 0.088048954 | IGF1/EGF | 2 |
| BP | GO:0033173 | calcineurin-NFAT signaling cascade | 2/111 | 45/18888 | 0.028753486 | 0.118224064 | 0.088048954 | IGF1/PLCG2 | 2 |
| BP | GO:0031334 | positive regulation of protein-containing complex assembly | 4/111 | 197/18888 | 0.028998614 | 0.118930352 | 0.088574972 | TPPP3/NPHS1/PLCG2/CCL21 | 4 |
| BP | GO:1903557 | positive regulation of tumor necrosis factor superfamily cytokine production | 3/111 | 113/18888 | 0.02901753 | 0.118930352 | 0.088574972 | LPL/PLCG2/CCL19 | 3 |
| BP | GO:0051402 | neuron apoptotic process | 5/111 | 292/18888 | 0.029064343 | 0.118933138 | 0.088577046 | EGR1/NR4A2/MMP2/CCL2/COL6A3 | 5 |
| BP | GO:0051962 | positive regulation of nervous system development | 5/111 | 294/18888 | 0.029808848 | 0.121786387 | 0.090702042 | PTN/THBS2/SERPINF1/VCAN/FN1 | 5 |
| BP | GO:0070849 | response to epidermal growth factor | 2/111 | 46/18888 | 0.029947052 | 0.121964457 | 0.090834661 | FOS/ZFP36 | 2 |
| BP | GO:0140467 | integrated stress response signaling | 2/111 | 46/18888 | 0.029947052 | 0.121964457 | 0.090834661 | FOS/JUNB | 2 |
| BP | GO:0006520 | amino acid metabolic process | 5/111 | 295/18888 | 0.03018559 | 0.122742036 | 0.091413774 | HPD/AGMAT/GLYAT/BHMT/PRODH2 | 5 |
| BP | GO:0015908 | fatty acid transport | 3/111 | 115/18888 | 0.030345721 | 0.123005138 | 0.091609722 | PTGS2/FABP1/CYP4A11 | 3 |
| BP | GO:0032355 | response to estradiol | 3/111 | 115/18888 | 0.030345721 | 0.123005138 | 0.091609722 | PTGS2/HPGD/MMP2 | 3 |
| BP | GO:0046486 | glycerolipid metabolic process | 6/111 | 400/18888 | 0.030744166 | 0.124424586 | 0.092666875 | LPL/PCK1/APOH/PLCG2/APOC1/C3 | 6 |
| BP | GO:0048511 | rhythmic process | 5/111 | 297/18888 | 0.030948084 | 0.124543716 | 0.092755599 | EGR1/PTGDS/MMP2/PTN/SERPINF1 | 5 |
| BP | GO:0044344 | cellular response to fibroblast growth factor stimulus | 3/111 | 116/18888 | 0.03102169 | 0.124543716 | 0.092755599 | ZFP36/FGF9/CCL2 | 3 |
| BP | GO:0071887 | leukocyte apoptotic process | 3/111 | 116/18888 | 0.03102169 | 0.124543716 | 0.092755599 | CXCR2/CCL19/CCL21 | 3 |
| BP | GO:0006953 | acute-phase response | 2/111 | 47/18888 | 0.031160084 | 0.124543716 | 0.092755599 | PTGS2/FN1 | 2 |
| BP | GO:0016339 | calcium-dependent cell-cell adhesion via plasma membrane cell adhesion molecules | 2/111 | 47/18888 | 0.031160084 | 0.124543716 | 0.092755599 | CDH10/CDH6 | 2 |
| BP | GO:0045933 | positive regulation of muscle contraction | 2/111 | 47/18888 | 0.031160084 | 0.124543716 | 0.092755599 | PTGS2/RGS2 | 2 |
| BP | GO:0055010 | ventricular cardiac muscle tissue morphogenesis | 2/111 | 47/18888 | 0.031160084 | 0.124543716 | 0.092755599 | TNNI1/TNNT2 | 2 |
| BP | GO:1900271 | regulation of long-term synaptic potentiation | 2/111 | 47/18888 | 0.031160084 | 0.124543716 | 0.092755599 | CALB1/PTN | 2 |
| BP | GO:0002673 | regulation of acute inflammatory response | 2/111 | 48/18888 | 0.032392316 | 0.127123742 | 0.094677108 | PTGS2/C3 | 2 |
| BP | GO:0031295 | T cell costimulation | 2/111 | 48/18888 | 0.032392316 | 0.127123742 | 0.094677108 | CCL19/CCL21 | 2 |
| BP | GO:0035850 | epithelial cell differentiation involved in kidney development | 2/111 | 48/18888 | 0.032392316 | 0.127123742 | 0.094677108 | NPHS1/MAGI2 | 2 |
| BP | GO:0042311 | vasodilation | 2/111 | 48/18888 | 0.032392316 | 0.127123742 | 0.094677108 | RGS2/KNG1 | 2 |
| BP | GO:0043277 | apoptotic cell clearance | 2/111 | 48/18888 | 0.032392316 | 0.127123742 | 0.094677108 | CCL2/C3 | 2 |
| BP | GO:0043407 | negative regulation of MAP kinase activity | 2/111 | 48/18888 | 0.032392316 | 0.127123742 | 0.094677108 | RGS2/DUSP1 | 2 |
| BP | GO:0044331 | cell-cell adhesion mediated by cadherin | 2/111 | 48/18888 | 0.032392316 | 0.127123742 | 0.094677108 | CDH10/CDH6 | 2 |
| BP | GO:0046189 | phenol-containing compound biosynthetic process | 2/111 | 48/18888 | 0.032392316 | 0.127123742 | 0.094677108 | NR4A2/MOXD1 | 2 |
| BP | GO:0046578 | regulation of Ras protein signal transduction | 2/111 | 48/18888 | 0.032392316 | 0.127123742 | 0.094677108 | IGF1/FBP1 | 2 |
| BP | GO:0090279 | regulation of calcium ion import | 2/111 | 48/18888 | 0.032392316 | 0.127123742 | 0.094677108 | EGF/CCL2 | 2 |
| BP | GO:0097720 | calcineurin-mediated signaling | 2/111 | 48/18888 | 0.032392316 | 0.127123742 | 0.094677108 | IGF1/PLCG2 | 2 |
| BP | GO:0007088 | regulation of mitotic nuclear division | 3/111 | 118/18888 | 0.032397323 | 0.127123742 | 0.094677108 | DUSP1/IGF1/EGF | 3 |
| BP | GO:0048469 | cell maturation | 4/111 | 205/18888 | 0.032869231 | 0.12853731 | 0.095729882 | NR4A2/CCL19/CCL21/C3 | 4 |
| BP | GO:0006874 | intracellular calcium ion homeostasis | 5/111 | 302/18888 | 0.032907147 | 0.12853731 | 0.095729882 | UMOD/CALB1/PLCG2/CCL19/CCL21 | 5 |
| BP | GO:0071560 | cellular response to transforming growth factor beta stimulus | 5/111 | 302/18888 | 0.032907147 | 0.12853731 | 0.095729882 | FOS/LOX/HPGD/ASPN/COL1A2 | 5 |
| BP | GO:0043500 | muscle adaptation | 3/111 | 119/18888 | 0.033096963 | 0.128888172 | 0.095991191 | RGS2/TNNI1/IGF1 | 3 |
| BP | GO:0045582 | positive regulation of T cell differentiation | 3/111 | 119/18888 | 0.033096963 | 0.128888172 | 0.095991191 | PCK1/CR1/CCL19 | 3 |
| BP | GO:0033674 | positive regulation of kinase activity | 5/111 | 303/18888 | 0.033308061 | 0.129514601 | 0.096457733 | S100A12/IGF1/CHI3L1/EGF/CCL19 | 5 |
| BP | GO:0006911 | phagocytosis, engulfment | 2/111 | 49/18888 | 0.033643484 | 0.129645594 | 0.096555291 | PLCG2/C3 | 2 |
| BP | GO:0014075 | response to amine | 2/111 | 49/18888 | 0.033643484 | 0.129645594 | 0.096555291 | RGS2/NR4A2 | 2 |
| BP | GO:0030195 | negative regulation of blood coagulation | 2/111 | 49/18888 | 0.033643484 | 0.129645594 | 0.096555291 | KNG1/APOH | 2 |
| BP | GO:0031294 | lymphocyte costimulation | 2/111 | 49/18888 | 0.033643484 | 0.129645594 | 0.096555291 | CCL19/CCL21 | 2 |
| BP | GO:0032369 | negative regulation of lipid transport | 2/111 | 49/18888 | 0.033643484 | 0.129645594 | 0.096555291 | EGF/APOC1 | 2 |
| BP | GO:0042551 | neuron maturation | 2/111 | 49/18888 | 0.033643484 | 0.129645594 | 0.096555291 | NR4A2/C3 | 2 |
| BP | GO:0009185 | ribonucleoside diphosphate metabolic process | 3/111 | 120/18888 | 0.033804467 | 0.130071517 | 0.096872503 | IGF1/ALDOB/FBP1 | 3 |
| BP | GO:0002819 | regulation of adaptive immune response | 4/111 | 207/18888 | 0.03388195 | 0.130175361 | 0.096949842 | JUNB/CR1/CCL19/C3 | 4 |
| BP | GO:0014911 | positive regulation of smooth muscle cell migration | 2/111 | 50/18888 | 0.034913329 | 0.133145801 | 0.099162117 | IGF1/FGF9 | 2 |
| BP | GO:0032924 | activin receptor signaling pathway | 2/111 | 50/18888 | 0.034913329 | 0.133145801 | 0.099162117 | MAGI2/FGF9 | 2 |
| BP | GO:0071320 | cellular response to cAMP | 2/111 | 50/18888 | 0.034913329 | 0.133145801 | 0.099162117 | FBP1/PCK1 | 2 |
| BP | GO:1900047 | negative regulation of hemostasis | 2/111 | 50/18888 | 0.034913329 | 0.133145801 | 0.099162117 | KNG1/APOH | 2 |
| BP | GO:2000404 | regulation of T cell migration | 2/111 | 50/18888 | 0.034913329 | 0.133145801 | 0.099162117 | APOD/CCL21 | 2 |
| BP | GO:0071559 | response to transforming growth factor beta | 5/111 | 309/18888 | 0.035777689 | 0.136046212 | 0.101322237 | FOS/LOX/HPGD/ASPN/COL1A2 | 5 |
| BP | GO:0045834 | positive regulation of lipid metabolic process | 3/111 | 123/18888 | 0.035974031 | 0.136046212 | 0.101322237 | PTGS2/PCK1/FABP1 | 3 |
| BP | GO:0030217 | T cell differentiation | 5/111 | 310/18888 | 0.036200029 | 0.136046212 | 0.101322237 | EGR1/JUNB/PCK1/CR1/CCL19 | 5 |
| BP | GO:0006110 | regulation of glycolytic process | 2/111 | 51/18888 | 0.036201591 | 0.136046212 | 0.101322237 | IGF1/FBP1 | 2 |
| BP | GO:0006636 | unsaturated fatty acid biosynthetic process | 2/111 | 51/18888 | 0.036201591 | 0.136046212 | 0.101322237 | PTGS2/PTGDS | 2 |
| BP | GO:0030811 | regulation of nucleotide catabolic process | 2/111 | 51/18888 | 0.036201591 | 0.136046212 | 0.101322237 | IGF1/FBP1 | 2 |
| BP | GO:0033121 | regulation of purine nucleotide catabolic process | 2/111 | 51/18888 | 0.036201591 | 0.136046212 | 0.101322237 | IGF1/FBP1 | 2 |
| BP | GO:0035987 | endodermal cell differentiation | 2/111 | 51/18888 | 0.036201591 | 0.136046212 | 0.101322237 | MMP2/FN1 | 2 |
| BP | GO:0045214 | sarcomere organization | 2/111 | 51/18888 | 0.036201591 | 0.136046212 | 0.101322237 | TNNT2/KRT19 | 2 |
| BP | GO:2001238 | positive regulation of extrinsic apoptotic signaling pathway | 2/111 | 51/18888 | 0.036201591 | 0.136046212 | 0.101322237 | G0S2/ATF3 | 2 |
| BP | GO:0071375 | cellular response to peptide hormone stimulus | 5/111 | 311/18888 | 0.036625449 | 0.137438731 | 0.102359334 | FOS/PDK4/FBP1/PCK1/NR4A2 | 5 |
| BP | GO:0002286 | T cell activation involved in immune response | 3/111 | 124/18888 | 0.036712851 | 0.137566469 | 0.102454469 | JUNB/PCK1/CCL19 | 3 |
| BP | GO:0042098 | T cell proliferation | 4/111 | 213/18888 | 0.037028776 | 0.138147881 | 0.102887483 | IGF1/CR1/VSIG4/CCL19 | 4 |
| BP | GO:0051348 | negative regulation of transferase activity | 4/111 | 213/18888 | 0.037028776 | 0.138147881 | 0.102887483 | ZFP36/RGS2/DUSP1/PRKAR2B | 4 |
| BP | GO:0071383 | cellular response to steroid hormone stimulus | 4/111 | 213/18888 | 0.037028776 | 0.138147881 | 0.102887483 | ZFP36/PCK1/TCF21/SERPINF1 | 4 |
| BP | GO:0022612 | gland morphogenesis | 3/111 | 125/18888 | 0.037459462 | 0.13913045 | 0.103619264 | MMP2/TNC/PTN | 3 |
| BP | GO:0051146 | striated muscle cell differentiation | 5/111 | 313/18888 | 0.037485545 | 0.13913045 | 0.103619264 | NPHS1/RGS2/IGF1/TNNT2/KRT19 | 5 |
| BP | GO:0006968 | cellular defense response | 2/111 | 52/18888 | 0.037508015 | 0.13913045 | 0.103619264 | UMOD/CXCR2 | 2 |
| BP | GO:0030838 | positive regulation of actin filament polymerization | 2/111 | 52/18888 | 0.037508015 | 0.13913045 | 0.103619264 | NPHS1/CCL21 | 2 |
| BP | GO:0045619 | regulation of lymphocyte differentiation | 4/111 | 215/18888 | 0.038114017 | 0.141175194 | 0.105142115 | JUNB/PCK1/CR1/CCL19 | 4 |
| BP | GO:0046622 | positive regulation of organ growth | 2/111 | 53/18888 | 0.038832347 | 0.143013986 | 0.10651158 | IGF1/FGF9 | 2 |
| BP | GO:0050819 | negative regulation of coagulation | 2/111 | 53/18888 | 0.038832347 | 0.143013986 | 0.10651158 | KNG1/APOH | 2 |
| BP | GO:0050850 | positive regulation of calcium-mediated signaling | 2/111 | 53/18888 | 0.038832347 | 0.143013986 | 0.10651158 | IGF1/PLCG2 | 2 |
| BP | GO:1902692 | regulation of neuroblast proliferation | 2/111 | 53/18888 | 0.038832347 | 0.143013986 | 0.10651158 | PTN/VCAN | 2 |
| BP | GO:0009132 | nucleoside diphosphate metabolic process | 3/111 | 127/18888 | 0.038975982 | 0.143134021 | 0.106600978 | IGF1/ALDOB/FBP1 | 3 |
| BP | GO:0071902 | positive regulation of protein serine/threonine kinase activity | 3/111 | 127/18888 | 0.038975982 | 0.143134021 | 0.106600978 | S100A12/EGF/CCL19 | 3 |
| BP | GO:0009395 | phospholipid catabolic process | 2/111 | 54/18888 | 0.040174335 | 0.146284514 | 0.108947349 | PLCG2/APOC1 | 2 |
| BP | GO:0010656 | negative regulation of muscle cell apoptotic process | 2/111 | 54/18888 | 0.040174335 | 0.146284514 | 0.108947349 | IGF1/APOH | 2 |
| BP | GO:0010883 | regulation of lipid storage | 2/111 | 54/18888 | 0.040174335 | 0.146284514 | 0.108947349 | LPL/C3 | 2 |
| BP | GO:0048146 | positive regulation of fibroblast proliferation | 2/111 | 54/18888 | 0.040174335 | 0.146284514 | 0.108947349 | IGF1/FN1 | 2 |
| BP | GO:0050873 | brown fat cell differentiation | 2/111 | 54/18888 | 0.040174335 | 0.146284514 | 0.108947349 | PTGS2/RGS2 | 2 |
| BP | GO:1904645 | response to amyloid-beta | 2/111 | 54/18888 | 0.040174335 | 0.146284514 | 0.108947349 | IGF1/MMP2 | 2 |
| BP | GO:0050679 | positive regulation of epithelial cell proliferation | 4/111 | 219/18888 | 0.040339037 | 0.146677063 | 0.109239706 | IGF1/EGF/FGF9/PTN | 4 |
| BP | GO:0051209 | release of sequestered calcium ion into cytosol | 3/111 | 129/18888 | 0.040523449 | 0.147140074 | 0.109584539 | PLCG2/CCL19/CCL21 | 3 |
| BP | GO:0051283 | negative regulation of sequestering of calcium ion | 3/111 | 130/18888 | 0.04130874 | 0.149544634 | 0.111375367 | PLCG2/CCL19/CCL21 | 3 |
| BP | GO:0042060 | wound healing | 6/111 | 430/18888 | 0.041412129 | 0.149544634 | 0.111375367 | HBB/IGF1/KNG1/APOH/PLCG2/FN1 | 6 |
| BP | GO:0034381 | plasma lipoprotein particle clearance | 2/111 | 55/18888 | 0.041533731 | 0.149544634 | 0.111375367 | APOM/APOC1 | 2 |
| BP | GO:0046460 | neutral lipid biosynthetic process | 2/111 | 55/18888 | 0.041533731 | 0.149544634 | 0.111375367 | PCK1/C3 | 2 |
| BP | GO:0046463 | acylglycerol biosynthetic process | 2/111 | 55/18888 | 0.041533731 | 0.149544634 | 0.111375367 | PCK1/C3 | 2 |
| BP | GO:0060425 | lung morphogenesis | 2/111 | 55/18888 | 0.041533731 | 0.149544634 | 0.111375367 | TCF21/TNC | 2 |
| BP | GO:0001704 | formation of primary germ layer | 3/111 | 131/18888 | 0.042101709 | 0.15137825 | 0.112740978 | DUSP1/MMP2/FN1 | 3 |
| BP | GO:0030098 | lymphocyte differentiation | 6/111 | 432/18888 | 0.042197025 | 0.151509652 | 0.112838841 | EGR1/JUNB/PCK1/CR1/PLCG2/CCL19 | 6 |
| BP | GO:0019730 | antimicrobial humoral response | 3/111 | 132/18888 | 0.042902335 | 0.152583062 | 0.113638277 | S100A12/S100A9/RNASE6 | 3 |
| BP | GO:0030301 | cholesterol transport | 3/111 | 132/18888 | 0.042902335 | 0.152583062 | 0.113638277 | EGF/APOM/APOC1 | 3 |
| BP | GO:0051282 | regulation of sequestering of calcium ion | 3/111 | 132/18888 | 0.042902335 | 0.152583062 | 0.113638277 | PLCG2/CCL19/CCL21 | 3 |
| BP | GO:0006584 | catecholamine metabolic process | 2/111 | 56/18888 | 0.042910287 | 0.152583062 | 0.113638277 | NR4A2/MOXD1 | 2 |
| BP | GO:0009712 | catechol-containing compound metabolic process | 2/111 | 56/18888 | 0.042910287 | 0.152583062 | 0.113638277 | NR4A2/MOXD1 | 2 |
| BP | GO:0043370 | regulation of CD4-positive, alpha-beta T cell differentiation | 2/111 | 56/18888 | 0.042910287 | 0.152583062 | 0.113638277 | JUNB/CCL19 | 2 |
| BP | GO:1902895 | positive regulation of miRNA transcription | 2/111 | 56/18888 | 0.042910287 | 0.152583062 | 0.113638277 | FOS/EGR1 | 2 |
| BP | GO:0006790 | sulfur compound metabolic process | 5/111 | 326/18888 | 0.043378957 | 0.154037123 | 0.114721209 | PDK4/GSTA1/GLYAT/BHMT/FMO3 | 5 |
| BP | GO:0042476 | odontogenesis | 3/111 | 133/18888 | 0.043710598 | 0.154788353 | 0.115280697 | TNC/ASPN/COL1A2 | 3 |
| BP | GO:0045621 | positive regulation of lymphocyte differentiation | 3/111 | 133/18888 | 0.043710598 | 0.154788353 | 0.115280697 | PCK1/CR1/CCL19 | 3 |
| BP | GO:0071222 | cellular response to lipopolysaccharide | 4/111 | 225/18888 | 0.043813016 | 0.154938212 | 0.115392307 | ZFP36/PLCG2/MRC1/CCL2 | 4 |
| BP | GO:0034332 | adherens junction organization | 2/111 | 57/18888 | 0.044303759 | 0.156132761 | 0.116281963 | CDH10/CDH6 | 2 |
| BP | GO:0170040 | proteinogenic amino acid catabolic process | 2/111 | 57/18888 | 0.044303759 | 0.156132761 | 0.116281963 | HPD/PRODH2 | 2 |
| BP | GO:1902105 | regulation of leukocyte differentiation | 5/111 | 328/18888 | 0.044332499 | 0.156132761 | 0.116281963 | FOS/JUNB/PCK1/CR1/CCL19 | 5 |
| BP | GO:0007596 | blood coagulation | 4/111 | 226/18888 | 0.044407932 | 0.15618506 | 0.116320914 | HBB/KNG1/APOH/PLCG2 | 4 |
| BP | GO:0008202 | steroid metabolic process | 5/111 | 329/18888 | 0.044813978 | 0.157398412 | 0.117224574 | EGR1/HSD17B14/CYP27B1/CYP2B6/APOC1 | 5 |
| BP | GO:0042742 | defense response to bacterium | 5/111 | 330/18888 | 0.045298599 | 0.158848038 | 0.118304202 | S100A12/S100A9/UMOD/S100A8/RNASE6 | 5 |
| BP | GO:0009154 | purine ribonucleotide catabolic process | 3/111 | 135/18888 | 0.045349944 | 0.158848038 | 0.118304202 | IGF1/ALDOB/FBP1 | 3 |
| BP | GO:0042306 | regulation of protein import into nucleus | 2/111 | 58/18888 | 0.045713905 | 0.159472864 | 0.118769549 | PTGS2/APOD | 2 |
| BP | GO:0048008 | platelet-derived growth factor receptor signaling pathway | 2/111 | 58/18888 | 0.045713905 | 0.159472864 | 0.118769549 | LOX/APOD | 2 |
| BP | GO:0099024 | plasma membrane invagination | 2/111 | 58/18888 | 0.045713905 | 0.159472864 | 0.118769549 | PLCG2/C3 | 2 |
| BP | GO:0051208 | sequestering of calcium ion | 3/111 | 136/18888 | 0.046180982 | 0.160884558 | 0.119820927 | PLCG2/CCL19/CCL21 | 3 |
| BP | GO:0043547 | positive regulation of GTPase activity | 4/111 | 230/18888 | 0.046833046 | 0.162716433 | 0.121185241 | CCL2/CCL18/CCL19/CCL21 | 4 |
| BP | GO:0051091 | positive regulation of DNA-binding transcription factor activity | 4/111 | 230/18888 | 0.046833046 | 0.162716433 | 0.121185241 | S100A12/S100A9/S100A8/PLCG2 | 4 |
| BP | GO:0003229 | ventricular cardiac muscle tissue development | 2/111 | 59/18888 | 0.047140484 | 0.163125056 | 0.121489569 | TNNI1/TNNT2 | 2 |
| BP | GO:0038066 | p38MAPK cascade | 2/111 | 59/18888 | 0.047140484 | 0.163125056 | 0.121489569 | ZFP36/DUSP1 | 2 |
| BP | GO:0045604 | regulation of epidermal cell differentiation | 2/111 | 59/18888 | 0.047140484 | 0.163125056 | 0.121489569 | ZFP36/CYP27B1 | 2 |
| BP | GO:0045861 | negative regulation of proteolysis | 4/111 | 231/18888 | 0.04745068 | 0.163758838 | 0.121961586 | KNG1/FABP1/CR1/SERPINF1 | 4 |
| BP | GO:0050817 | coagulation | 4/111 | 231/18888 | 0.04745068 | 0.163758838 | 0.121961586 | HBB/KNG1/APOH/PLCG2 | 4 |
| BP | GO:0019722 | calcium-mediated signaling | 4/111 | 232/18888 | 0.048072851 | 0.165684237 | 0.123395553 | IGF1/CXCR2/CXCR1/PLCG2 | 4 |
| BP | GO:0006940 | regulation of smooth muscle contraction | 2/111 | 60/18888 | 0.048583258 | 0.166774486 | 0.12420753 | PTGS2/RGS2 | 2 |
| BP | GO:0043113 | receptor clustering | 2/111 | 60/18888 | 0.048583258 | 0.166774486 | 0.12420753 | MAGI2/PTN | 2 |
| BP | GO:1905517 | macrophage migration | 2/111 | 60/18888 | 0.048583258 | 0.166774486 | 0.12420753 | MMP2/CCL2 | 2 |
| BP | GO:0007599 | hemostasis | 4/111 | 233/18888 | 0.048699558 | 0.166797205 | 0.124224451 | HBB/KNG1/APOH/PLCG2 | 4 |
| BP | GO:0042157 | lipoprotein metabolic process | 3/111 | 139/18888 | 0.048719277 | 0.166797205 | 0.124224451 | APOD/APOM/APOC1 | 3 |
| BP | GO:0043467 | regulation of generation of precursor metabolites and energy | 3/111 | 140/18888 | 0.049580353 | 0.169520093 | 0.126252358 | IGF1/ALDOB/FBP1 | 3 |
| CC | GO:0062023 | collagen-containing extracellular matrix | 20/110 | 428/19894 | 2.16E-13 | 4.44E-11 | 3.91E-11 | S100A9/LOX/S100A8/KNG1/APOH/AEBP1/MFAP4/MMP2/TNC/TGFBI/THBS2/SERPINF1/ASPN/CPA3/FBLN5/COL6A3/COL1A2/VCAN/LUM/FN1 | 20 |
| CC | GO:0034774 | secretory granule lumen | 11/110 | 322/19894 | 1.73E-06 | 0.000100269 | 8.81E-05 | ALB/S100A12/S100A9/S100A8/IGF1/CHI3L1/EGF/KNG1/APOH/C3/FN1 | 11 |
| CC | GO:0060205 | cytoplasmic vesicle lumen | 11/110 | 325/19894 | 1.89E-06 | 0.000100269 | 8.81E-05 | ALB/S100A12/S100A9/S100A8/IGF1/CHI3L1/EGF/KNG1/APOH/C3/FN1 | 11 |
| CC | GO:0031983 | vesicle lumen | 11/110 | 326/19894 | 1.95E-06 | 0.000100269 | 8.81E-05 | ALB/S100A12/S100A9/S100A8/IGF1/CHI3L1/EGF/KNG1/APOH/C3/FN1 | 11 |
| CC | GO:0034361 | very-low-density lipoprotein particle | 4/110 | 20/19894 | 4.00E-06 | 0.000137474 | 0.000120825 | LPL/APOH/APOM/APOC1 | 4 |
| CC | GO:0034385 | triglyceride-rich plasma lipoprotein particle | 4/110 | 20/19894 | 4.00E-06 | 0.000137474 | 0.000120825 | LPL/APOH/APOM/APOC1 | 4 |
| CC | GO:0031091 | platelet alpha granule | 6/110 | 91/19894 | 1.13E-05 | 0.000333626 | 0.000293223 | ALB/IGF1/EGF/KNG1/THBS2/FN1 | 6 |
| CC | GO:0072562 | blood microparticle | 7/110 | 144/19894 | 1.54E-05 | 0.000395777 | 0.000347847 | ALB/AFM/HBB/KNG1/IGKC/C3/FN1 | 7 |
| CC | GO:0031093 | platelet alpha granule lumen | 5/110 | 67/19894 | 3.47E-05 | 0.000780652 | 0.000686112 | ALB/IGF1/EGF/KNG1/FN1 | 5 |
| CC | GO:0042627 | chylomicron | 3/110 | 13/19894 | 4.52E-05 | 0.000780652 | 0.000686112 | LPL/APOH/APOC1 | 3 |
| CC | GO:0034358 | plasma lipoprotein particle | 4/110 | 36/19894 | 4.55E-05 | 0.000780652 | 0.000686112 | LPL/APOH/APOM/APOC1 | 4 |
| CC | GO:1990777 | lipoprotein particle | 4/110 | 36/19894 | 4.55E-05 | 0.000780652 | 0.000686112 | LPL/APOH/APOM/APOC1 | 4 |
| CC | GO:0005788 | endoplasmic reticulum lumen | 9/110 | 313/19894 | 6.08E-05 | 0.000922484 | 0.000810768 | ALB/PTGS2/KNG1/TNC/COL6A3/COL1A2/VCAN/C3/FN1 | 9 |
| CC | GO:0032994 | protein-lipid complex | 4/110 | 39/19894 | 6.27E-05 | 0.000922484 | 0.000810768 | LPL/APOH/APOM/APOC1 | 4 |
| CC | GO:0005604 | basement membrane | 5/110 | 92/19894 | 0.000158247 | 0.002173264 | 0.001910073 | TNC/TGFBI/THBS2/SERPINF1/FN1 | 5 |
| CC | GO:0034364 | high-density lipoprotein particle | 3/110 | 26/19894 | 0.000389796 | 0.005018626 | 0.004410852 | APOH/APOM/APOC1 | 3 |
| CC | GO:0005581 | collagen trimer | 4/110 | 86/19894 | 0.001326337 | 0.016072089 | 0.014125699 | LOX/COL6A3/COL1A2/LUM | 4 |
| CC | GO:0098966 | perisynaptic extracellular matrix | 2/110 | 11/19894 | 0.001612909 | 0.018458852 | 0.016223416 | TNC/VCAN | 2 |
| CC | GO:0005583 | fibrillar collagen trimer | 2/110 | 12/19894 | 0.001928512 | 0.018917785 | 0.016626771 | COL1A2/LUM | 2 |
| CC | GO:0098643 | banded collagen fibril | 2/110 | 12/19894 | 0.001928512 | 0.018917785 | 0.016626771 | COL1A2/LUM | 2 |
| CC | GO:0099535 | synapse-associated extracellular matrix | 2/110 | 12/19894 | 0.001928512 | 0.018917785 | 0.016626771 | TNC/VCAN | 2 |
| CC | GO:0098644 | complex of collagen trimers | 2/110 | 21/19894 | 0.005940293 | 0.055622741 | 0.048886619 | COL1A2/LUM | 2 |
| CC | GO:0005865 | striated muscle thin filament | 2/110 | 22/19894 | 0.006510859 | 0.058314654 | 0.051252532 | TNNI1/TNNT2 | 2 |
| CC | GO:0030667 | secretory granule membrane | 6/110 | 317/19894 | 0.008383433 | 0.071957797 | 0.063243439 | CXCR2/CXCR1/CR1/FPR1/FCGR3B/MOXD1 | 6 |
| CC | GO:0036379 | myofilament | 2/110 | 26/19894 | 0.00902958 | 0.074403742 | 0.065393171 | TNNI1/TNNT2 | 2 |
| CC | GO:0098982 | GABA-ergic synapse | 3/110 | 79/19894 | 0.009593105 | 0.07600691 | 0.066802189 | CDH10/SST/CALB1 | 3 |
| CC | GO:0016342 | catenin complex | 2/110 | 31/19894 | 0.012689649 | 0.096817322 | 0.085092383 | CDH10/CDH6 | 2 |
| CC | GO:0005796 | Golgi lumen | 3/110 | 106/19894 | 0.02103288 | 0.154741901 | 0.136002079 | UMOD/VCAN/LUM | 3 |
| CC | GO:0097546 | ciliary base | 2/110 | 47/19894 | 0.027862234 | 0.197917935 | 0.173949335 | PRKAR2B/MAGI2 | 2 |
| CC | GO:0005782 | peroxisomal matrix | 2/110 | 51/19894 | 0.032398589 | 0.21513637 | 0.189082553 | HAO2/FABP1 | 2 |
| CC | GO:0031907 | microbody lumen | 2/110 | 51/19894 | 0.032398589 | 0.21513637 | 0.189082553 | HAO2/FABP1 | 2 |
| CC | GO:0030017 | sarcomere | 4/110 | 219/19894 | 0.033419242 | 0.21513637 | 0.189082553 | TNNI1/TNNT2/KRT19/MMP2 | 4 |
| CC | GO:0030016 | myofibril | 4/110 | 240/19894 | 0.044315558 | 0.271936623 | 0.239004084 | TNNI1/TNNT2/KRT19/MMP2 | 4 |
| CC | GO:0101003 | ficolin-1-rich granule membrane | 2/110 | 61/19894 | 0.044882744 | 0.271936623 | 0.239004084 | CR1/FPR1 | 2 |
| MF | GO:0005201 | extracellular matrix structural constituent | 13/109 | 166/18522 | 1.66E-11 | 5.28E-09 | 4.33E-09 | CHI3L1/AEBP1/MFAP4/TNC/TGFBI/THBS2/ASPN/FBLN5/COL6A3/COL1A2/VCAN/LUM/FN1 | 13 |
| MF | GO:0005504 | fatty acid binding | 7/109 | 49/18522 | 1.41E-08 | 2.23E-06 | 1.83E-06 | ALB/S100A9/GSTA1/S100A8/PTGDS/FABP1/APOC1 | 7 |
| MF | GO:0050786 | RAGE receptor binding | 4/109 | 10/18522 | 2.32E-07 | 2.45E-05 | 2.01E-05 | S100A12/S100A9/S100A8/FPR1 | 4 |
| MF | GO:0033293 | monocarboxylic acid binding | 7/109 | 81/18522 | 4.89E-07 | 3.88E-05 | 3.18E-05 | ALB/S100A9/GSTA1/S100A8/PTGDS/FABP1/APOC1 | 7 |
| MF | GO:0016209 | antioxidant activity | 7/109 | 85/18522 | 6.81E-07 | 4.32E-05 | 3.54E-05 | ALB/S100A9/PTGS2/GSTA1/HBB/FABP1/APOM | 7 |
| MF | GO:0005518 | collagen binding | 6/109 | 68/18522 | 2.94E-06 | 0.00015547 | 0.000127515 | LOX/PCOLCE2/AEBP1/TGFBI/LUM/FN1 | 6 |
| MF | GO:0043177 | organic acid binding | 9/109 | 208/18522 | 3.86E-06 | 0.000174863 | 0.000143421 | ALB/S100A9/GSTA1/S100A8/HBB/PTGDS/PCK1/FABP1/APOC1 | 9 |
| MF | GO:0008201 | heparin binding | 8/109 | 174/18522 | 8.76E-06 | 0.000346973 | 0.000284584 | LPL/KNG1/PCOLCE2/APOH/FGF9/PTN/THBS2/FN1 | 8 |
| MF | GO:0005539 | glycosaminoglycan binding | 9/109 | 239/18522 | 1.19E-05 | 0.000417864 | 0.000342728 | LPL/KNG1/PCOLCE2/APOH/FGF9/PTN/THBS2/VCAN/FN1 | 9 |
| MF | GO:0031406 | carboxylic acid binding | 8/109 | 196/18522 | 2.08E-05 | 0.000658881 | 0.000540407 | ALB/S100A9/GSTA1/S100A8/PTGDS/PCK1/FABP1/APOC1 | 8 |
| MF | GO:0005178 | integrin binding | 7/109 | 157/18522 | 3.97E-05 | 0.001108517 | 0.000909194 | ESM1/IGF1/TNC/TGFBI/PTN/FBLN5/FN1 | 7 |
| MF | GO:0004875 | complement receptor activity | 3/109 | 12/18522 | 4.20E-05 | 0.001108517 | 0.000909194 | CR1/FPR1/FPR3 | 3 |
| MF | GO:0036041 | long-chain fatty acid binding | 3/109 | 17/18522 | 0.00012695 | 0.003095636 | 0.002539008 | S100A9/S100A8/FABP1 | 3 |
| MF | GO:1901681 | sulfur compound binding | 8/109 | 267/18522 | 0.00018173 | 0.004114887 | 0.003374986 | LPL/KNG1/PCOLCE2/APOH/FGF9/PTN/THBS2/FN1 | 8 |
| MF | GO:0008009 | chemokine activity | 4/109 | 49/18522 | 0.000196087 | 0.004143972 | 0.003398841 | CCL2/CCL18/CCL19/CCL21 | 4 |
| MF | GO:0048020 | CCR chemokine receptor binding | 4/109 | 50/18522 | 0.000212178 | 0.004203771 | 0.003447888 | CCL2/CCL18/CCL19/CCL21 | 4 |
| MF | GO:0140375 | immune receptor activity | 6/109 | 151/18522 | 0.000269851 | 0.004955958 | 0.004064824 | CXCR2/CXCR1/CR1/FPR1/FCGR3B/FPR3 | 6 |
| MF | GO:0030021 | extracellular matrix structural constituent conferring compression resistance | 3/109 | 22/18522 | 0.000281411 | 0.004955958 | 0.004064824 | ASPN/VCAN/LUM | 3 |
| MF | GO:0019957 | C-C chemokine binding | 3/109 | 24/18522 | 0.000366701 | 0.006118114 | 0.005018012 | ZFP36/CXCR2/CXCR1 | 3 |
| MF | GO:0004497 | monooxygenase activity | 5/109 | 105/18522 | 0.000389515 | 0.006173807 | 0.00506369 | CYP27B1/CYP2B6/CYP4A11/FMO3/MOXD1 | 5 |
| MF | GO:0005507 | copper ion binding | 4/109 | 63/18522 | 0.000517537 | 0.00781234 | 0.006407598 | ALB/S100A12/LOX/MOXD1 | 4 |
| MF | GO:0016705 | oxidoreductase activity, acting on paired donors, with incorporation or reduction of molecular oxygen | 6/109 | 179/18522 | 0.000666399 | 0.009602201 | 0.007875622 | PTGS2/CYP27B1/CYP2B6/CYP4A11/FMO3/MOXD1 | 6 |
| MF | GO:0019956 | chemokine binding | 3/109 | 33/18522 | 0.000951185 | 0.012566485 | 0.010306896 | ZFP36/CXCR2/CXCR1 | 3 |
| MF | GO:0042379 | chemokine receptor binding | 4/109 | 74/18522 | 0.000951406 | 0.012566485 | 0.010306896 | CCL2/CCL18/CCL19/CCL21 | 4 |
| MF | GO:0016614 | oxidoreductase activity, acting on CH-OH group of donors | 5/109 | 135/18522 | 0.00121685 | 0.014983486 | 0.012289295 | HAO2/HPGD/DCXR/HSD17B14/ADH1B | 5 |
| MF | GO:0043394 | proteoglycan binding | 3/109 | 36/18522 | 0.001228929 | 0.014983486 | 0.012289295 | LPL/TNC/FN1 | 3 |
| MF | GO:0016709 | oxidoreductase activity, acting on paired donors, with incorporation or reduction of molecular oxygen, NAD(P)H as one donor, and incorporation of one atom of oxygen | 3/109 | 38/18522 | 0.00143966 | 0.016902672 | 0.01386339 | CYP27B1/CYP2B6/FMO3 | 3 |
| MF | GO:0020037 | heme binding | 5/109 | 142/18522 | 0.001522498 | 0.017236854 | 0.014137483 | PTGS2/HBB/CYP27B1/CYP2B6/CYP4A11 | 5 |
| MF | GO:0001664 | G protein-coupled receptor binding | 7/109 | 291/18522 | 0.001673632 | 0.018294531 | 0.015004978 | FPR1/MAGI2/CCL2/CCL18/CCL19/CCL21/C3 | 7 |
| MF | GO:0001851 | complement component C3b binding | 2/109 | 11/18522 | 0.001823071 | 0.018642367 | 0.01529027 | CR1/VSIG4 | 2 |
| MF | GO:0019864 | IgG binding | 2/109 | 11/18522 | 0.001823071 | 0.018642367 | 0.01529027 | UMOD/FCGR3B | 2 |
| MF | GO:0046906 | tetrapyrrole binding | 5/109 | 152/18522 | 0.002052581 | 0.020333382 | 0.016677222 | PTGS2/HBB/CYP27B1/CYP2B6/CYP4A11 | 5 |
| MF | GO:0004857 | enzyme inhibitor activity | 8/109 | 389/18522 | 0.002117358 | 0.020339471 | 0.016682216 | RGS2/PRKAR2B/KNG1/PTN/SERPINF1/APOC1/COL6A3/C3 | 8 |
| MF | GO:0061134 | peptidase regulator activity | 6/109 | 232/18522 | 0.00250848 | 0.023237469 | 0.019059122 | KNG1/PCOLCE2/SERPINF1/COL6A3/C3/FN1 | 6 |
| MF | GO:0035325 | Toll-like receptor binding | 2/109 | 13/18522 | 0.002565651 | 0.023237469 | 0.019059122 | S100A9/S100A8 | 2 |
| MF | GO:0008392 | arachidonic acid epoxygenase activity | 2/109 | 16/18522 | 0.003901963 | 0.034358954 | 0.028180846 | CYP2B6/CYP4A11 | 2 |
| MF | GO:0004601 | peroxidase activity | 3/109 | 57/18522 | 0.004605662 | 0.039459317 | 0.032364108 | PTGS2/GSTA1/HBB | 3 |
| MF | GO:0008391 | arachidonic acid monooxygenase activity | 2/109 | 18/18522 | 0.004936999 | 0.041184969 | 0.03377947 | CYP2B6/CYP4A11 | 2 |
| MF | GO:0016684 | oxidoreductase activity, acting on peroxide as acceptor | 3/109 | 59/18522 | 0.005073834 | 0.04124116 | 0.033825557 | PTGS2/GSTA1/HBB | 3 |
| MF | GO:0030246 | carbohydrate binding | 6/109 | 279/18522 | 0.006151662 | 0.048751922 | 0.039985803 | CHI3L1/ALDOB/FBP1/MRC1/PTN/VCAN | 6 |
| MF | GO:0016616 | oxidoreductase activity, acting on the CH-OH group of donors, NAD or NADP as acceptor | 4/109 | 125/18522 | 0.006376961 | 0.049304794 | 0.040439263 | HPGD/DCXR/HSD17B14/ADH1B | 4 |
| MF | GO:0001846 | opsonin binding | 2/109 | 21/18522 | 0.006698865 | 0.050560478 | 0.041469161 | CR1/VSIG4 | 2 |
| MF | GO:0016493 | C-C chemokine receptor activity | 2/109 | 23/18522 | 0.008009026 | 0.059043286 | 0.04842667 | CXCR2/CXCR1 | 2 |
| MF | GO:0016702 | oxidoreductase activity, acting on single donors with incorporation of molecular oxygen, incorporation of two atoms of oxygen | 2/109 | 24/18522 | 0.008703783 | 0.0627068 | 0.051431445 | HPD/PTGS2 | 2 |
| MF | GO:0016701 | oxidoreductase activity, acting on single donors with incorporation of molecular oxygen | 2/109 | 25/18522 | 0.009424553 | 0.064484217 | 0.052889264 | HPD/PTGS2 | 2 |
| MF | GO:0048029 | monosaccharide binding | 3/109 | 74/18522 | 0.009494963 | 0.064484217 | 0.052889264 | ALDOB/FBP1/MRC1 | 3 |
| MF | GO:0001637 | G protein-coupled chemoattractant receptor activity | 2/109 | 26/18522 | 0.010171012 | 0.064484217 | 0.052889264 | CXCR2/CXCR1 | 2 |
| MF | GO:0001848 | complement binding | 2/109 | 26/18522 | 0.010171012 | 0.064484217 | 0.052889264 | CR1/VSIG4 | 2 |
| MF | GO:0004950 | chemokine receptor activity | 2/109 | 26/18522 | 0.010171012 | 0.064484217 | 0.052889264 | CXCR2/CXCR1 | 2 |
| MF | GO:0019865 | immunoglobulin binding | 2/109 | 26/18522 | 0.010171012 | 0.064484217 | 0.052889264 | UMOD/FCGR3B | 2 |
| MF | GO:0046332 | SMAD binding | 3/109 | 78/18522 | 0.010956472 | 0.068101992 | 0.055856523 | FOS/MAGI2/COL1A2 | 3 |
| MF | GO:0048306 | calcium-dependent protein binding | 3/109 | 79/18522 | 0.011341014 | 0.069136566 | 0.05670507 | S100A12/S100A9/S100A8 | 3 |
| MF | GO:0140272 | exogenous protein binding | 3/109 | 80/18522 | 0.011733277 | 0.070178277 | 0.05755947 | ALB/CR1/MRC1 | 3 |
| MF | GO:0004181 | metallocarboxypeptidase activity | 2/109 | 29/18522 | 0.012561321 | 0.073739609 | 0.060480436 | AEBP1/CPA3 | 2 |
| MF | GO:0008083 | growth factor activity | 4/109 | 162/18522 | 0.015433291 | 0.088951877 | 0.072957376 | IGF1/EGF/FGF9/PTN | 4 |
| MF | GO:0019825 | oxygen binding | 2/109 | 34/18522 | 0.017029487 | 0.096399061 | 0.079065476 | ALB/HBB | 2 |
| MF | GO:0004866 | endopeptidase inhibitor activity | 4/109 | 169/18522 | 0.017748624 | 0.098707258 | 0.080958635 | KNG1/SERPINF1/COL6A3/C3 | 4 |
| MF | GO:0030414 | peptidase inhibitor activity | 4/109 | 176/18522 | 0.020269902 | 0.110785501 | 0.090865079 | KNG1/SERPINF1/COL6A3/C3 | 4 |
| MF | GO:0001228 | DNA-binding transcription activator activity, RNA polymerase II-specific | 7/109 | 476/18522 | 0.0224159 | 0.120437974 | 0.098781934 | FOS/EGR1/JUNB/TCF21/NR4A2/FOSB/ATF3 | 7 |
| MF | GO:0008237 | metallopeptidase activity | 4/109 | 183/18522 | 0.023001631 | 0.121525283 | 0.099673733 | AEBP1/MMP2/CPA3/MMP7 | 4 |
| MF | GO:0001216 | DNA-binding transcription activator activity | 7/109 | 482/18522 | 0.023800755 | 0.12368589 | 0.10144584 | FOS/EGR1/JUNB/TCF21/NR4A2/FOSB/ATF3 | 7 |
| MF | GO:0061135 | endopeptidase regulator activity | 4/109 | 186/18522 | 0.024237765 | 0.123925346 | 0.10164224 | KNG1/SERPINF1/COL6A3/C3 | 4 |
| MF | GO:0016712 | oxidoreductase activity, acting on paired donors, with incorporation or reduction of molecular oxygen, reduced flavin or flavoprotein as one donor, and incorporation of one atom of oxygen | 2/109 | 43/18522 | 0.026490357 | 0.133292749 | 0.109325283 | CYP2B6/CYP4A11 | 2 |
| MF | GO:0004180 | carboxypeptidase activity | 2/109 | 45/18522 | 0.028823471 | 0.14056985 | 0.115293883 | AEBP1/CPA3 | 2 |
| MF | GO:0030020 | extracellular matrix structural constituent conferring tensile strength | 2/109 | 45/18522 | 0.028823471 | 0.14056985 | 0.115293883 | COL6A3/COL1A2 | 2 |
| MF | GO:0016860 | intramolecular oxidoreductase activity | 2/109 | 49/18522 | 0.033724829 | 0.161981375 | 0.132855387 | GSTA1/PTGDS | 2 |
| MF | GO:0016830 | carbon-carbon lyase activity | 2/109 | 51/18522 | 0.03628883 | 0.171694911 | 0.140822324 | ALDOB/PCK1 | 2 |
| MF | GO:0005179 | hormone activity | 3/109 | 126/18522 | 0.038335573 | 0.178711419 | 0.146577189 | IGF1/KNG1/SST | 3 |
| MF | GO:0016504 | peptidase activator activity | 2/109 | 55/18522 | 0.041633151 | 0.191271145 | 0.156878542 | PCOLCE2/FN1 | 2 |
| MF | GO:0070851 | growth factor receptor binding | 3/109 | 138/18522 | 0.048015888 | 0.210248315 | 0.172443413 | ESM1/EGF/FGF9 | 3 |

### **2.3 Table S3: KEGG enrichment analysis results.**

| ID | Description | | GeneRatio | BgRatio | | | pvalue | p.adjust | | qvalue | | geneID | Count | |
| --- | --- | --- | --- | --- | --- | --- | --- | --- | --- | --- | --- | --- | --- | --- |
| hsa04657 | IL-17 signaling pathway | 6/75 | | | 95/8842 | 0.000145473 | | 0.013178512 | 0.010511347 | | FOS/S100A9/PTGS2/S100A8/FOSB/CCL2 | | 6 |  |
| hsa04061 | Viral protein interaction with cytokine and cytokine receptor | 6/75 | | | 100/8842 | 0.00019297 | | 0.013178512 | 0.010511347 | | CXCR2/CXCR1/CCL2/CCL18/CCL19/CCL21 | | 6 |  |
| hsa04933 | AGE-RAGE signaling pathway in diabetic complications | 6/75 | | | 101/8842 | 0.000203791 | | 0.013178512 | 0.010511347 | | EGR1/PLCG2/MMP2/CCL2/COL1A2/FN1 | | 6 |  |
| hsa05140 | Leishmaniasis | 5/75 | | | 77/8842 | 0.000471341 | | 0.022192458 | 0.017700984 | | FOS/PTGS2/CR1/FCGR3B/C3 | | 5 |  |
| hsa05144 | Malaria | 4/75 | | | 50/8842 | 0.000818578 | | 0.022192458 | 0.017700984 | | HBB/CR1/THBS2/CCL2 | | 4 |  |
| hsa04610 | Complement and coagulation cascades | 5/75 | | | 88/8842 | 0.000869428 | | 0.022192458 | 0.017700984 | | KNG1/CR1/VSIG4/C7/C3 | | 5 |  |
| hsa04151 | PI3K-Akt signaling pathway | 10/75 | | | 362/8842 | 0.000900746 | | 0.022192458 | 0.017700984 | | IGF1/EGF/PCK1/MAGI2/FGF9/TNC/THBS2/COL6A3/COL1A2/FN1 | | 10 |  |
| hsa04512 | ECM-receptor interaction | 5/75 | | | 89/8842 | 0.000915153 | | 0.022192458 | 0.017700984 | | TNC/THBS2/COL6A3/COL1A2/FN1 | | 5 |  |
| hsa04062 | Chemokine signaling pathway | 7/75 | | | 193/8842 | 0.00119969 | | 0.025859991 | 0.020626255 | | CXCR2/CXCR1/PLCG2/CCL2/CCL18/CCL19/CCL21 | | 7 |  |
| hsa05150 | Staphylococcus aureus infection | 5/75 | | | 100/8842 | 0.001544383 | | 0.027953162 | 0.022295794 | | FPR1/FCGR3B/KRT19/FPR3/C3 | | 5 |  |
| hsa04510 | Focal adhesion | 7/75 | | | 203/8842 | 0.001606242 | | 0.027953162 | 0.022295794 | | IGF1/EGF/TNC/THBS2/COL6A3/COL1A2/FN1 | | 7 |  |
| hsa00590 | Arachidonic acid metabolism | 4/75 | | | 61/8842 | 0.001729062 | | 0.027953162 | 0.022295794 | | PTGS2/HPGD/PTGDS/CYP2B6 | | 4 |  |
| hsa00010 | Glycolysis / Gluconeogenesis | 4/75 | | | 67/8842 | 0.002444281 | | 0.036476196 | 0.029093873 | | ALDOB/FBP1/PCK1/ADH1B | | 4 |  |
| hsa04928 | Parathyroid hormone synthesis, secretion and action | 5/75 | | | 115/8842 | 0.002853756 | | 0.039544906 | 0.031541514 | | FOS/EGR1/SLC12A3/NR4A2/CYP27B1 | | 5 |  |
| hsa00982 | Drug metabolism - cytochrome P450 | 4/75 | | | 73/8842 | 0.003340885 | | 0.041355892 | 0.03298598 | | GSTA1/CYP2B6/FMO3/ADH1B | | 4 |  |
| hsa04820 | Cytoskeleton in muscle cells | 7/75 | | | 232/8842 | 0.003410795 | | 0.041355892 | 0.03298598 | | TNNI1/TNNT2/THBS2/COL6A3/COL1A2/VCAN/FN1 | | 7 |  |
| hsa04935 | Growth hormone synthesis, secretion and action | 5/75 | | | 122/8842 | 0.003681909 | | 0.042017082 | 0.033513354 | | FOS/JUNB/IGF1/SST/PLCG2 | | 5 |  |
| hsa04613 | Neutrophil extracellular trap formation | 6/75 | | | 192/8842 | 0.005640401 | | 0.060790989 | 0.048487658 | | CR1/FPR1/FCGR3B/PLCG2/FPR3/C3 | | 6 |  |
| hsa05418 | Fluid shear stress and atherosclerosis | 5/75 | | | 141/8842 | 0.006783044 | | 0.067753808 | 0.05404129 | | FOS/GSTA1/DUSP1/MMP2/CCL2 | | 5 |  |
| hsa04380 | Osteoclast differentiation | 5/75 | | | 142/8842 | 0.006984929 | | 0.067753808 | 0.05404129 | | FOS/JUNB/FOSB/FCGR3B/PLCG2 | | 5 |  |
| hsa05205 | Proteoglycans in cancer | 6/75 | | | 204/8842 | 0.007529401 | | 0.069557324 | 0.055479797 | | IGF1/PLCG2/MMP2/COL1A2/LUM/FN1 | | 6 |  |
| hsa04979 | Cholesterol metabolism | 3/75 | | | 51/8842 | 0.009118991 | | 0.080412918 | 0.064138356 | | LPL/APOH/APOC1 | | 3 |  |
| hsa05142 | Chagas disease | 4/75 | | | 103/8842 | 0.011219836 | | 0.094636879 | 0.075483566 | | FOS/KNG1/CCL2/C3 | | 4 |  |
| hsa04064 | NF-kappa B signaling pathway | 4/75 | | | 105/8842 | 0.011978419 | | 0.096825556 | 0.077229282 | | PTGS2/PLCG2/CCL19/CCL21 | | 4 |  |
| hsa04066 | HIF-1 signaling pathway | 4/75 | | | 109/8842 | 0.013592849 | | 0.105480511 | 0.084132583 | | IGF1/EGF/ALDOB/PLCG2 | | 4 |  |
| hsa05152 | Tuberculosis | 5/75 | | | 180/8842 | 0.01813113 | | 0.130889416 | 0.104399046 | | CR1/CYP27B1/FCGR3B/MRC1/C3 | | 5 |  |
| hsa04668 | TNF signaling pathway | 4/75 | | | 119/8842 | 0.018216568 | | 0.130889416 | 0.104399046 | | FOS/PTGS2/JUNB/CCL2 | | 4 |  |
| hsa05120 | Epithelial cell signaling in Helicobacter pylori infection | 3/75 | | | 71/8842 | 0.022193908 | | 0.149777447 | 0.119464377 | | CXCR2/CXCR1/PLCG2 | | 3 |  |
| hsa05165 | Human papillomavirus infection | 7/75 | | | 333/8842 | 0.022389412 | | 0.149777447 | 0.119464377 | | PTGS2/EGF/TNC/THBS2/COL6A3/COL1A2/FN1 | | 7 |  |
| hsa05218 | Melanoma | 3/75 | | | 73/8842 | 0.023864107 | | 0.154321228 | 0.123088554 | | IGF1/EGF/FGF9 | | 3 |  |
| hsa05167 | Kaposi sarcoma-associated herpesvirus infection | 5/75 | | | 196/8842 | 0.025141144 | | 0.155753591 | 0.124231025 | | FOS/ZFP36/PTGS2/PLCG2/C3 | | 5 |  |
| hsa03320 | PPAR signaling pathway | 3/75 | | | 76/8842 | 0.026494168 | | 0.155753591 | 0.124231025 | | LPL/PCK1/FABP1 | | 3 |  |
| hsa05214 | Glioma | 3/75 | | | 76/8842 | 0.026494168 | | 0.155753591 | 0.124231025 | | IGF1/EGF/PLCG2 | | 3 |  |
| hsa00030 | Pentose phosphate pathway | 2/75 | | | 31/8842 | 0.028168764 | | 0.160727652 | 0.128198398 | | ALDOB/FBP1 | | 2 |  |
| hsa00980 | Metabolism of xenobiotics by cytochrome P450 | 3/75 | | | 79/8842 | 0.029273678 | | 0.162259816 | 0.129420472 | | GSTA1/CYP2B6/ADH1B | | 3 |  |
| hsa01521 | EGFR tyrosine kinase inhibitor resistance | 3/75 | | | 80/8842 | 0.030233304 | | 0.162923917 | 0.129950167 | | IGF1/EGF/PLCG2 | | 3 |  |
| hsa00051 | Fructose and mannose metabolism | 2/75 | | | 34/8842 | 0.033436418 | | 0.172053096 | 0.137231715 | | ALDOB/FBP1 | | 2 |  |
| hsa04015 | Rap1 signaling pathway | 5/75 | | | 212/8842 | 0.033701122 | | 0.172053096 | 0.137231715 | | IGF1/EGF/FPR1/MAGI2/FGF9 | | 5 |  |
| hsa05207 | Chemical carcinogenesis - receptor activation | 5/75 | | | 215/8842 | 0.035485783 | | 0.173115477 | 0.138079083 | | FOS/GSTA1/EGF/CYP2B6/FGF9 | | 5 |  |
| hsa05224 | Breast cancer | 4/75 | | | 148/8842 | 0.036701132 | | 0.173115477 | 0.138079083 | | FOS/IGF1/EGF/FGF9 | | 4 |  |
| hsa00350 | Tyrosine metabolism | 2/75 | | | 36/8842 | 0.037144952 | | 0.173115477 | 0.138079083 | | HPD/ADH1B | | 2 |  |
| hsa04072 | Phospholipase D signaling pathway | 4/75 | | | 149/8842 | 0.037478608 | | 0.173115477 | 0.138079083 | | CXCR2/EGF/CXCR1/PLCG2 | | 4 |  |
| hsa05143 | African trypanosomiasis | 2/75 | | | 37/8842 | 0.039055929 | | 0.176205819 | 0.140543979 | | HBB/KNG1 | | 2 |  |
| hsa04060 | Cytokine-cytokine receptor interaction | 6/75 | | | 298/8842 | 0.040302142 | | 0.177695806 | 0.141732412 | | CXCR2/CXCR1/CCL2/CCL18/CCL19/CCL21 | | 6 |  |
| hsa04145 | Phagosome | 4/75 | | | 157/8842 | 0.044040619 | | 0.189864001 | 0.151437917 | | FCGR3B/MRC1/THBS2/C3 | | 4 |  |
| hsa04810 | Regulation of actin cytoskeleton | 5/75 | | | 230/8842 | 0.045283575 | | 0.190978557 | 0.152326901 | | EGF/KNG1/FGF9/C7/FN1 | | 5 |  |
| hsa05219 | Bladder cancer | 2/75 | | | 41/8842 | 0.047060443 | | 0.19424949 | 0.154935838 | | EGF/MMP2 | | 2 |  |
| hsa04666 | Fc gamma R-mediated phagocytosis | 3/75 | | | 98/8842 | 0.050289758 | | 0.19611171 | 0.156421169 | | FCGR3B/PLCG2/MARCKS | | 3 |  |
| hsa05171 | Coronavirus disease - COVID-19 | 5/75 | | | 238/8842 | 0.051113124 | | 0.19611171 | 0.156421169 | | FOS/PLCG2/CCL2/C7/C3 | | 5 |  |
| hsa01522 | Endocrine resistance | 3/75 | | | 99/8842 | 0.05155514 | | 0.19611171 | 0.156421169 | | FOS/IGF1/MMP2 | | 3 |  |
| hsa04750 | Inflammatory mediator regulation of TRP channels | 3/75 | | | 99/8842 | 0.05155514 | | 0.19611171 | 0.156421169 | | IGF1/KNG1/PLCG2 | | 3 |  |
| hsa04974 | Protein digestion and absorption | 3/75 | | | 105/8842 | 0.059469129 | | 0.219879349 | 0.175378537 | | CPA3/COL6A3/COL1A2 | | 3 |  |
| hsa00620 | Pyruvate metabolism | 2/75 | | | 47/8842 | 0.060070132 | | 0.219879349 | 0.175378537 | | PCK1/ADH1B | | 2 |  |
| hsa00330 | Arginine and proline metabolism | 2/75 | | | 50/8842 | 0.066986216 | | 0.240654183 | 0.191948806 | | AGMAT/PRODH2 | | 2 |  |
| hsa04913 | Ovarian steroidogenesis | 2/75 | | | 51/8842 | 0.069348144 | | 0.244609815 | 0.195103868 | | PTGS2/IGF1 | | 2 |  |
| hsa01200 | Carbon metabolism | 3/75 | | | 116/8842 | 0.075360648 | | 0.261070816 | 0.208233369 | | HAO2/ALDOB/FBP1 | | 3 |  |
| hsa05134 | Legionellosis | 2/75 | | | 56/8842 | 0.08155405 | | 0.277569925 | 0.221393266 | | CR1/C3 | | 2 |  |
| hsa04152 | AMPK signaling pathway | 3/75 | | | 122/8842 | 0.084744547 | | 0.283455899 | 0.226087993 | | IGF1/FBP1/PCK1 | | 3 |  |
| hsa00130 | Ubiquinone and other terpenoid-quinone biosynthesis | 1/75 | | | 11/8842 | 0.089495017 | | 0.29427175 | 0.234714852 | | HPD | | 1 |  |
| hsa04370 | VEGF signaling pathway | 2/75 | | | 60/8842 | 0.091758593 | | 0.296686117 | 0.236640582 | | PTGS2/PLCG2 | | 2 |  |
| hsa04926 | Relaxin signaling pathway | 3/75 | | | 130/8842 | 0.097992129 | | 0.311647098 | 0.248573648 | | FOS/MMP2/COL1A2 | | 3 |  |
| hsa04068 | FoxO signaling pathway | 3/75 | | | 133/8842 | 0.10316604 | | 0.322809866 | 0.257477212 | | IGF1/EGF/PCK1 | | 3 |  |
| hsa04010 | MAPK signaling pathway | 5/75 | | | 300/8842 | 0.110528371 | | 0.324758596 | 0.259031545 | | FOS/DUSP1/IGF1/EGF/FGF9 | | 5 |  |
| hsa04910 | Insulin signaling pathway | 3/75 | | | 138/8842 | 0.112026363 | | 0.324758596 | 0.259031545 | | PRKAR2B/FBP1/PCK1 | | 3 |  |
| hsa05135 | Yersinia infection | 3/75 | | | 138/8842 | 0.112026363 | | 0.324758596 | 0.259031545 | | FOS/CCL2/FN1 | | 3 |  |
| hsa00830 | Retinol metabolism | 2/75 | | | 68/8842 | 0.113188225 | | 0.324758596 | 0.259031545 | | CYP2B6/ADH1B | | 2 |  |
| hsa04915 | Estrogen signaling pathway | 3/75 | | | 139/8842 | 0.11383291 | | 0.324758596 | 0.259031545 | | FOS/KRT19/MMP2 | | 3 |  |
| hsa05322 | Systemic lupus erythematosus | 3/75 | | | 139/8842 | 0.11383291 | | 0.324758596 | 0.259031545 | | FCGR3B/C7/C3 | | 3 |  |
| hsa05031 | Amphetamine addiction | 2/75 | | | 69/8842 | 0.115952343 | | 0.326010937 | 0.260030427 | | FOS/FOSB | | 2 |  |
| hsa05166 | Human T-cell leukemia virus 1 infection | 4/75 | | | 223/8842 | 0.120601973 | | 0.329841478 | 0.263085715 | | FOS/ZFP36/EGR1/MMP7 | | 4 |  |
| hsa05204 | Chemical carcinogenesis - DNA adducts | 2/75 | | | 71/8842 | 0.121532029 | | 0.329841478 | 0.263085715 | | PTGS2/GSTA1 | | 2 |  |
| hsa04936 | Alcoholic liver disease | 3/75 | | | 144/8842 | 0.12303132 | | 0.329841478 | 0.263085715 | | FABP1/ADH1B/C3 | | 3 |  |
| hsa05223 | Non-small cell lung cancer | 2/75 | | | 73/8842 | 0.127177133 | | 0.329841478 | 0.263085715 | | EGF/PLCG2 | | 2 |  |
| hsa00360 | Phenylalanine metabolism | 1/75 | | | 16/8842 | 0.127516035 | | 0.329841478 | 0.263085715 | | HPD | | 1 |  |
| hsa00430 | Taurine and hypotaurine metabolism | 1/75 | | | 16/8842 | 0.127516035 | | 0.329841478 | 0.263085715 | | FMO3 | | 1 |  |
| hsa05133 | Pertussis | 2/75 | | | 78/8842 | 0.141551913 | | 0.355738858 | 0.283741792 | | FOS/C3 | | 2 |  |
| hsa04921 | Oxytocin signaling pathway | 3/75 | | | 154/8842 | 0.142206868 | | 0.355738858 | 0.283741792 | | FOS/PTGS2/RGS2 | | 3 |  |
| hsa04014 | Ras signaling pathway | 4/75 | | | 238/8842 | 0.143029026 | | 0.355738858 | 0.283741792 | | IGF1/EGF/PLCG2/FGF9 | | 4 |  |
| hsa00100 | Steroid biosynthesis | 1/75 | | | 20/8842 | 0.156801227 | | 0.385056178 | 0.307125655 | | CYP27B1 | | 1 |  |
| hsa04012 | ErbB signaling pathway | 2/75 | | | 86/8842 | 0.165217375 | | 0.400652133 | 0.319565185 | | EGF/PLCG2 | | 2 |  |
| hsa05210 | Colorectal cancer | 2/75 | | | 87/8842 | 0.168224578 | | 0.402908248 | 0.32136469 | | FOS/EGF | | 2 |  |
| hsa05235 | PD-L1 expression and PD-1 checkpoint pathway in cancer | 2/75 | | | 90/8842 | 0.177302313 | | 0.411387651 | 0.328127969 | | FOS/EGF | | 2 |  |
| hsa04614 | Renin-angiotensin system | 1/75 | | | 23/8842 | 0.178126612 | | 0.411387651 | 0.328127969 | | CPA3 | | 1 |  |
| hsa04964 | Proximal tubule bicarbonate reclamation | 1/75 | | | 23/8842 | 0.178126612 | | 0.411387651 | 0.328127969 | | PCK1 | | 1 |  |
| hsa04662 | B cell receptor signaling pathway | 2/75 | | | 91/8842 | 0.180345712 | | 0.411612566 | 0.328307364 | | FOS/PLCG2 | | 2 |  |
| hsa04912 | GnRH signaling pathway | 2/75 | | | 93/8842 | 0.186456595 | | 0.415776776 | 0.33162879 | | EGR1/MMP2 | | 2 |  |
| hsa05222 | Small cell lung cancer | 2/75 | | | 93/8842 | 0.186456595 | | 0.415776776 | 0.33162879 | | PTGS2/FN1 | | 2 |  |
| hsa05323 | Rheumatoid arthritis | 2/75 | | | 94/8842 | 0.189523384 | | 0.417812916 | 0.333252841 | | FOS/CCL2 | | 2 |  |
| hsa04080 | Neuroactive ligand-receptor interaction | 5/75 | | | 368/8842 | 0.201530422 | | 0.432192657 | 0.344722304 | | KNG1/SST/FPR1/FPR3/C3 | | 5 |  |
| hsa05215 | Prostate cancer | 2/75 | | | 98/8842 | 0.201858308 | | 0.432192657 | 0.344722304 | | IGF1/EGF | | 2 |  |
| hsa05231 | Choline metabolism in cancer | 2/75 | | | 99/8842 | 0.204957343 | | 0.432192657 | 0.344722304 | | FOS/EGF | | 2 |  |
| hsa05410 | Hypertrophic cardiomyopathy | 2/75 | | | 99/8842 | 0.204957343 | | 0.432192657 | 0.344722304 | | IGF1/TNNT2 | | 2 |  |
| hsa00601 | Glycosphingolipid biosynthesis - lacto and neolacto series | 1/75 | | | 28/8842 | 0.212492452 | | 0.443263825 | 0.353552807 | | B3GALT2 | | 1 |  |
| hsa05146 | Amoebiasis | 2/75 | | | 103/8842 | 0.21740523 | | 0.44868739 | 0.35787871 | | COL1A2/FN1 | | 2 |  |
| hsa04625 | C-type lectin receptor signaling pathway | 2/75 | | | 105/8842 | 0.223655991 | | 0.451676577 | 0.360262923 | | PTGS2/PLCG2 | | 2 |  |
| hsa05414 | Dilated cardiomyopathy | 2/75 | | | 105/8842 | 0.223655991 | | 0.451676577 | 0.360262923 | | IGF1/TNNT2 | | 2 |  |
| hsa00020 | Citrate cycle (TCA cycle) | 1/75 | | | 30/8842 | 0.225838288 | | 0.451676577 | 0.360262923 | | PCK1 | | 1 |  |
| hsa04922 | Glucagon signaling pathway | 2/75 | | | 107/8842 | 0.229921514 | | 0.455150752 | 0.36303397 | | FBP1/PCK1 | | 2 |  |
| hsa00630 | Glyoxylate and dicarboxylate metabolism | 1/75 | | | 31/8842 | 0.232427272 | | 0.455463544 | 0.363283456 | | HAO2 | | 1 |  |
| hsa05415 | Diabetic cardiomyopathy | 3/75 | | | 205/8842 | 0.251908663 | | 0.488702806 | 0.38979551 | | PDK4/MMP2/COL1A2 | | 3 |  |
| hsa04726 | Serotonergic synapse | 2/75 | | | 115/8842 | 0.255087342 | | 0.489969747 | 0.390806038 | | PTGS2/DUSP1 | | 2 |  |
| hsa04670 | Leukocyte transendothelial migration | 2/75 | | | 116/8842 | 0.258240442 | | 0.491163194 | 0.391757947 | | PLCG2/MMP2 | | 2 |  |
| hsa00040 | Pentose and glucuronate interconversions | 1/75 | | | 36/8842 | 0.264551235 | | 0.498280967 | 0.397435172 | | DCXR | | 1 |  |
| hsa05206 | MicroRNAs in cancer | 4/75 | | | 312/8842 | 0.272340962 | | 0.507339139 | 0.404660083 | | PTGS2/PLCG2/TNC/MARCKS | | 4 |  |
| hsa04960 | Aldosterone-regulated sodium reabsorption | 1/75 | | | 38/8842 | 0.277026109 | | 0.507339139 | 0.404660083 | | IGF1 | | 1 |  |
| hsa05417 | Lipid and atherosclerosis | 3/75 | | | 216/8842 | 0.277205921 | | 0.507339139 | 0.404660083 | | FOS/CYP2B6/CCL2 | | 3 |  |
| hsa04611 | Platelet activation | 2/75 | | | 125/8842 | 0.286627146 | | 0.519679125 | 0.414502612 | | PLCG2/COL1A2 | | 2 |  |
| hsa00260 | Glycine, serine and threonine metabolism | 1/75 | | | 41/8842 | 0.295347949 | | 0.529851342 | 0.4226161 | | BHMT | | 1 |  |
| hsa05163 | Human cytomegalovirus infection | 3/75 | | | 226/8842 | 0.300431173 | | 0.529851342 | 0.4226161 | | PTGS2/CXCR2/CCL2 | | 3 |  |
| hsa05208 | Chemical carcinogenesis - reactive oxygen species | 3/75 | | | 226/8842 | 0.300431173 | | 0.529851342 | 0.4226161 | | FOS/GSTA1/EGF | | 3 |  |
| hsa00071 | Fatty acid degradation | 1/75 | | | 43/8842 | 0.307307203 | | 0.532299976 | 0.424569162 | | ADH1B | | 1 |  |
| hsa04975 | Fat digestion and absorption | 1/75 | | | 43/8842 | 0.307307203 | | 0.532299976 | 0.424569162 | | FABP1 | | 1 |  |
| hsa04650 | Natural killer cell mediated cytotoxicity | 2/75 | | | 135/8842 | 0.318050856 | | 0.546034213 | 0.435523762 | | FCGR3B/PLCG2 | | 2 |  |
| hsa05030 | Cocaine addiction | 1/75 | | | 49/8842 | 0.341996294 | | 0.581993694 | 0.464205496 | | FOSB | | 1 |  |
| hsa05110 | Vibrio cholerae infection | 1/75 | | | 51/8842 | 0.353173957 | | 0.591372352 | 0.471686032 | | PLCG2 | | 1 |  |
| hsa00270 | Cysteine and methionine metabolism | 1/75 | | | 52/8842 | 0.358692323 | | 0.591372352 | 0.471686032 | | BHMT | | 1 |  |
| hsa04144 | Endocytosis | 3/75 | | | 252/8842 | 0.361138815 | | 0.591372352 | 0.471686032 | | EHD3/CXCR2/CXCR1 | | 3 |  |
| hsa04961 | Endocrine and other factor-regulated calcium reabsorption | 1/75 | | | 53/8842 | 0.364164231 | | 0.591372352 | 0.471686032 | | CALB1 | | 1 |  |
| hsa05226 | Gastric cancer | 2/75 | | | 150/8842 | 0.364562834 | | 0.591372352 | 0.471686032 | | EGF/FGF9 | | 2 |  |
| hsa04020 | Calcium signaling pathway | 3/75 | | | 254/8842 | 0.365797331 | | 0.591372352 | 0.471686032 | | EGF/PLCG2/FGF9 | | 3 |  |
| hsa00480 | Glutathione metabolism | 1/75 | | | 58/8842 | 0.39084031 | | 0.624650772 | 0.498229319 | | GSTA1 | | 1 |  |
| hsa04923 | Regulation of lipolysis in adipocytes | 1/75 | | | 59/8842 | 0.396041469 | | 0.624650772 | 0.498229319 | | PTGS2 | | 1 |  |
| hsa05213 | Endometrial cancer | 1/75 | | | 59/8842 | 0.396041469 | | 0.624650772 | 0.498229319 | | EGF | | 1 |  |
| hsa04730 | Long-term depression | 1/75 | | | 60/8842 | 0.401198806 | | 0.627682003 | 0.500647067 | | IGF1 | | 1 |  |
| hsa04213 | Longevity regulating pathway - multiple species | 1/75 | | | 62/8842 | 0.411383469 | | 0.635658841 | 0.507009493 | | IGF1 | | 1 |  |
| hsa04022 | cGMP-PKG signaling pathway | 2/75 | | | 166/8842 | 0.412850587 | | 0.635658841 | 0.507009493 | | RGS2/KNG1 | | 2 |  |
| hsa05225 | Hepatocellular carcinoma | 2/75 | | | 170/8842 | 0.424653522 | | 0.646172791 | 0.515395552 | | GSTA1/PLCG2 | | 2 |  |
| hsa00561 | Glycerolipid metabolism | 1/75 | | | 65/8842 | 0.42634081 | | 0.646172791 | 0.515395552 | | LPL | | 1 |  |
| hsa04310 | Wnt signaling pathway | 2/75 | | | 174/8842 | 0.436336531 | | 0.656196023 | 0.523390208 | | SERPINF1/MMP7 | | 2 |  |
| hsa04664 | Fc epsilon RI signaling pathway | 1/75 | | | 69/8842 | 0.445701992 | | 0.665124511 | 0.530511683 | | PLCG2 | | 1 |  |
| hsa04920 | Adipocytokine signaling pathway | 1/75 | | | 70/8842 | 0.450440662 | | 0.667064797 | 0.532059279 | | PCK1 | | 1 |  |
| hsa04917 | Prolactin signaling pathway | 1/75 | | | 71/8842 | 0.455139356 | | 0.668916933 | 0.533536566 | | FOS | | 1 |  |
| hsa00562 | Inositol phosphate metabolism | 1/75 | | | 73/8842 | 0.464418149 | | 0.670561705 | 0.534848457 | | PLCG2 | | 1 |  |
| hsa01230 | Biosynthesis of amino acids | 1/75 | | | 75/8842 | 0.473540998 | | 0.670561705 | 0.534848457 | | ALDOB | | 1 |  |
| hsa01524 | Platinum drug resistance | 1/75 | | | 75/8842 | 0.473540998 | | 0.670561705 | 0.534848457 | | GSTA1 | | 1 |  |
| hsa04115 | p53 signaling pathway | 1/75 | | | 75/8842 | 0.473540998 | | 0.670561705 | 0.534848457 | | IGF1 | | 1 |  |
| hsa04918 | Thyroid hormone synthesis | 1/75 | | | 75/8842 | 0.473540998 | | 0.670561705 | 0.534848457 | | ALB | | 1 |  |
| hsa04971 | Gastric acid secretion | 1/75 | | | 76/8842 | 0.478044753 | | 0.672033929 | 0.536022721 | | SST | | 1 |  |
| hsa05212 | Pancreatic cancer | 1/75 | | | 77/8842 | 0.48251049 | | 0.673431906 | 0.537137766 | | EGF | | 1 |  |
| hsa05100 | Bacterial invasion of epithelial cells | 1/75 | | | 78/8842 | 0.486938523 | | 0.674246234 | 0.537787284 | | FN1 | | 1 |  |
| hsa05202 | Transcriptional misregulation in cancer | 2/75 | | | 193/8842 | 0.490044943 | | 0.674246234 | 0.537787284 | | IGF1/HPGD | | 2 |  |
| hsa00983 | Drug metabolism - other enzymes | 1/75 | | | 81/8842 | 0.499999535 | | 0.679610926 | 0.54206623 | | GSTA1 | | 1 |  |
| hsa04814 | Motor proteins | 2/75 | | | 197/8842 | 0.500950322 | | 0.679610926 | 0.54206623 | | TNNI1/TNNT2 | | 2 |  |
| hsa04146 | Peroxisome | 1/75 | | | 83/8842 | 0.508524051 | | 0.685094902 | 0.546440318 | | HAO2 | | 1 |  |
| hsa04260 | Cardiac muscle contraction | 1/75 | | | 87/8842 | 0.525145177 | | 0.70260803 | 0.560409009 | | TNNT2 | | 1 |  |
| hsa04211 | Longevity regulating pathway | 1/75 | | | 90/8842 | 0.537245882 | | 0.709784107 | 0.566132739 | | IGF1 | | 1 |  |
| hsa05170 | Human immunodeficiency virus 1 infection | 2/75 | | | 213/8842 | 0.543079444 | | 0.709784107 | 0.566132739 | | FOS/PLCG2 | | 2 |  |
| hsa04540 | Gap junction | 1/75 | | | 92/8842 | 0.545143464 | | 0.709784107 | 0.566132739 | | EGF | | 1 |  |
| hsa04658 | Th1 and Th2 cell differentiation | 1/75 | | | 92/8842 | 0.545143464 | | 0.709784107 | 0.566132739 | | FOS | | 1 |  |
| hsa04713 | Circadian entrainment | 1/75 | | | 97/8842 | 0.564310294 | | 0.724966471 | 0.578242383 | | FOS | | 1 |  |
| hsa04070 | Phosphatidylinositol signaling system | 1/75 | | | 98/8842 | 0.568046912 | | 0.724966471 | 0.578242383 | | PLCG2 | | 1 |  |
| hsa04925 | Aldosterone synthesis and secretion | 1/75 | | | 98/8842 | 0.568046912 | | 0.724966471 | 0.578242383 | | NR4A2 | | 1 |  |
| hsa04640 | Hematopoietic cell lineage | 1/75 | | | 99/8842 | 0.571751908 | | 0.724966471 | 0.578242383 | | CR1 | | 1 |  |
| hsa04024 | cAMP signaling pathway | 2/75 | | | 226/8842 | 0.575490756 | | 0.724968875 | 0.578244301 | | FOS/SST | | 2 |  |
| hsa04972 | Pancreatic secretion | 1/75 | | | 106/8842 | 0.596823346 | | 0.745450691 | 0.594580855 | | CPA3 | | 1 |  |
| hsa04659 | Th17 cell differentiation | 1/75 | | | 108/8842 | 0.603716699 | | 0.745450691 | 0.594580855 | | FOS | | 1 |  |
| hsa04620 | Toll-like receptor signaling pathway | 1/75 | | | 109/8842 | 0.607119635 | | 0.745450691 | 0.594580855 | | FOS | | 1 |  |
| hsa04931 | Insulin resistance | 1/75 | | | 109/8842 | 0.607119635 | | 0.745450691 | 0.594580855 | | PCK1 | | 1 |  |
| hsa04914 | Progesterone-mediated oocyte maturation | 1/75 | | | 111/8842 | 0.613839244 | | 0.74896109 | 0.597380793 | | IGF1 | | 1 |  |
| hsa04725 | Cholinergic synapse | 1/75 | | | 115/8842 | 0.626940084 | | 0.757605303 | 0.604275526 | | FOS | | 1 |  |
| hsa05131 | Shigellosis | 2/75 | | | 249/8842 | 0.628734298 | | 0.757605303 | 0.604275526 | | PLCG2/C3 | | 2 |  |
| hsa04722 | Neurotrophin signaling pathway | 1/75 | | | 120/8842 | 0.64270088 | | 0.762854561 | 0.608462401 | | PLCG2 | | 1 |  |
| hsa04071 | Sphingolipid signaling pathway | 1/75 | | | 122/8842 | 0.648819601 | | 0.762854561 | 0.608462401 | | KNG1 | | 1 |  |
| hsa04660 | T cell receptor signaling pathway | 1/75 | | | 122/8842 | 0.648819601 | | 0.762854561 | 0.608462401 | | FOS | | 1 |  |
| hsa04919 | Thyroid hormone signaling pathway | 1/75 | | | 122/8842 | 0.648819601 | | 0.762854561 | 0.608462401 | | PLCG2 | | 1 |  |
| hsa04728 | Dopaminergic synapse | 1/75 | | | 132/8842 | 0.677896134 | | 0.792240061 | 0.631900645 | | FOS | | 1 |  |
| hsa05020 | Prion disease | 2/75 | | | 278/8842 | 0.688424288 | | 0.793511922 | 0.632915098 | | EGR1/C7 | | 2 |  |
| hsa04210 | Apoptosis | 1/75 | | | 136/8842 | 0.688849799 | | 0.793511922 | 0.632915098 | | FOS | | 1 |  |
| hsa04114 | Oocyte meiosis | 1/75 | | | 139/8842 | 0.696823073 | | 0.793511922 | 0.632915098 | | IGF1 | | 1 |  |
| hsa05162 | Measles | 1/75 | | | 139/8842 | 0.696823073 | | 0.793511922 | 0.632915098 | | FOS | | 1 |  |
| hsa04371 | Apelin signaling pathway | 1/75 | | | 140/8842 | 0.699435767 | | 0.793511922 | 0.632915098 | | EGR1 | | 1 |  |
| hsa04550 | Signaling pathways regulating pluripotency of stem cells | 1/75 | | | 144/8842 | 0.709666218 | | 0.800437479 | 0.638439009 | | IGF1 | | 1 |  |
| hsa04723 | Retrograde endocannabinoid signaling | 1/75 | | | 149/8842 | 0.721972253 | | 0.803009945 | 0.640490841 | | PTGS2 | | 1 |  |
| hsa01240 | Biosynthesis of cofactors | 1/75 | | | 153/8842 | 0.731445291 | | 0.803009945 | 0.640490841 | | HPD | | 1 |  |
| hsa04261 | Adrenergic signaling in cardiomyocytes | 1/75 | | | 154/8842 | 0.733763349 | | 0.803009945 | 0.640490841 | | TNNT2 | | 1 |  |
| hsa04148 | Efferocytosis | 1/75 | | | 156/8842 | 0.738340398 | | 0.803009945 | 0.640490841 | | PTGS2 | | 1 |  |
| hsa04514 | Cell adhesion molecules | 1/75 | | | 157/8842 | 0.74059972 | | 0.803009945 | 0.640490841 | | VCAN | | 1 |  |
| hsa04932 | Non-alcoholic fatty liver disease | 1/75 | | | 157/8842 | 0.74059972 | | 0.803009945 | 0.640490841 | | FOS | | 1 |  |
| hsa04150 | mTOR signaling pathway | 1/75 | | | 158/8842 | 0.742839791 | | 0.803009945 | 0.640490841 | | IGF1 | | 1 |  |
| hsa05160 | Hepatitis C | 1/75 | | | 159/8842 | 0.745060774 | | 0.803009945 | 0.640490841 | | EGF | | 1 |  |
| hsa05161 | Hepatitis B | 1/75 | | | 163/8842 | 0.75375701 | | 0.807894253 | 0.644386626 | | FOS | | 1 |  |
| hsa04630 | JAK-STAT signaling pathway | 1/75 | | | 168/8842 | 0.764216679 | | 0.814604592 | 0.649738877 | | EGF | | 1 |  |
| hsa05164 | Influenza A | 1/75 | | | 172/8842 | 0.772267726 | | 0.81868819 | 0.652996006 | | CCL2 | | 1 |  |
| hsa04360 | Axon guidance | 1/75 | | | 184/8842 | 0.794828463 | | 0.838025662 | 0.668419817 | | PLCG2 | | 1 |  |
| hsa05034 | Alcoholism | 1/75 | | | 188/8842 | 0.801847021 | | 0.838126223 | 0.668500026 | | FOSB | | 1 |  |
| hsa04621 | NOD-like receptor signaling pathway | 1/75 | | | 189/8842 | 0.803564316 | | 0.838126223 | 0.668500026 | | CCL2 | | 1 |  |
| hsa05130 | Pathogenic Escherichia coli infection | 1/75 | | | 203/8842 | 0.826119395 | | 0.851060112 | 0.678816259 | | FOS | | 1 |  |
| hsa05169 | Epstein-Barr virus infection | 1/75 | | | 203/8842 | 0.826119395 | | 0.851060112 | 0.678816259 | | PLCG2 | | 1 |  |
| hsa05203 | Viral carcinogenesis | 1/75 | | | 205/8842 | 0.829125573 | | 0.851060112 | 0.678816259 | | C3 | | 1 |  |
| hsa05010 | Alzheimer disease | 2/75 | | | 391/8842 | 0.850805586 | | 0.868717282 | 0.69289984 | | PTGS2/LPL | | 2 |  |
| hsa05132 | Salmonella infection | 1/75 | | | 251/8842 | 0.885715967 | | 0.899627736 | 0.717554407 | | FOS | | 1 |  |
| hsa05012 | Parkinson disease | 1/75 | | | 271/8842 | 0.904117463 | | 0.913535354 | 0.728647298 | | DUSP1 | | 1 |  |
| hsa04740 | Olfactory transduction | 1/75 | | | 453/8842 | 0.980964099 | | 0.985466689 | 0.786020636 | | RGS2 | | 1 |  |
| hsa05022 | Pathways of neurodegeneration - multiple diseases | 1/75 | | | 483/8842 | 0.985466689 | | 0.985466689 | 0.786020636 | | PTGS2 | | 1 |  |

### 2.4 Table S4:Candidate gene screening via machine learning.

| DEGs | GEO-brown | intersect | Random Foreast | Lasso | intersect | NETs-gene | intersect |
| --- | --- | --- | --- | --- | --- | --- | --- |
| G6PC | A2M | PDK4 | PDK4 | ADH1B | ADH1B | ALPL | ESM1 |
| FOS | ABCA6 | FN1 | FBLN5 | AEBP1 | AEBP1 | BST1 | VCAN |
| ZFP36 | ABCC9 | G0S2 | FN1 | CCL19 | ESM1 | CD93 |  |
| ALB | ABI3BP | LUM | AEBP1 | CCL2 | FPR3 | CEACAM3 |  |
| S100A12 | ACTA2 | TGFBI | MARCKS | CPA3 | G0S2 | CREB5 |  |
| S100A9 | ACTN1 | COL1A2 | G0S2 | ESM1 | PDK4 | CRISPLD2 |  |
| PDK4 | ADAM10 | ESM1 | CCL18 | FGF9 | RNASE6 | CSF3R |  |
| EGR1 | ADAM12 | MOXD1 | PTN | FPR3 | VCAN | CYP4F3 |  |
| HPD | ADAMTS1 | FGF9 | MOXD1 | G0S2 |  | DYSF |  |
| ESM1 | ADAMTS3 | MMP2 | MMP2 | IGKC |  | FCAR |  |
| PTGS2 | ADH1B | COL6A3 | ESM1 | PDK4 |  | FCGR3B |  |
| TPPP3 | ADRA2A | THBS2 | APOC1 | RNASE6 |  | CPPED1 |  |
| CDH10 | ADRA2B | MARCKS | MFAP4 | TGFBI |  | FPR1 |  |
| LOX | AEBP1 | ADH1B | MS4A6A | THBS2 |  | FPR2 |  |
| GSTA1 | AHR | ASPN | VCAN | UMOD |  | G0S2 |  |
| NPHS1 | AHSA2 | VSIG4 | RNASE6 | VCAN |  | HIST1H2BC |  |
| LPL | ALOX5AP | C7 | IGJ |  |  | HPSE |  |
| UMOD | ANGPT2 | MS4A6A | ADH1B |  |  | CXCR1 |  |
| RGS2 | ANGPTL4 | MFAP4 | HBB |  |  | CXCR2 |  |
| AGMAT | ANKRD10-IT1 | AEBP1 | FPR3 |  |  | KCNJ15 |  |
| EHD3 | ANKRD36 | TNC |  |  |  | LILRB2 |  |
| HAO2 | ANKRD36B | CPA3 |  |  |  | MGAM |  |
| S100A8 | ANO1 | CCL21 |  |  |  | MME |  |
| LRRC2 | ANXA3 | C3 |  |  |  | PDE4B |  |
| SLC12A3 | APOC1 | PTN |  |  |  | S100A12 |  |
| DUSP1 | ARL4C | SERPINF1 |  |  |  | SIGLEC5 |  |
| AFM | ASPN | FBLN5 |  |  |  | SLC22A4 |  |
| JUNB | ATP13A3 | FMO3 |  |  |  | SLC25A37 |  |
| DPP6 | AXL | MMP7 |  |  |  | TECPR2 |  |
| TNNI1 | BASP1 | CDH6 |  |  |  | TNFRSF10C |  |
| HBB | BHLHE41 | CCL19 |  |  |  | VNN3 |  |
| GLYAT | BICC1 | CCL2 |  |  |  | AKT1 |  |
| IGF1 | BIRC3 | FPR3 |  |  |  | AKT2 |  |
| HPGD | BMP5 | VCAN |  |  |  | ATG7 |  |
| CHI3L1 | C10orf10 | IGJ |  |  |  | CLEC6A |  |
| HRSP12 | C1QA | HBB |  |  |  | CSF3 |  |
| PRKAR2B | C1QB | IGKC |  |  |  | CTSG |  |
| CXCR2 | C1R | CCL18 |  |  |  | CYBB |  |
| PTGDS | C1RL | UMOD |  |  |  | DNASE1 |  |
| EGF | C1S | RNASE6 |  |  |  | ELANE |  |
| KNG1 | C2 | APOC1 |  |  |  | ENTPD4 |  |
| ALDOB | C3 |  |  |  |  | F3 |  |
| SST | C3AR1 |  |  |  |  | HMGB1 |  |
| FBP1 | C7 |  |  |  |  | IL17A |  |
| DCXR | CADM1 |  |  |  |  | IL1B |  |
| PCOLCE2 | CADM3 |  |  |  |  | IL6 |  |
| CXCR1 | CAV1 |  |  |  |  | IL8 |  |
| PCK1 | CCL11 |  |  |  |  | IRAK4 |  |
| ARHGAP19 | CCL13 |  |  |  |  | ITGAM |  |
| APOH | CCL18 |  |  |  |  | ITGB2 |  |
| TCF21 | CCL19 |  |  |  |  | KCNN3 |  |
| FABP1 | CCL2 |  |  |  |  | MAPK1 |  |
| DACH1 | CCL20 |  |  |  |  | MAPK3 |  |
| CR1 | CCL21 |  |  |  |  | MMP9 |  |
| NR4A2 | CCL22 |  |  |  |  | MPO |  |
| G0S2 | CCL8 |  |  |  |  | MTOR |  |
| BHMT | CCNA2 |  |  |  |  | PADI4 |  |
| HSD17B14 | CCND2 |  |  |  |  | PTAFR |  |
| B3GALT2 | CCR1 |  |  |  |  | PIK3CA |  |
| TNNT2 | CCR2 |  |  |  |  | RIPK1 |  |
| CA10 | CCR7 |  |  |  |  | RIPK3 |  |
| CYP27B1 | CD14 |  |  |  |  | SELP |  |
| FPR1 | CD163 |  |  |  |  | SELPLG |  |
| HIST1H2BD | CD180 |  |  |  |  | SIGLEC14 |  |
| FOSB | CD1C |  |  |  |  | TLR2 |  |
| PRODH2 | CD2 |  |  |  |  | TLR4 |  |
| MAGI2 | CD248 |  |  |  |  | TLR7 |  |
| APOD | CD37 |  |  |  |  | TLR8 |  |
| FCGR3B | CD3D |  |  |  |  | TNF |  |
| APOM | CD3G |  |  |  |  | SGK1 |  |
| ATF3 | CD44 |  |  |  |  | ACTB |  |
| WT1-AS | CD48 |  |  |  |  | ACTG1 |  |
| CYP2B6 | CD52 |  |  |  |  | ACTN1 |  |
| DDN | CD53 |  |  |  |  | ACTN4 |  |
| CYP4A11 | CD84 |  |  |  |  | ARPIN |  |
| CALB1 | CD86 |  |  |  |  | AZU1 |  |
| PLCG2 | CD93 |  |  |  |  | C3 |  |
| FGF9 | CD96 |  |  |  |  | C3AR1 |  |
| SYNPO | CDA |  |  |  |  | C5AR1 |  |
| TGFBR3 | CDCP1 |  |  |  |  | CAMP |  |
| KLK7 | CDH1 |  |  |  |  | CARD11 |  |
| ALDH6A1 | CDH11 |  |  |  |  | CASP1 |  |
| C1orf21 | CDH2 |  |  |  |  | CAT |  |
| CERS6 | CDH6 |  |  |  |  | CCDC25 |  |
| BTG2 | CEACAM1 |  |  |  |  | CCL2 |  |
| HES1 | CELF2 |  |  |  |  | CCL3 |  |
| GDF15 | CFH |  |  |  |  | CCL4 |  |
| A1CF | CHPT1 |  |  |  |  | CCL5 |  |
| RASL11B | CHST15 |  |  |  |  | CD177 |  |
| USP2 | CKB |  |  |  |  | CD274 |  |
| NFIL3 | CKLF |  |  |  |  | CD44 |  |
| PPBP | CLC |  |  |  |  | CEBPB |  |
| CETP | CLDN1 |  |  |  |  | CFTR |  |
| CCDC91 | CLEC10A |  |  |  |  | CLEC4E |  |
| FXYD1 | CLEC4A |  |  |  |  | CLEC7A |  |
| NAP1L2 | CLEC7A |  |  |  |  | CTSC |  |
| RRAS | CNTN1 |  |  |  |  | CXCL1 |  |
| HTRA1 | COL10A1 |  |  |  |  | CXCL2 |  |
| ANGPTL3 | COL14A1 |  |  |  |  | CXCR4 |  |
| TNNC1 | COL15A1 |  |  |  |  | DEFA3 |  |
| MPP5 | COL1A1 |  |  |  |  | DNAJB1 |  |
| GJA1 | COL1A2 |  |  |  |  | ENO1 |  |
| SBSPON | COL3A1 |  |  |  |  | F2RL2 |  |
| KDR | COL4A1 |  |  |  |  | FCGR2B |  |
| PLTP | COL4A2 |  |  |  |  | FGL2 |  |
| DEFB1 | COL6A3 |  |  |  |  | GPBAR1 |  |
| VNN2 | COL8A1 |  |  |  |  | GSDMD |  |
| ZNF124 | CORO1A |  |  |  |  | H2AX |  |
| GADD45B | CORO1C |  |  |  |  | HIF1A |  |
| EXPH5 | CP |  |  |  |  | HRG |  |
| CA2 | CPA3 |  |  |  |  | IL12A |  |
| SLC5A12 | CPA4 |  |  |  |  | IL1RL1 |  |
| H2AFJ | CPE |  |  |  |  | IL33 |  |
| BST1 | CR2 |  |  |  |  | IL36RN |  |
| XPNPEP2 | CRIP1 |  |  |  |  | IL5 |  |
| JUN | CRISPLD2 |  |  |  |  | ILK |  |
| TSPYL5 | CRYAB |  |  |  |  | IRF1 |  |
| ZNF804A | CSF1R |  |  |  |  | ITGB1 |  |
| USP46 | CTSC |  |  |  |  | KLF2 |  |
| THSD7A | CTSG |  |  |  |  | KRT10 |  |
| TM4SF5 | CTSK |  |  |  |  | LCP1 |  |
| TYRO3 | CTSS |  |  |  |  | LDLR |  |
| FOLH1B | CX3CL1 |  |  |  |  | LPAR3 |  |
| MT1H | CX3CR1 |  |  |  |  | LTF |  |
| PLG | CXCL1 |  |  |  |  | LYZ |  |
| ABAT | CXCL6 |  |  |  |  | MAPK14 |  |
| SLC7A9 | CYBB |  |  |  |  | MAPK7 |  |
| GSTA3 | CYBRD1 |  |  |  |  | MCOLN3 |  |
| SEMA3G | CYP1B1 |  |  |  |  | MFN1 |  |
| EGOT | CYP39A1 |  |  |  |  | MFN2 |  |
| DDC | DACT1 |  |  |  |  | MIR146A |  |
| SLC7A7 | DCBLD2 |  |  |  |  | MIR21 |  |
| DAO | DDX17 |  |  |  |  | MIR223 |  |
| GIPC2 | DKK3 |  |  |  |  | MNDA |  |
| MCM6 | DOCK10 |  |  |  |  | MYD88 |  |
| AKR7A3 | DOCK2 |  |  |  |  | MYH9 |  |
| MYOZ1 | DSP |  |  |  |  | NFE2L2 |  |
| NLK | DUSP6 |  |  |  |  | NFIL3 |  |
| HIST1H1E | EDIL3 |  |  |  |  | NFKBIA |  |
| MAFB | EDNRA |  |  |  |  | NLRP3 |  |
| EPB41L5 | EDNRB |  |  |  |  | NOX4 |  |
| CKB | EFNA5 |  |  |  |  | OPA1 |  |
| SEMA5A | ENAH |  |  |  |  | ORAI1 |  |
| FAM134B | EPB41L2 |  |  |  |  | P2RX1 |  |
| SOWAHC | EPHA3 |  |  |  |  | PARVB |  |
| ECHS1 | ESM1 |  |  |  |  | PF4 |  |
| PI3 | EVI2A |  |  |  |  | PKM |  |
| SLC7A8 | EXT1 |  |  |  |  | PROCR |  |
| SORD | FABP5 |  |  |  |  | PRTN3 |  |
| ERVMER34-1 | FAM129A |  |  |  |  | S100A8 |  |
| GBA3 | FBLN5 |  |  |  |  | S100A9 |  |
| ALDH4A1 | FBN1 |  |  |  |  | S1PR2 |  |
| ASPA | FCER1G |  |  |  |  | SOCS3 |  |
| KLK1 | FCGR1A |  |  |  |  | SPP1 |  |
| SLC13A3 | FCGR1B |  |  |  |  | SRC |  |
| MYL9 | FCGR2A |  |  |  |  | STAT3 |  |
| PBLD | FCGR2B |  |  |  |  | SUCNR1 |  |
| PEPD | FCGR2C |  |  |  |  | SYK |  |
| FRY | FGA |  |  |  |  | TICAM1 |  |
| FOXC1 | FGF7 |  |  |  |  | TIMP1 |  |
| CLEC4E | FGF9 |  |  |  |  | TKT |  |
| PLCE1 | FGL2 |  |  |  |  | TLR9 |  |
| HIST2H2BE | FHL2 |  |  |  |  | TNFAIP3 |  |
| CDKN1B | FILIP1L |  |  |  |  | WASL |  |
| AQP9 | FLT3 |  |  |  |  | XIST |  |
| NDNF | FMO3 |  |  |  |  | PRKCD |  |
| NFASC | FMOD |  |  |  |  | DEFA1 |  |
| PVALB | FN1 |  |  |  |  | JAK2 |  |
| HSD17B2 | FOXF1 |  |  |  |  | FRAS1 |  |
| F3 | FPR3 |  |  |  |  | CASP3 |  |
| TREM1 | FRZB |  |  |  |  | MMP3 |  |
| MYLIP | FXYD5 |  |  |  |  | HSPG2 |  |
| SIK1 | FYB |  |  |  |  | CYBA |  |
| CX3CL1 | G0S2 |  |  |  |  | CRP |  |
| IL1RL1 | GALNT1 |  |  |  |  | EGFR |  |
| TENC1 | GATA6 |  |  |  |  | SELE |  |
| ZDHHC6 | GBP1 |  |  |  |  | VTN |  |
| SPTB | GBP2 |  |  |  |  | IL10 |  |
| FMO1 | GFRA1 |  |  |  |  | CD36 |  |
| ADI1 | GLIPR1 |  |  |  |  | CXCL5 |  |
| PSAT1 | GOLGA8A |  |  |  |  | VEGFA |  |
| PIPOX | GPC4 |  |  |  |  | PPBP |  |
| ECH1 | GPHN |  |  |  |  | FBN1 |  |
| ENPEP | GPR126 |  |  |  |  | APP |  |
| MAOA | GPR171 |  |  |  |  | MMP1 |  |
| WT1 | GPR18 |  |  |  |  | SERPINB1 |  |
| PTPRO | GPR183 |  |  |  |  | IFNG |  |
| KLK6 | GPR27 |  |  |  |  | MEPE |  |
| FBXO21 | GPR65 |  |  |  |  | H19 |  |
| BHMT2 | GUCY1A3 |  |  |  |  | SMARCD2 |  |
| AIF1 | GZMA |  |  |  |  | IL1RN |  |
| FAM65A | GZMK |  |  |  |  | FN1 |  |
| FZD2 | HBB |  |  |  |  | IL1A |  |
| NR4A1 | HBD |  |  |  |  | SELL |  |
| PAMR1 | HCLS1 |  |  |  |  | CSF2 |  |
| KIRREL | HGF |  |  |  |  | ICAM1 |  |
| CTH | HIF1A |  |  |  |  | TGFB1 |  |
| RGN | HIST1H1D |  |  |  |  | ELN |  |
| HGD | HIST1H2AM |  |  |  |  | MMP2 |  |
| PTH1R | HIST1H3B |  |  |  |  | SOD1 |  |
| TCEAL2 | HIST1H3F |  |  |  |  | PIK3CG |  |
| ITGA8 | HIST1H4E |  |  |  |  | MMP8 |  |
| XYLT1 | HIST2H2BE |  |  |  |  | EFEMP2 |  |
| CRYAA | HLA-DPA1 |  |  |  |  | SOD3 |  |
| FBXO17 | HLA-DQB1 |  |  |  |  | MEFV |  |
| SALL1 | HLA-DRA |  |  |  |  | ECM1 |  |
| DIO1 | HOXD10 |  |  |  |  | EFEMP1 |  |
| APOLD1 | HRH1 |  |  |  |  | LCN2 |  |
| SLC27A2 | HSD17B2 |  |  |  |  | NCF4 |  |
| SLC2A2 | HSPG2 |  |  |  |  | NCF1 |  |
| ANXA9 | HTR2B |  |  |  |  | NCF2 |  |
| MXRA8 | IFI16 |  |  |  |  | CXCL8 |  |
| FMO4 | IFI44 |  |  |  |  | RAC2 |  |
| BMP7 | IFIT2 |  |  |  |  | TNFRSF1A |  |
| HRG | IGFBP3 |  |  |  |  | ALPL |  |
| CLIP3 | IGFBP6 |  |  |  |  | BST1 |  |
| MME | IGHM |  |  |  |  | CD93 |  |
| DPEP1 | IGJ |  |  |  |  | CEACAM3 |  |
| OLFML2A | IGKC |  |  |  |  | CREB5 |  |
| KLF7 | IGLV6-57 |  |  |  |  | CRISPLD2 |  |
| MT1G | IGSF6 |  |  |  |  | CSF3R |  |
| KBTBD11 | IL10RA |  |  |  |  | CYP4F3 |  |
| ETFB | IL2RG |  |  |  |  | DYSF |  |
| BLVRB | IL33 |  |  |  |  | FCAR |  |
| MACROD1 | IL7R |  |  |  |  | FCGR3B |  |
| NAMPT | INHBA |  |  |  |  | CPPED1 |  |
| SPOCK2 | IRF8 |  |  |  |  | FPR1 |  |
| CDKN1C | ITGA2 |  |  |  |  | FPR2 |  |
| HIST1H1C | ITGA4 |  |  |  |  | G0S2 |  |
| EPHX2 | ITGBL1 |  |  |  |  | HIST1H2BC |  |
| FGF1 | ITIH2 |  |  |  |  | HPSE |  |
| PCBD1 | ITM2C |  |  |  |  | CXCR1 |  |
| HSPA1L | JAG1 |  |  |  |  | CXCR2 |  |
| TGFBR2 | KCTD12 |  |  |  |  | KCNJ15 |  |
| RARRES2 | KITLG |  |  |  |  | LILRB2 |  |
| ELOVL4 | KPNA2 |  |  |  |  | MGAM |  |
| PLAT | KRT23 |  |  |  |  | MME |  |
| CYB5A | LAMA2 |  |  |  |  | PDE4B |  |
| CEL | LAMA4 |  |  |  |  | S100A12 |  |
| RHCG | LAMC1 |  |  |  |  | SIGLEC5 |  |
| CRYL1 | LAMC2 |  |  |  |  | SLC22A4 |  |
| PPAP2B | LAMP5 |  |  |  |  | SLC25A37 |  |
| HSD11B2 | LAPTM5 |  |  |  |  | TECPR2 |  |
| CITED2 | LCP1 |  |  |  |  | TNFRSF10C |  |
| KLF9 | LGALS9 |  |  |  |  | VNN3 |  |
| FLRT3 | LGI2 |  |  |  |  | AKT1 |  |
| RBP4 | LINC00342 |  |  |  |  | AKT2 |  |
| HS3ST3A1 | LINC00597 |  |  |  |  | ATG7 |  |
| CORO2B | LOC100272216 |  |  |  |  | CLEC6A |  |
| CNTN1 | LOXL1 |  |  |  |  | CSF3 |  |
| BCAM | LPAR1 |  |  |  |  | CTSG |  |
| FOXD1 | LRP1 |  |  |  |  | CYBB |  |
| RAMP3 | LTB |  |  |  |  | DNASE1 |  |
| KHK | LTBP1 |  |  |  |  | ELANE |  |
| ACADSB | LTBP2 |  |  |  |  | ENTPD4 |  |
| ALPL | LTF |  |  |  |  | F3 |  |
| PAK1 | LUM |  |  |  |  | HMGB1 |  |
| GADD45G | LXN |  |  |  |  | IL17A |  |
| FABP3 | LY86 |  |  |  |  | IL1B |  |
| MNDA | LY96 |  |  |  |  | IL6 |  |
| ZNF185 | LYPD1 |  |  |  |  | IL8 |  |
| MRPL16 | LYVE1 |  |  |  |  | IRAK4 |  |
| EHHADH | LYZ |  |  |  |  | ITGAM |  |
| CYP4F3 | MARCKS |  |  |  |  | ITGB2 |  |
| B3GNT1 | MCAM |  |  |  |  | KCNN3 |  |
| MT1F | MECOM |  |  |  |  | MAPK1 |  |
| C11orf63 | MEST |  |  |  |  | MAPK3 |  |
| HRASLS2 | MFAP4 |  |  |  |  | MMP9 |  |
| GMDS | MFHAS1 |  |  |  |  | MPO |  |
| TYRP1 | MGP |  |  |  |  | MTOR |  |
| GAS6 | MICAL2 |  |  |  |  | PADI4 |  |
| ARG2 | MIR3916 |  |  |  |  | PTAFR |  |
| ECM2 | MMP14 |  |  |  |  | PIK3CA |  |
| PVRL2 | MMP2 |  |  |  |  | RIPK1 |  |
| PCMTD2 | MMP7 |  |  |  |  | RIPK3 |  |
| ID4 | MOXD1 |  |  |  |  | SELP |  |
| FAM20B | MPZL2 |  |  |  |  | SELPLG |  |
| ZNF204P | MS4A1 |  |  |  |  | SIGLEC14 |  |
| GTF2A2 | MS4A4A |  |  |  |  | TLR2 |  |
| DPYS | MS4A6A |  |  |  |  | TLR4 |  |
| ACSM5 | MSC |  |  |  |  | TLR7 |  |
| SIRPA | MSR1 |  |  |  |  | TLR8 |  |
| ALDH2 | MXRA5 |  |  |  |  | TNF |  |
| TST | MYC |  |  |  |  | SGK1 |  |
| NQO1 | MYH10 |  |  |  |  | ACTB |  |
| MTHFD2 | MYH11 |  |  |  |  | ACTG1 |  |
| CLDN8 | MYL3 |  |  |  |  | ACTN1 |  |
| METTL21B | MYOF |  |  |  |  | ACTN4 |  |
| KCNJ15 | N4BP2L2-IT2 |  |  |  |  | ARPIN |  |
| SLC26A4 | NABP1 |  |  |  |  | AZU1 |  |
| TSPAN2 | NAIP |  |  |  |  | C3 |  |
| AZGP1 | NCKAP1L |  |  |  |  | C3AR1 |  |
| ID1 | NEFL |  |  |  |  | C5AR1 |  |
| MYCT1 | NFIX |  |  |  |  | CAMP |  |
| ACOX2 | NID1 |  |  |  |  | CARD11 |  |
| SLC9A3R1 | NID2 |  |  |  |  | CASP1 |  |
| NDN | NKTR |  |  |  |  | CAT |  |
| ITIH5 | NMU |  |  |  |  | CCDC25 |  |
| SERINC5 | NPY1R |  |  |  |  | CCL2 |  |
| CAND2 | NT5E |  |  |  |  | CCL3 |  |
| CDH13 | NUPR1 |  |  |  |  | CCL4 |  |
| C11orf71 | NUSAP1 |  |  |  |  | CCL5 |  |
| NR4A3 | OCLM |  |  |  |  | CD177 |  |
| SPRY4 | OGN |  |  |  |  | CD274 |  |
| GK | OLFM4 |  |  |  |  | CD44 |  |
| NRIP2 | OLFML2B |  |  |  |  | CEBPB |  |
| BTBD3 | OSMR |  |  |  |  | CFTR |  |
| FAM98A | P2RY13 |  |  |  |  | CLEC4E |  |
| CTNNBIP1 | P2RY14 |  |  |  |  | CLEC7A |  |
| F5 | PAK1 |  |  |  |  | CTSC |  |
| VEGFA | PALMD |  |  |  |  | CXCL1 |  |
| RNF220 | PAPPA |  |  |  |  | CXCL2 |  |
| HEBP1 | PAPPA2 |  |  |  |  | CXCR4 |  |
| ASMTL | PCDHGA10 |  |  |  |  | DEFA3 |  |
| EMCN | PDCD1LG2 |  |  |  |  | DNAJB1 |  |
| AKR7A2 | PDE1A |  |  |  |  | ENO1 |  |
| APOE | PDE3A |  |  |  |  | F2RL2 |  |
| TACC2 | PDGFRA |  |  |  |  | FCGR2B |  |
| DHRS3 | PDK4 |  |  |  |  | FGL2 |  |
| MGAM | PDLIM1 |  |  |  |  | GPBAR1 |  |
| AHCY | PELI2 |  |  |  |  | GSDMD |  |
| SELL | PKD2 |  |  |  |  | H2AX |  |
| NTNG1 | PLAU |  |  |  |  | HIF1A |  |
| CYP39A1 | PLAUR |  |  |  |  | HRG |  |
| FAM174B | PLEK |  |  |  |  | IL12A |  |
| HPS5 | PLK2 |  |  |  |  | IL1RL1 |  |
| NPL | PLN |  |  |  |  | IL33 |  |
| FRAT1 | PLVAP |  |  |  |  | IL36RN |  |
| SUCO | PLXDC2 |  |  |  |  | IL5 |  |
| GHR | PMP22 |  |  |  |  | ILK |  |
| PDZK1IP1 | POSTN |  |  |  |  | IRF1 |  |
| PDZK1 | PPBP |  |  |  |  | ITGB1 |  |
| FPR2 | PPP1R3C |  |  |  |  | KLF2 |  |
| PLA2R1 | PRDM1 |  |  |  |  | KRT10 |  |
| PLBD1 | PRELP |  |  |  |  | LCP1 |  |
| USH1C | PROM1 |  |  |  |  | LDLR |  |
| PNMA1 | PROS1 |  |  |  |  | LPAR3 |  |
| GAS1 | PRSS23 |  |  |  |  | LTF |  |
| ITGB5 | PTH1R |  |  |  |  | LYZ |  |
| CYP26B1 | PTN |  |  |  |  | MAPK14 |  |
| TMEM204 | PTPRC |  |  |  |  | MAPK7 |  |
| C1orf115 | PXDN |  |  |  |  | MCOLN3 |  |
| TIPARP | QPCT |  |  |  |  | MFN1 |  |
| ASS1 | RAB32 |  |  |  |  | MFN2 |  |
| CHPT1 | RAB3B |  |  |  |  | MIR146A |  |
| MGAT5 | RARRES1 |  |  |  |  | MIR21 |  |
| ABCC2 | RARRES2 |  |  |  |  | MIR223 |  |
| GALC | RASSF2 |  |  |  |  | MNDA |  |
| ANK2 | RASSF9 |  |  |  |  | MYD88 |  |
| CRABP1 | RELN |  |  |  |  | MYH9 |  |
| GPR27 | RERGL |  |  |  |  | NFE2L2 |  |
| SCGB1D2 | RGCC |  |  |  |  | NFIL3 |  |
| MXD1 | RGS4 |  |  |  |  | NFKBIA |  |
| SUCLG1 | RGS5 |  |  |  |  | NLRP3 |  |
| CDC42EP3 | RNASE6 |  |  |  |  | NOX4 |  |
| VPS28 | RND1 |  |  |  |  | OPA1 |  |
| PDLIM2 | RPL23AP32 |  |  |  |  | ORAI1 |  |
| ALS2CL | S100A11 |  |  |  |  | P2RX1 |  |
| MMP28 | SACS |  |  |  |  | PARVB |  |
| MBNL3 | SAMHD1 |  |  |  |  | PF4 |  |
| UQCR10 | SAMSN1 |  |  |  |  | PKM |  |
| C5AR1 | SCD |  |  |  |  | PROCR |  |
| GTF2H5 | SCG5 |  |  |  |  | PRTN3 |  |
| RRAGD | SEMA3C |  |  |  |  | S100A8 |  |
| SLC6A13 | SERPINA3 |  |  |  |  | S100A9 |  |
| FUT6 | SERPINE1 |  |  |  |  | S1PR2 |  |
| NQO2 | SERPINF1 |  |  |  |  | SOCS3 |  |
| RABAC1 | SETBP1 |  |  |  |  | SPP1 |  |
| APOO | SFRP4 |  |  |  |  | SRC |  |
| ETNK2 | SLAMF7 |  |  |  |  | STAT3 |  |
| CLIC5 | SLC12A2 |  |  |  |  | SUCNR1 |  |
| ACO2 | SLC16A10 |  |  |  |  | SYK |  |
| GOLIM4 | SLC22A3 |  |  |  |  | TICAM1 |  |
| ACOT13 | SLC34A2 |  |  |  |  | TIMP1 |  |
| MYO5C | SLC38A1 |  |  |  |  | TKT |  |
| SNTA1 | SLC38A2 |  |  |  |  | TLR9 |  |
| ARNTL | SLC4A7 |  |  |  |  | TNFAIP3 |  |
| HBD | SLC6A6 |  |  |  |  | WASL |  |
| PRDM1 | SLC7A2 |  |  |  |  | XIST |  |
| KL | SLIT3 |  |  |  |  | PRKCD |  |
| IQGAP2 | SLPI |  |  |  |  | DEFA1 |  |
| ARHGEF26 | SMA4 |  |  |  |  | JAK2 |  |
| PRDX6 | SOX4 |  |  |  |  | FRAS1 |  |
| C11orf1 | SPDYE2 |  |  |  |  | CASP3 |  |
| CCBL1 | SPON1 |  |  |  |  | MMP3 |  |
| EMC6 | SRGN |  |  |  |  | HSPG2 |  |
| WISP3 | SRPX |  |  |  |  | CYBA |  |
| PCYOX1 | STX11 |  |  |  |  | CRP |  |
| QDPR | SVEP1 |  |  |  |  | EGFR |  |
| MTTP | SYT11 |  |  |  |  | SELE |  |
| BPHL | SYTL2 |  |  |  |  | VTN |  |
| PHYHIP | TAC1 |  |  |  |  | IL10 |  |
| BAIAP2 | TAGLN |  |  |  |  | CD36 |  |
| H1FX | TES |  |  |  |  | CXCL5 |  |
| SULT1A1 | TFPI2 |  |  |  |  | VEGFA |  |
| GPX3 | TGFB2 |  |  |  |  | PPBP |  |
| ADH6 | TGFBI |  |  |  |  | FBN1 |  |
| FAH | THBD |  |  |  |  | APP |  |
| ATP5D | THBS2 |  |  |  |  | MMP1 |  |
| TCEB2 | TIMP1 |  |  |  |  | SERPINB1 |  |
| MAPT | TLR1 |  |  |  |  | IFNG |  |
| CDA | TLR2 |  |  |  |  | MEPE |  |
| NPHS2 | TLR7 |  |  |  |  | H19 |  |
| MAN2A2 | TM6SF1 |  |  |  |  | SMARCD2 |  |
| ZBTB16 | TNC |  |  |  |  | IL1RN |  |
| CTDSPL | TNFAIP3 |  |  |  |  | FN1 |  |
| PC | TNFAIP6 |  |  |  |  | IL1A |  |
| TJP1 | TNFRSF11B |  |  |  |  | SELL |  |
| SLC6A2 | TNFRSF12A |  |  |  |  | CSF2 |  |
| ARHGAP28 | TNFRSF17 |  |  |  |  | ICAM1 |  |
| FAXDC2 | TNIK |  |  |  |  | TGFB1 |  |
| HSPB8 | TOP2A |  |  |  |  | ELN |  |
| ZBTB10 | TPM1 |  |  |  |  | MMP2 |  |
| WDR83OS | TPSAB1 |  |  |  |  | SOD1 |  |
| LPPR1 | TSHZ2 |  |  |  |  | PIK3CG |  |
| HIST1H2AC | TSPAN13 |  |  |  |  | MMP8 |  |
| MAN2B2 | TTR |  |  |  |  | EFEMP2 |  |
| FAM127B | TYROBP |  |  |  |  | SOD3 |  |
| PPDPF | TYRP1 |  |  |  |  | MEFV |  |
| SULT1A2 | UCP2 |  |  |  |  | ECM1 |  |
| LRRC6 | UGCG |  |  |  |  | EFEMP1 |  |
| OCEL1 | UMOD |  |  |  |  | LCN2 |  |
| ADRBK2 | VCAM1 |  |  |  |  | NCF4 |  |
| PODXL | VCAN |  |  |  |  | NCF1 |  |
| PTPRD | VEGFC |  |  |  |  | NCF2 |  |
| DLG2 | VLDLR |  |  |  |  | CXCL8 |  |
| ZNF16 | VSIG4 |  |  |  |  | RAC2 |  |
| PNMA2 | WSB1 |  |  |  |  | TNFRSF1A |  |
| TSPYL4 | ZBED2 |  |  |  |  |  |  |
| TRAM2 | ZNF204P |  |  |  |  |  |  |
| COX8A | ZNF91 |  |  |  |  |  |  |
| MUT |  |  |  |  |  |  |  |
| PRLR |  |  |  |  |  |  |  |
| NDUFC1 |  |  |  |  |  |  |  |
| DHTKD1 |  |  |  |  |  |  |  |
| CRYAB |  |  |  |  |  |  |  |
| LAMA2 |  |  |  |  |  |  |  |
| DYNC1H1 |  |  |  |  |  |  |  |
| SETBP1 |  |  |  |  |  |  |  |
| ANKRD10 |  |  |  |  |  |  |  |
| RAB3B |  |  |  |  |  |  |  |
| OGT |  |  |  |  |  |  |  |
| TNFRSF11B |  |  |  |  |  |  |  |
| LGALS9 |  |  |  |  |  |  |  |
| CBFB |  |  |  |  |  |  |  |
| LAMC1 |  |  |  |  |  |  |  |
| EXT1 |  |  |  |  |  |  |  |
| GALNT1 |  |  |  |  |  |  |  |
| KCNJ8 |  |  |  |  |  |  |  |
| MYH10 |  |  |  |  |  |  |  |
| CLK1 |  |  |  |  |  |  |  |
| GGT5 |  |  |  |  |  |  |  |
| SACS |  |  |  |  |  |  |  |
| TUBB2B |  |  |  |  |  |  |  |
| DOCK10 |  |  |  |  |  |  |  |
| DKK3 |  |  |  |  |  |  |  |
| DDX17 |  |  |  |  |  |  |  |
| SEL1L3 |  |  |  |  |  |  |  |
| GPR52 |  |  |  |  |  |  |  |
| KPNA2 |  |  |  |  |  |  |  |
| BLNK |  |  |  |  |  |  |  |
| MSR1 |  |  |  |  |  |  |  |
| SPATS2 |  |  |  |  |  |  |  |
| TRIM25 |  |  |  |  |  |  |  |
| NKTR |  |  |  |  |  |  |  |
| MALL |  |  |  |  |  |  |  |
| MAP4K4 |  |  |  |  |  |  |  |
| NEFL |  |  |  |  |  |  |  |
| FRZB |  |  |  |  |  |  |  |
| CCNA2 |  |  |  |  |  |  |  |
| IFI44 |  |  |  |  |  |  |  |
| CD2 |  |  |  |  |  |  |  |
| ISLR |  |  |  |  |  |  |  |
| TCF4 |  |  |  |  |  |  |  |
| MMP14 |  |  |  |  |  |  |  |
| PDLIM1 |  |  |  |  |  |  |  |
| MECOM |  |  |  |  |  |  |  |
| HDAC9 |  |  |  |  |  |  |  |
| EVI2A |  |  |  |  |  |  |  |
| CFB |  |  |  |  |  |  |  |
| CLEC10A |  |  |  |  |  |  |  |
| BHLHE41 |  |  |  |  |  |  |  |
| ADAM15 |  |  |  |  |  |  |  |
| CDH2 |  |  |  |  |  |  |  |
| COL1A1 |  |  |  |  |  |  |  |
| PXDN |  |  |  |  |  |  |  |
| NID2 |  |  |  |  |  |  |  |
| DOCK9 |  |  |  |  |  |  |  |
| DSP |  |  |  |  |  |  |  |
| LY86 |  |  |  |  |  |  |  |
| TNFRSF21 |  |  |  |  |  |  |  |
| OSMR |  |  |  |  |  |  |  |
| UCHL1 |  |  |  |  |  |  |  |
| CSF1R |  |  |  |  |  |  |  |
| MXRA5 |  |  |  |  |  |  |  |
| COL10A1 |  |  |  |  |  |  |  |
| ABLIM1 |  |  |  |  |  |  |  |
| ADAM10 |  |  |  |  |  |  |  |
| CPE |  |  |  |  |  |  |  |
| COL4A1 |  |  |  |  |  |  |  |
| IL18 |  |  |  |  |  |  |  |
| BICC1 |  |  |  |  |  |  |  |
| LY96 |  |  |  |  |  |  |  |
| TOP2A |  |  |  |  |  |  |  |
| GPR56 |  |  |  |  |  |  |  |
| KCTD12 |  |  |  |  |  |  |  |
| RAPGEF5 |  |  |  |  |  |  |  |
| EPB41L2 |  |  |  |  |  |  |  |
| PDGFRA |  |  |  |  |  |  |  |
| S100A2 |  |  |  |  |  |  |  |
| BMP5 |  |  |  |  |  |  |  |
| LTB |  |  |  |  |  |  |  |
| FCER1G |  |  |  |  |  |  |  |
| MCAM |  |  |  |  |  |  |  |
| LINC00597 |  |  |  |  |  |  |  |
| FABP5 |  |  |  |  |  |  |  |
| IL10RA |  |  |  |  |  |  |  |
| HIF1A |  |  |  |  |  |  |  |
| GLIPR1 |  |  |  |  |  |  |  |
| LAPTM5 |  |  |  |  |  |  |  |
| FGF7 |  |  |  |  |  |  |  |
| NCKAP1L |  |  |  |  |  |  |  |
| C3AR1 |  |  |  |  |  |  |  |
| LAMA4 |  |  |  |  |  |  |  |
| LIMA1 |  |  |  |  |  |  |  |
| DHCR24 |  |  |  |  |  |  |  |
| PCDH17 |  |  |  |  |  |  |  |
| CPVL |  |  |  |  |  |  |  |
| CP |  |  |  |  |  |  |  |
| PRSS23 |  |  |  |  |  |  |  |
| WSB1 |  |  |  |  |  |  |  |
| ANXA1 |  |  |  |  |  |  |  |
| PMP22 |  |  |  |  |  |  |  |
| FOLR2 |  |  |  |  |  |  |  |
| TM6SF1 |  |  |  |  |  |  |  |
| HGF |  |  |  |  |  |  |  |
| ADH1C |  |  |  |  |  |  |  |
| TSHZ2 |  |  |  |  |  |  |  |
| SPARCL1 |  |  |  |  |  |  |  |
| SERPINE2 |  |  |  |  |  |  |  |
| IL33 |  |  |  |  |  |  |  |
| NMU |  |  |  |  |  |  |  |
| CASP3 |  |  |  |  |  |  |  |
| CD3D |  |  |  |  |  |  |  |
| CD44 |  |  |  |  |  |  |  |
| IGFBP3 |  |  |  |  |  |  |  |
| LPAR1 |  |  |  |  |  |  |  |
| GPR65 |  |  |  |  |  |  |  |
| N4BP2L2-IT2 |  |  |  |  |  |  |  |
| ANKRD36B |  |  |  |  |  |  |  |
| HAVCR1 |  |  |  |  |  |  |  |
| CRIP1 |  |  |  |  |  |  |  |
| LPAR6 |  |  |  |  |  |  |  |
| CORO1C |  |  |  |  |  |  |  |
| ADAMTSL3 |  |  |  |  |  |  |  |
| RGS5 |  |  |  |  |  |  |  |
| PPP1R3C |  |  |  |  |  |  |  |
| ARGLU1 |  |  |  |  |  |  |  |
| LRP2BP |  |  |  |  |  |  |  |
| HPGDS |  |  |  |  |  |  |  |
| S100A4 |  |  |  |  |  |  |  |
| DACT1 |  |  |  |  |  |  |  |
| JAG1 |  |  |  |  |  |  |  |
| VEGFC |  |  |  |  |  |  |  |
| MEST |  |  |  |  |  |  |  |
| P2RY14 |  |  |  |  |  |  |  |
| CLDN1 |  |  |  |  |  |  |  |
| IGLV6-57 |  |  |  |  |  |  |  |
| CD48 |  |  |  |  |  |  |  |
| HTR2B |  |  |  |  |  |  |  |
| CYBB |  |  |  |  |  |  |  |
| FILIP1L |  |  |  |  |  |  |  |
| PRELP |  |  |  |  |  |  |  |
| ANKRD10-IT1 |  |  |  |  |  |  |  |
| C1QA |  |  |  |  |  |  |  |
| RGS4 |  |  |  |  |  |  |  |
| ANGPT2 |  |  |  |  |  |  |  |
| PLN |  |  |  |  |  |  |  |
| CFH |  |  |  |  |  |  |  |
| SLC12A2 |  |  |  |  |  |  |  |
| LOC100272216 |  |  |  |  |  |  |  |
| IRF8 |  |  |  |  |  |  |  |
| GFRA1 |  |  |  |  |  |  |  |
| IL7R |  |  |  |  |  |  |  |
| RASSF9 |  |  |  |  |  |  |  |
| ACTA2 |  |  |  |  |  |  |  |
| GPR183 |  |  |  |  |  |  |  |
| PER3 |  |  |  |  |  |  |  |
| C1QB |  |  |  |  |  |  |  |
| GOLGA8A |  |  |  |  |  |  |  |
| CCND2 |  |  |  |  |  |  |  |
| GZMK |  |  |  |  |  |  |  |
| TGFB2 |  |  |  |  |  |  |  |
| CD52 |  |  |  |  |  |  |  |
| F2RL2 |  |  |  |  |  |  |  |
| CTSK |  |  |  |  |  |  |  |
| IL32 |  |  |  |  |  |  |  |
| SYTL2 |  |  |  |  |  |  |  |
| TLR7 |  |  |  |  |  |  |  |
| PDE1A |  |  |  |  |  |  |  |
| SLC38A1 |  |  |  |  |  |  |  |
| SFRP1 |  |  |  |  |  |  |  |
| CXCL6 |  |  |  |  |  |  |  |
| WFDC2 |  |  |  |  |  |  |  |
| CD93 |  |  |  |  |  |  |  |
| ADAMTS5 |  |  |  |  |  |  |  |
| ACTN1 |  |  |  |  |  |  |  |
| SERPINA3 |  |  |  |  |  |  |  |
| OLFML2B |  |  |  |  |  |  |  |
| CD24 |  |  |  |  |  |  |  |
| PLVAP |  |  |  |  |  |  |  |
| TES |  |  |  |  |  |  |  |
| TPM1 |  |  |  |  |  |  |  |
| FMOD |  |  |  |  |  |  |  |
| TNFRSF12A |  |  |  |  |  |  |  |
| CCL11 |  |  |  |  |  |  |  |
| SAMHD1 |  |  |  |  |  |  |  |
| RPL23AP32 |  |  |  |  |  |  |  |
| SVEP1 |  |  |  |  |  |  |  |
| LTF |  |  |  |  |  |  |  |
| ENAH |  |  |  |  |  |  |  |
| COL14A1 |  |  |  |  |  |  |  |
| EDNRB |  |  |  |  |  |  |  |
| SYT11 |  |  |  |  |  |  |  |
| PCDHGA10 |  |  |  |  |  |  |  |
| SMA4 |  |  |  |  |  |  |  |
| SLC6A6 |  |  |  |  |  |  |  |
| ANKRD36 |  |  |  |  |  |  |  |
| LINC00342 |  |  |  |  |  |  |  |
| SOX4 |  |  |  |  |  |  |  |
| ABCC9 |  |  |  |  |  |  |  |
| CD248 |  |  |  |  |  |  |  |
| NAIP |  |  |  |  |  |  |  |
| VCAM1 |  |  |  |  |  |  |  |
| CTSC |  |  |  |  |  |  |  |
| CDH11 |  |  |  |  |  |  |  |
| ABCA8 |  |  |  |  |  |  |  |
| CD163 |  |  |  |  |  |  |  |
| POSTN |  |  |  |  |  |  |  |
| CD1C |  |  |  |  |  |  |  |
| CD53 |  |  |  |  |  |  |  |
| NT5E |  |  |  |  |  |  |  |
| SFRP4 |  |  |  |  |  |  |  |
| ADAMTS1 |  |  |  |  |  |  |  |
| TAGLN |  |  |  |  |  |  |  |
| ARL4C |  |  |  |  |  |  |  |
| FCER1A |  |  |  |  |  |  |  |
| RARRES1 |  |  |  |  |  |  |  |
| ABI3BP |  |  |  |  |  |  |  |
| OCLM |  |  |  |  |  |  |  |
| SLIT3 |  |  |  |  |  |  |  |
| MIR3916 |  |  |  |  |  |  |  |
| COL3A1 |  |  |  |  |  |  |  |
| ECM1 |  |  |  |  |  |  |  |
| CLU |  |  |  |  |  |  |  |
| ATP13A3 |  |  |  |  |  |  |  |
| FBN1 |  |  |  |  |  |  |  |
| SPDYE2 |  |  |  |  |  |  |  |
| LTBP1 |  |  |  |  |  |  |  |
| GPR18 |  |  |  |  |  |  |  |
| LYVE1 |  |  |  |  |  |  |  |
| GUCY1A3 |  |  |  |  |  |  |  |
| EMP1 |  |  |  |  |  |  |  |
| COL15A1 |  |  |  |  |  |  |  |
| IGFBP6 |  |  |  |  |  |  |  |
| COLEC12 |  |  |  |  |  |  |  |
| PROM1 |  |  |  |  |  |  |  |
| INHBA |  |  |  |  |  |  |  |
| COL8A1 |  |  |  |  |  |  |  |
| PLK2 |  |  |  |  |  |  |  |
| MS4A4A |  |  |  |  |  |  |  |
| MRC1 |  |  |  |  |  |  |  |
| KRT19 |  |  |  |  |  |  |  |
| AEBP1 |  |  |  |  |  |  |  |
| MFAP4 |  |  |  |  |  |  |  |
| IGKC |  |  |  |  |  |  |  |
| FMO3 |  |  |  |  |  |  |  |
| MMP2 |  |  |  |  |  |  |  |
| TNC |  |  |  |  |  |  |  |
| TGFBI |  |  |  |  |  |  |  |
| PTN |  |  |  |  |  |  |  |
| RNASE6 |  |  |  |  |  |  |  |
| FPR3 |  |  |  |  |  |  |  |
| THBS2 |  |  |  |  |  |  |  |
| SERPINF1 |  |  |  |  |  |  |  |
| ASPN |  |  |  |  |  |  |  |
| CPA3 |  |  |  |  |  |  |  |
| APOC1 |  |  |  |  |  |  |  |
| VSIG4 |  |  |  |  |  |  |  |
| FBLN5 |  |  |  |  |  |  |  |
| CDH6 |  |  |  |  |  |  |  |
| CCL2 |  |  |  |  |  |  |  |
| CCL18 |  |  |  |  |  |  |  |
| MARCKS |  |  |  |  |  |  |  |
| MS4A6A |  |  |  |  |  |  |  |
| COL6A3 |  |  |  |  |  |  |  |
| COL1A2 |  |  |  |  |  |  |  |
| ADH1B |  |  |  |  |  |  |  |
| CCL19 |  |  |  |  |  |  |  |
| VCAN |  |  |  |  |  |  |  |
| IGJ |  |  |  |  |  |  |  |
| MOXD1 |  |  |  |  |  |  |  |
| CCL21 |  |  |  |  |  |  |  |
| C7 |  |  |  |  |  |  |  |
| C3 |  |  |  |  |  |  |  |
| MMP7 |  |  |  |  |  |  |  |
| LUM |  |  |  |  |  |  |  |
| FN1 |  |  |  |  |  |  |  |

| **2.5 Table S5: List of DKD-contextualized NET-related genes (DKD-NRGs).** | | |
| --- | --- | --- |
| No. | Gene Symbol | Full Gene Name |
| 1 | CREB5 | cAMP responsive element binding protein 5 |
| 2 | CXCR2 | C-X-C motif chemokine receptor 2 |
| 3 | MME | Membrane metallo-endopeptidase |
| 4 | S100A12 | S100 calcium binding protein A12 |
| 5 | AKT1 | AKT serine/threonine kinase 1 |
| 6 | AKT2 | AKT serine/threonine kinase 2 |
| 7 | ATG7 | Autophagy related 7 |
| 8 | CYBB | Cytochrome b-245 beta chain |
| 9 | ENTPD4 | Ectonucleoside triphosphate diphosphohydrolase 4 |
| 10 | HMGB1 | High mobility group box 1 |
| 11 | IL17A | Interleukin 17A |
| 12 | IL1B | Interleukin 1 beta |
| 13 | IL6 | Interleukin 6 |
| 14 | ITGAM | Integrin subunit alpha M |
| 15 | MAPK1 | Mitogen-activated protein kinase 1 |
| 16 | MAPK3 | Mitogen-activated protein kinase 3 |
| 17 | MMP9 | Matrix metallopeptidase 9 |
| 18 | MTOR | Mechanistic target of rapamycin kinase |
| 19 | PADI4 | Peptidyl arginine deiminase 4 |
| 20 | RIPK1 | Receptor interacting serine/threonine kinase 1 |
| 21 | RIPK3 | Receptor interacting serine/threonine kinase 3 |
| 22 | SELP | Selectin P |
| 23 | TLR2 | Toll like receptor 2 |
| 24 | TLR4 | Toll like receptor 4 |
| 25 | TNF | Tumor necrosis factor |
| 26 | ACTB | Actin beta |
| 27 | ACTN4 | Actinin alpha 4 |
| 28 | C3 | Complement C3 |
| 29 | C5AR1 | Complement C5a receptor 1 |
| 30 | CASP1 | Caspase 1 |
| 31 | VCAN | Versican |
| 32 | CCL2 | C-C motif chemokine ligand 2 |
| 33 | CFTR | Cystic fibrosis transmembrane conductance regulator |
| 34 | CTSC | Cathepsin C |
| 35 | CXCR4 | C-X-C motif chemokine receptor 4 |
| 36 | ENO1 | Enolase 1 |
| 37 | GPBAR1 | G-protein coupled bile acid receptor 1 |
| 38 | GSDMD | Gasdermin D |
| 39 | HIF1A | Hypoxia inducible factor 1 subunit alpha |
| 40 | IL1RL1 | Interleukin 1 receptor like 1 |
| 41 | IL33 | Interleukin 33 |
| 42 | IL5 | Interleukin 5 |
| 43 | ILK | Integrin linked kinase |
| 44 | IRF1 | Interferon regulatory factor 1 |
| 45 | ITGB1 | Integrin subunit beta 1 |
| 46 | KLF2 | Kruppel like factor 2 |
| 47 | LYZ | Lysozyme |
| 48 | MFN1 | Mitofusin 1 |
| 49 | MFN2 | Mitofusin 2 |
| 50 | MIR21 | MicroRNA 21 |
| 51 | MIR223 | MicroRNA 223 |
| 52 | MYD88 | MYD88 innate immune signal transduction adaptor |
| 53 | MYH9 | Myosin heavy chain 9 |
| 54 | NFE2L2 | NFE2 like bZIP transcription factor 2 |
| 55 | NFKBIA | NFKB inhibitor alpha |
| 56 | NLRP3 | NLR family pyrin domain containing 3 |
| 57 | NOX4 | NADPH oxidase 4 |
| 58 | ORAI1 | ORAI calcium release-activated calcium modulator 1 |
| 59 | PKM | Pyruvate kinase M1/2 |
| 60 | S100A8 | S100 calcium binding protein A8 |
| 61 | S100A9 | S100 calcium binding protein A9 |
| 62 | SOCS3 | Suppressor of cytokine signaling 3 |
| 63 | SPP1 | Secreted phosphoprotein 1 |
| 64 | STAT3 | Signal transducer and activator of transcription 3 |
| 65 | SUCNR1 | Succinate receptor 1 |
| 66 | SYK | Spleen associated tyrosine kinase |
| 67 | TNFAIP3 | TNF alpha induced protein 3 |
| 68 | JAK2 | Janus kinase 2 |
| 69 | FRAS1 | Fraser extracellular matrix complex subunit 1 |
| 70 | CASP3 | Caspase 3 |
| 71 | CRP | C-reactive protein |
| 72 | EGFR | Epidermal growth factor receptor |
| 73 | IL10 | Interleukin 10 |
| 74 | CD36 | CD36 molecule |
| 75 | CXCL5 | C-X-C motif chemokine ligand 5 |
| 76 | VEGFA | Vascular endothelial growth factor A |
| 77 | FBN1 | Fibrillin 1 |
| 78 | IFNG | Interferon gamma |
| 79 | ESM1 | Endothelial Cell-Specific Molecule 1 |
| 80 | FN1 | Fibronectin 1 |
| 81 | IL1A | Interleukin 1 alpha |
| 82 | ICAM1 | Intercellular adhesion molecule 1 |
| 83 | TGFB1 | Transforming growth factor beta 1 |
| 84 | MMP2 | Matrix metallopeptidase 2 |
| 85 | SOD3 | Superoxide dismutase 3 |
| 86 | MEFV | MEFV innate immunity regulator |
| 87 | LCN2 | Lipocalin 2 |
| 88 | CXCL8 | C-X-C motif chemokine ligand 8 |
| 89 | TNFRSF1A | TNF receptor superfamily member 1A |

**2.6 Table S6: Sensitivity analysis of NET gene-set enrichment (bulk GSEA).**

| Gene set | Bulk GSEA p.adj | Interpretation |
| --- | --- | --- |
| DKD - NRGs | 0.023 | Significant — DKD - contextualized NET signal |
| KEGG NET formation | 0.098 | NS (trend only) |
| GO NET formation | 0.982 | NS |
| WikiPathways NETs | 0.441 | NS |

**2.7** **Table S7: Primer sequences for qRT-PCR.**

| Gene name |  |  | Sequence (5’->3’) |
| --- | --- | --- | --- |
| VCAN | Mus musculus | F | GAAGGCTTGTTTGGACGTTGG |
|  |  | R | ACGGAATCCATAAGTCCTGACTC |
| ESM1 | Mus musculus | F | TGGACTGCCCTCAACACTG |
|  |  | R | CCTGAGACTGTGCGGTAGC |

### 2.8 Table S8:Molecular docking prediction results.

| **Gene Symbol** | **Drug ID (ZINC)** | **BindingDB Prediction Score** | **Vina Score (kcal/mol)** | **Interacting Residues** |
| --- | --- | --- | --- | --- |
| VCAN | ZINC005331 | 0.802 | -9.6 | TYR-326, THR-324, ARG-305, HIS-153 |
|  | ZINC070181 | 0.716 | -7.9 | GLN-284, VAL-322 |
|  | ZINC139471 | 0.646 | -7.8 | HIS-153, GLN-195, GLN-331, THR-324 |
|  | ZINC176945 | 0.655 | -7.3 | ASP-191, GLU-194 |
|  | ZINC097413 | 0.652 | -6.8 | GLN-284 |
| ESM1 | ZINC171091 | 0.903 | -6.6 | SER-119, GLN-118 |
|  | ZINC185772 | 0.89 | -6.8 | SER-119, GLN-118 |
|  | ZINC236913 | 0.835 | -6.8 | GLN-118, SER-119 |
|  | ZINC112643 | 0.818 | -6.8 | GLN-136, PHE-132 |
|  | ZINC069604 | 0.861 | -6.3 | PHE-132, SER-119, GLN-118 |
